# Reactions of Nickel(0)–Olefin Pincer Complexes with Terminal Alkynes: Cooperative C–H Bond Activation and Alkyne Coupling

**DOI:** 10.1021/acs.organomet.3c00404

**Published:** 2023-11-22

**Authors:** María L. G. Sansores-Paredes, Tú T. T. Nguyen, Martin Lutz, Marc-Etienne Moret

**Affiliations:** † Organic Chemistry and Catalysis, Institute for Sustainable and Circular Chemistry, Faculty of Science, Utrecht University, Universiteitsweg 99, 3584 CG Utrecht, The Netherlands; ‡ Structural Biochemistry, Bijvoet Centre for Biomolecular Research, Faculty of Science, Utrecht University, Universiteitsweg 99, 3534 CG Utrecht, The Netherlands

## Abstract

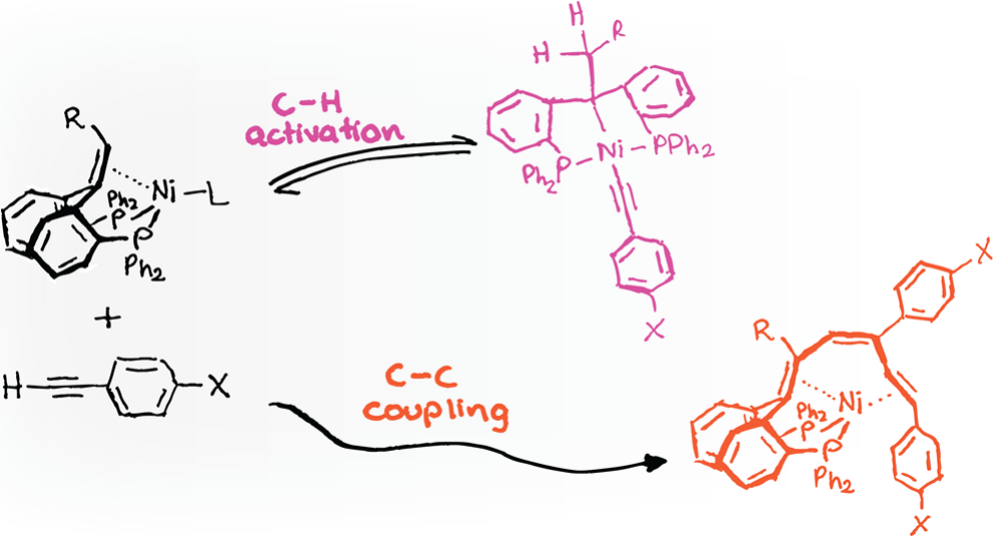

Metal–ligand cooperation can facilitate the activation of chemical bonds, opening reaction pathways of interest for catalyst development. In this context, olefins occupying the central position of a diphosphine pincer ligand (PC=CP) are emerging as reversible H atom acceptors, e.g., for H_2_ activation. Here, we report on the reactivity of nickel complexes of PC=CP ligands with a terminal alkyne, for which two competing pathways are observed. First, cooperative and reversible C–H bond activation generates a Ni(II) alkyl/alkynyl complex as the kinetic product. Second, in the absence of a bulky substituent on the olefin, two alkyne molecules are incorporated in the ligand structure to form a conjugated triene bound to Ni(0). The mechanisms of these processes are studied by density functional theory calculations supported by experimental observations.

## Introduction

**Figure 1:**
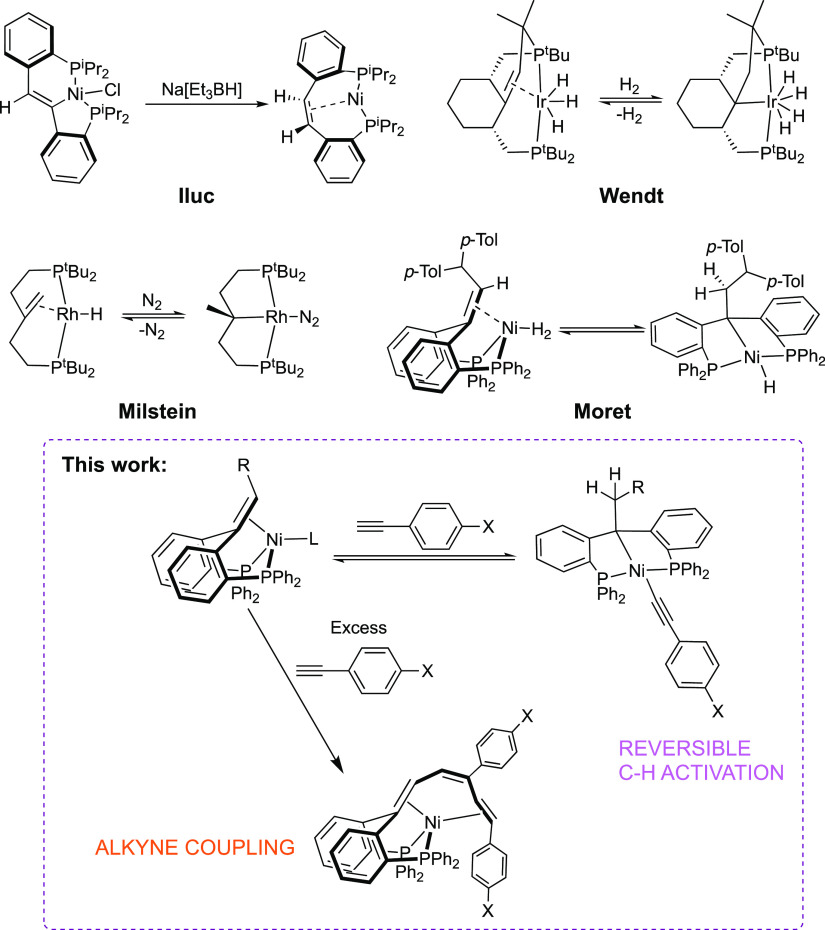
Examples of reported hydrogen transfer reactions of metal complexes featuring PC=CP pincer ligands.

Metal–ligand cooperativity is a promising strategy for the development of efficient metal catalysts.^1,2^ It employs ligands that take an active part in the elementary steps of catalysis in several ways: redox noninnocence (accepting or releasing of electrons), adaptivity (adapting the coordination mode/number of the metal to stabilize intermediate states), and bifunctional bond activation (bond making/cleavage involving ligand atoms).^1−5^ Pincer ligands are a prominent platform for metal–ligand cooperativity owing to their robustness and versatile reactivity, such as reversible hydrogen transfer.^3,6−12^ In recent years, pincer ligands featuring a π-acceptor moiety such as an olefin in the central position have attracted attention.^3,13−28^ In particular, olefin diphosphine pincer ligands (PC=CP) have been shown to engage in various hydrogen atom transfer reactions (Figure 1). Iluc reported the synthesis of nickel complexes featuring an olefin diphosphine pincer ligand derived from stilbene.^18^ Complexation with (dme)NiCl_2_ leads to the activation of the olefinic C–H bond, yielding a square planar (vinyl)nickel(II)Cl complex. Exposing this complex to a hydride source leads to hydrogen transfer to the backbone, yielding the nickel(0)–olefin complex. Milstein observed an intriguing reversible *trans*-hydride insertion of an olefin(hydrido)rhodium(I) complex induced by N_2_.^25^ Wendt described the reversible formation of an alkyl tetrahydride iridium complex by exposing an olefin trihydride iridium complex to molecular H_2_.^27^ Recently, we reported the reversible bifunctional activation of molecular H_2_ by a nickel(0)–olefin complex via ligand-to-ligand hydrogen transfer (LLHT) and its catalytic activity in the semihydrogenation of diphenylacetylene.^28^

Seeking to expand the latter activation mechanism beyond H_2_, we herein describe the reactivity of nickel(0)–olefin pincer complexes toward terminal alkynes. Alkynes are valuable organic substrates, with their polyunsaturated nature allowing for the construction of complex carbon backbones (including polymers) and further functionalization by addition reactions.^29,30^ Because nickel has been proposed to facilitate C–H bond activation steps via LLHT, we anticipated that similar processes could take place with terminal alkynes.^28,31−33^ The uncovered reactivity includes two competing pathways. The anticipated C–H bond activation yielding a Ni(II) square planar complex is found to be rapid and reversible; over time, a slower C–C alkene/alkyne coupling reaction affords Ni(0) triene complexes as the thermodynamic products. The cooperative role of the olefin moiety in C–H bond activation and the mechanism of the C–C coupling reaction are investigated by computational methods.

## Results and Discussion

**Scheme 1:**
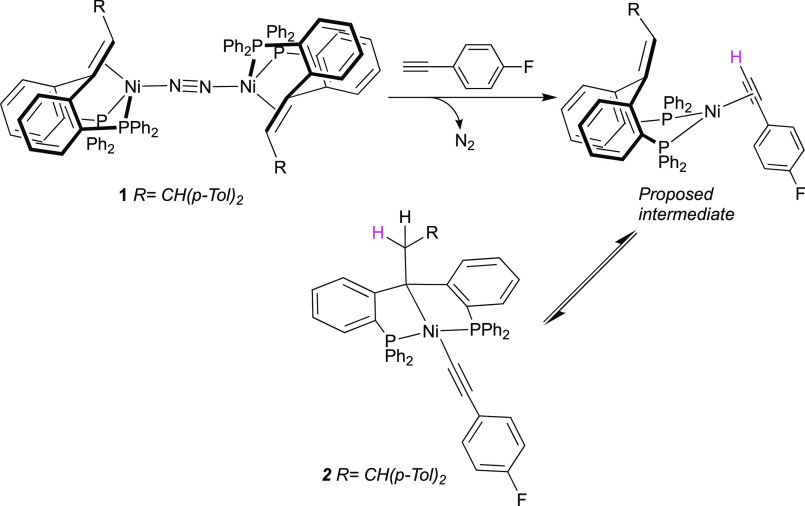
Reactivity of a Bulky Nickel(0)–Olefin Complex Toward 4-Ethynyl-1-fluorobenzene

The synthesis of the complex (^Ph^bppe^H,CH*p*-*T*ol_2_^)Ni(N_2_) (**1**, Scheme 1), featuring a bulky trisubstituted olefin backbone and an easily displaceable N_2_ ligand, was described previously.^21^ Addition of 1.2 equiv of 1-ethynyl-4-fluorobenzene to a solution of **1** in C_6_D_6_ led to the observation of two species in ^31^P{^1^H} nuclear magnetic resonance (NMR) in an approximately 93%:7% proportion (see Supporting Information). There were no further changes in the observed species over a period of 16 h.

The main species, complex **2**, was identified by NMR spectroscopy as an alkyl/alkynyl Ni(II) complex resulting from acetylenic C–H bond activation of 1-ethynyl-4-fluorobenzene. In contrast with the two doublets observed for compound **1**, a ^31^P{^1^H} NMR spectrum of **2** displays one singlet at δ = 35.2 ppm, indicating increased symmetry due to the transfer of an H atom to the olefinic backbone. The newly formed C*H*_2_ unit gives rise to an ^1^H NMR doublet signal at δ = 2.99 ppm (*J*_H,H_ = 7.7 Hz) due to coupling with the proton belonging to the C*H*(*p*-Tol)_2_ group. The latter methine proton displays a triplet multiplicity at δ = 4.01 ppm (t, *J*_H,H_ = 7.1 Hz). The corresponding ^13^C{^1^H} NMR shifts are in good agreement with alkyl carbons located at δ = 50 (CH) and 58 ppm (*C*H_2_). Finally, the infrared (IR) spectrum of **2** depicts a characteristic absorption at 2088 cm^–1^ related to the vibration of the triple bond.

The minor species observed in ^31^P{^1^H} NMR presents two doublets at δ 30.5 (d, *J*_P,P_ = 25.8 Hz) and 30.1 ppm (d, *J*_P,P_ = 25.6 Hz), consistent with an intact olefinic backbone. This species is tentatively assigned as an η^2^ (C,C)-complex of the alkyne without coordination of the olefin backbone. The available spectroscopic data is in good agreement with that of similar structures such as (^Ph^bppe^H,CH*p*-*T*ol_2_^)Ni(η^2^ (C,C)-diphenylacetylene) and alkyne complexes of an analogue (PC=OP)Ni(0) fragment, in which the olefin or ketone backbone is not coordinated to the nickel center.^20,28^ Separation attempts were unsuccessful, and the proportion of species in ^31^P{^1^H} NMR was maintained over several samples, suggesting that these two species could be in equilibrium.

**Scheme 2:**
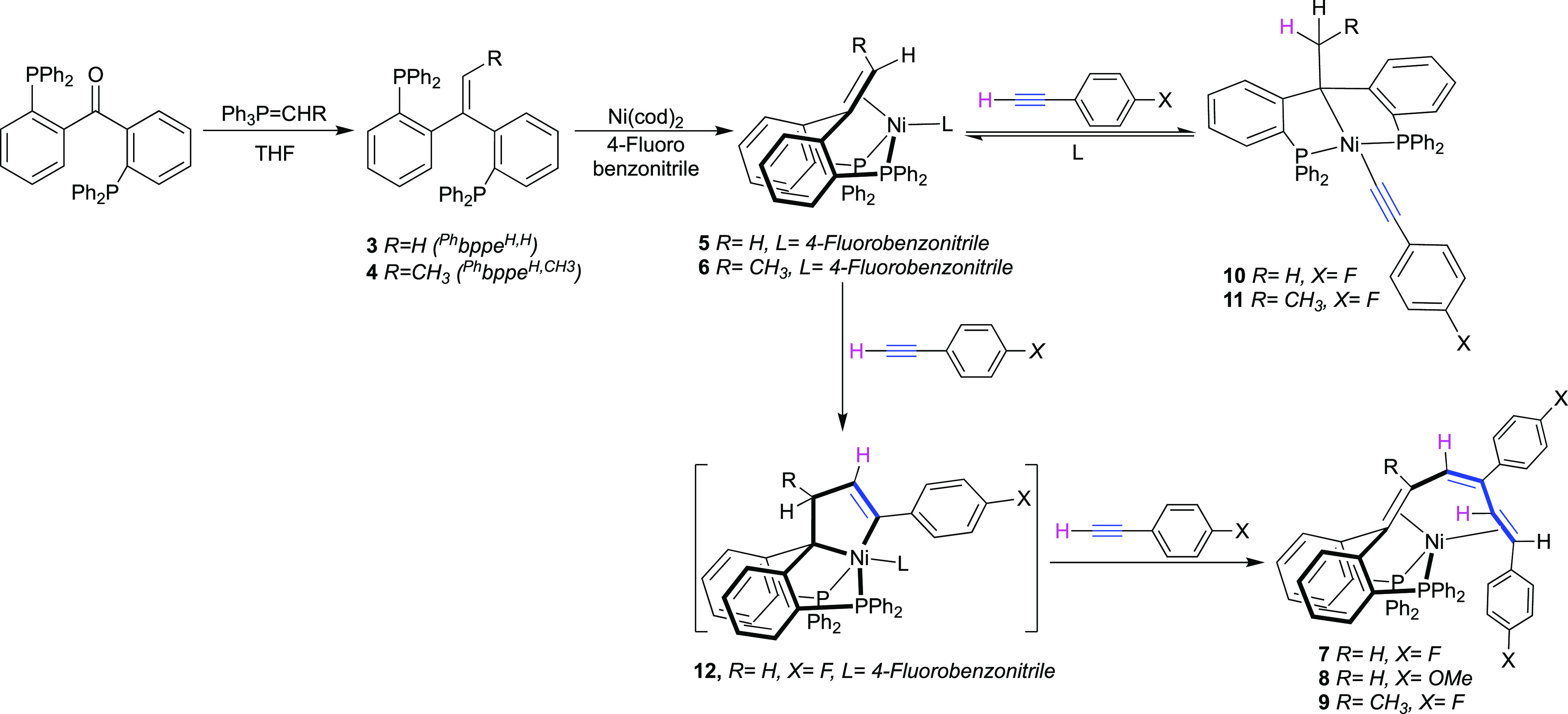
Synthesis and Reactivity of Nickel(0)–Olefin Pincer Complexes

Encouraged by these results, we aimed to explore the effect of the substituent of the olefin backbone on reactivity. The unsubstituted ligand 1,1-bis[2-(diphenylphosphino)phenyl]ethene (^Ph^bppe^H,H^, **3**) was synthesized as previously reported via Wittig reaction from the corresponding ketone (Scheme 2).^21^ The methyl-substituted ligand ^Ph^bppe^H,Me^ (**4**) could be accessed analogously. In contrast to ligand **3**, the ^1^H NMR signal (δ 5.81) corresponding to the olefinic proton in ligand **4** displays long-distance coupling with one of the phosphorus nuclei resulting in a doublet of quadruplets multiplicity (“dq”, *J*_H,H_ = 6,5 Hz, *J*_H,P_ = 3.2 Hz). Confirming this interpretation, the ^1^H{^31^P} NMR spectrum displays the expected quadruplet multiplicity as a result of the coupling with the methyl group. Complexation of **3** and **4** with Ni(cod)_2_ and 4-fluorobenzonitrile is straightforward and yields tetrahedral Ni(0) complexes **5** and **6**, respectively, where the olefin backbone is coordinated to the nickel center (Scheme 2). This is confirmed by the upfield shift of the olefinic ^1^H NMR signals to δ = 3.72 ppm for complex **5** and 3.88 ppm for complex **6** in a C_6_D_6_ solution. The ^31^P{^1^H} NMR spectrum of complex **5** in C_6_D_6_ solution exhibits one singlet at δ 18.5 ppm, while complex **6** gives rise to two doublets at δ 28.5 (d, *J*_P,P_ = 64 Hz) and 9.15 ppm (d, *J*_P,P_ = 64 Hz) as a result of the unsymmetrical substitution of the olefinic backbone.

**Figure 2:**
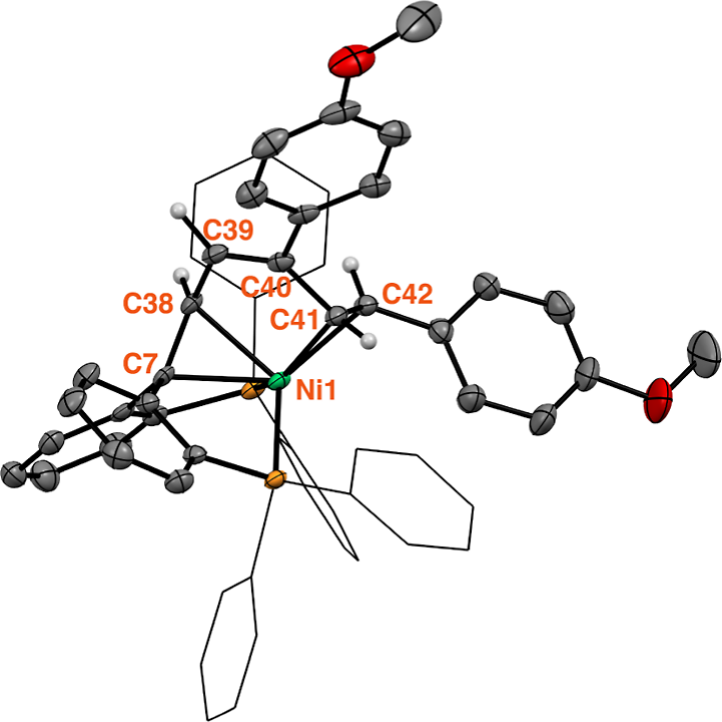
Molecular structure of complex **8**. Displacement ellipsoids are drawn at the 50% probability level. Solvent molecules and most H atoms are omitted for clarity. Phenyl rings of the phosphines and tolyl groups are presented as wireframes. Selected bond lengths (Å): C7–C38 1.413(4), C38–C39 1.468(4), C39–C40 1.332(4), C40–C41 1.496(4), C41–C42 1.398(4), Ni1–C7 2.109(3), Ni1–C38 2.102(3), Ni1–C41 2.022(3), Ni1–C42 2.065(3).^34^

Unexpectedly, treating complex **5** with 1-ethynyl-4-fluorobenzene (2 equiv) for 16 h at room temperature resulted in a different reaction leading to complex **7**, which could be isolated in 90% yield. Complex **7** presents an unsymmetric ^31^P{^1^H} NMR spectrum in C_6_D_6_ with two singlet peaks at δ = 38.7 and 29.1 ppm. Its ^1^H NMR spectrum displays a particular set of peaks integrating for 4 hydrogen atoms that presumably originate from the olefinic CH_2_ group and two molecules of alkyne: δ = 5.58 (dd, *J*_H,H_ = 2.9 Hz, *J*_H,P_ = 8.4 Hz, 1H); 4.39 (q, *J*_H,P_ = 3.9 Hz) and two overlapping peaks at δ = 4.09 ppm (2H). The ^19^F NMR spectrum of **7** displayed two signals at −115.43 and −121.1 ppm. The IR spectrum of **7** displayed no characteristic absorption for a C≡C triple bond. These data collectively suggest that **7** is a condensation product of the (^Ph^bppe^H,H^)Ni fragment with two equiv of alkyne. Because the crystal quality of **7** was not sufficient for an X-ray single-crystal diffraction experiment, we synthesized analogous complex **8** using 1-ethynyl-4-methoxybenzene. Complex **8** presents NMR spectra similar to those of **7** and afforded suitable crystals. X-ray crystal structure determination confirms the incorporation of two alkyne molecules to form a coordinated hexatriene structure (Figure 2).^34^ Complex **8** is best described as a distorted tetrahedral Ni(0) complex, with the terminal double bonds of the hexatriene moiety being coordinated in an η^2^(C,C) fashion. The two phosphines complete the coordination environment of nickel. Both newly formed C=C bonds are found in the (E) conformation generally expected for syn-insertion processes.

Similar to **5**, the reaction of complex **6** with two equivalents of 1-ethynyl-4-fluorobenzene afforded the analogous complex **9**, which exhibits similar NMR characteristics. The ^31^P{^1^H} NMR spectrum displays two doublets at δ 38.9 ppm (*J*_P,P_ = 8.6 Hz) and 27.6 ppm (*J*_P,P_ = 8.4 Hz). The ^1^H NMR spectrum presents one olefinic signal at δ = 5.53 ppm (d, *J*_H,P_ = 7.3 Hz) and two upfield-shifted signals associated with a coordinated olefin at δ = 4.18–4.10 (m) and 3.78 ppm (td, *J*_H,H_ = 11.3, *J*_H,P_ = 2.8 Hz). The methyl group is located at δ 1.34 ppm as a doublet (d, *J*_H,P_ = 10.7 Hz).

These observations show that replacing the bis(*p*-tolyl)methyl substituent in **1** with smaller substituents H or Me opens a new reactive pathway. The olefin moiety acts as an initiator for the coupling of two alkynes at room temperature. To afford final products **7**–**9**, one of the olefinic protons in the starting material has formally migrated to the terminus of the hexatriene chain, preventing further insertions. Indeed, compounds **7**–**9** show no sign of further reaction in the presence of excess corresponding alkyne.

More insights on the mechanism of formation of compounds **7**–**9** were obtained by monitoring the reaction of complex **5** and **6** with 1-ethynyl-4-fluorobenzene (2 equiv) by multinuclear NMR. After 10 min, complexes **5/6** were fully converted to new species **10/11** that then gradually evolved to compounds **7/9**, with the conversion being complete after 16 h. The species **10** and **11** were spectroscopically identified as C–H activation products analogous to **2**. In C_6_D_6_, complex **10** exhibits one ^31^P{^1^H} NMR singlet at δ = 38.3 ppm and a ^1^H NMR singlet signal at δ = 2.08 ppm for the newly formed backbone methyl group. Complex **11** also exhibits a ^31^P{^1^H} NMR singlet at δ = 38.4 ppm, characteristic of a symmetrical species. In the ^1^H NMR spectrum of **11**, the ethyl group of **11** gives rise to the expected quartet (δ 2.36 ppm, *J*_H,H_ = 7.8 Hz) and triplet (δ 1.41 ppm, *J*_H,H_ = 7.4 Hz) for the newly formed CH_2_ and the preexisting CH_3_ group, respectively. Hence, Ni(II) C–H bond activation products **10** and **11** are kinetic products of the reaction of **5** and **6** with a terminal alkyne while coupling products **7**–**9** are the thermodynamic products.

Monitoring reactions with a smaller excess (1.2 equiv) of 1-ethynyl-4-fluorobenzene gave more insight into the competition between C–H bond activation and alkyne coupling (see section 1). Complex **5** was fully converted to complex **10** and a small amount of complex **7** after 10 min. After 6 h, ^1^H NMR revealed the presence of four species in solution in approximately 12%:18%:31%:39% proportions: C–H activation product **10**, the starting complex **5**, coupling product **7,** and a new species **12**, respectively. The latter is proposed to be a nickelacyclopentene intermediate with a 4-fluorobenzonitrile coligand based on its spectroscopic properties (see also Supporting Information Sections 1.1 and 4.2). A doublet ^1^H NMR signal at 2.90 ppm is consistent with an aliphatic methylene group. In the ^31^P{^1^H} NMR spectrum, **12** is associated with a single peak at 17.6 ppm, indicating a symmetrical structure. Importantly, the ^19^F NMR spectrum displays two signals corresponding to **12** at −103.25 and −122.6 ppm corresponding to a coordinated 4-fluorobenzonitrile molecule and one incorporated 4-fluorophenylacetylene molecule, respectively, on the basis of their chemical shift. After 24 h, the four species were still present, but the amount of coupling product **7** and starting olefin complex **5** had increased at the expense of C–H activation product **10** and intermediate nickelacyclopentene **12** (proportion 6%:29%:42%:23% for complex **10**, complex **5**, complex **7,** and **12**, respectively). Repeating the reaction with methyl-substituted complex **6** yielded similar results to those of **5**, but no nickelacyclopentene intermediate could be detected (see Section 1.2). These observations positively demonstrate that the starting olefin complexes **5/6** can be regenerated from the C–H activation products **10/11** under the reaction conditions, suggesting that the latter are not intermediates in the formation of **7/9** but rather products of a distinct, reversible pathway. In addition, the observation of intermediate cyclopentene **12** strongly supports a sequential insertion mechanism over an alternative scenario involving initial coupling of two alkynes followed by trapping of the formed metallacyclopentadiene. This also explains why no alkyne cyclotrimerization is observed, in contrast with the (PC=OP)Ni(0) analogue.^14,20^

**Figure 3:**
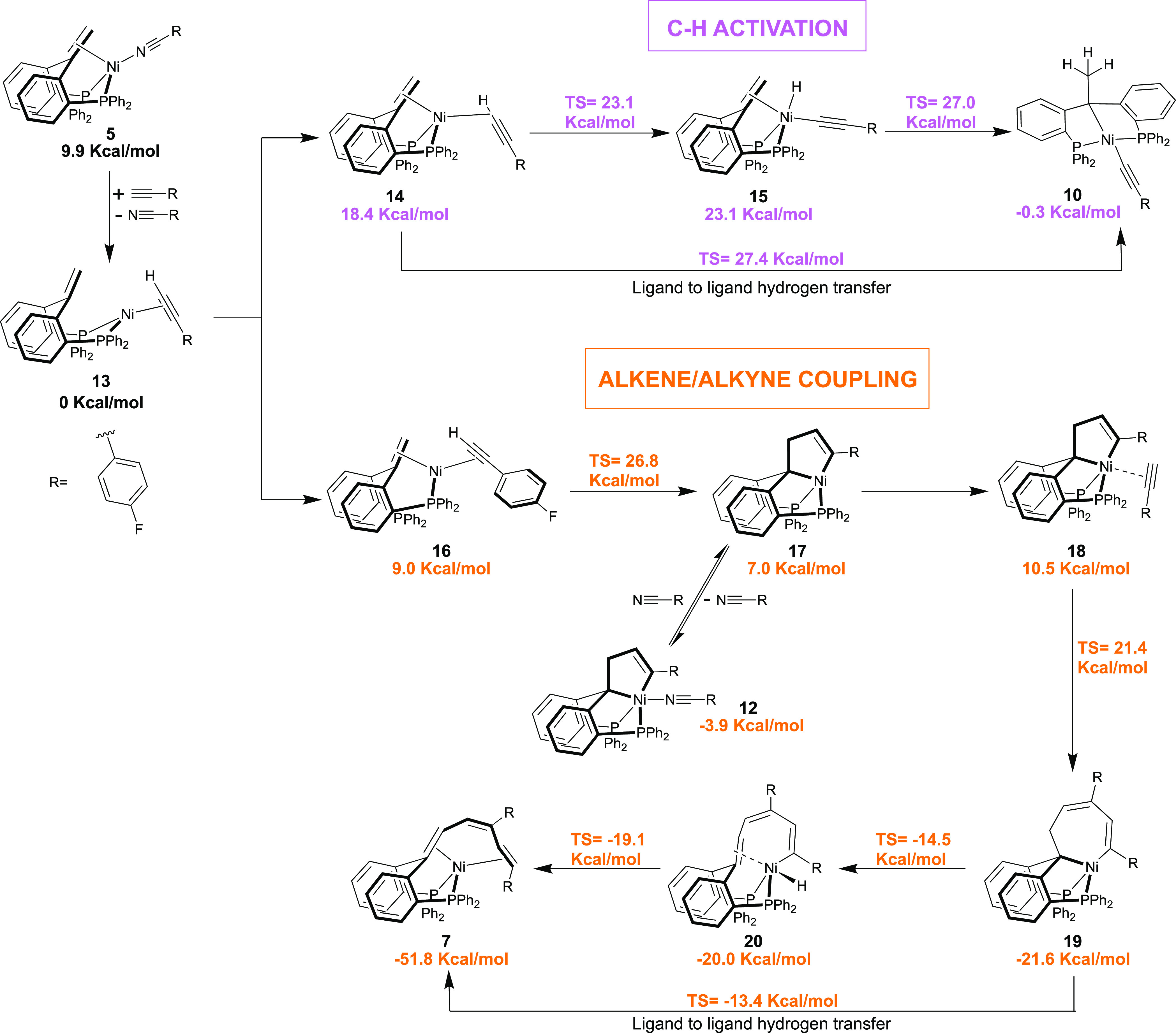
Gibbs free energy profiles for C–H activation and alkene/alkyne coupling processes. Calculations were computed at the B3LYP-GD3BJ/def2TZVP/SMD//B3LYP/6-31g(d,p) level of theory with benzene as solvent.

To gain insight into the mechanism of these processes, DFT calculations were performed on nickel complexes derived from the unsubstituted ligand ^Ph^bppe^H,H^ (**3**) (Figure 3). Ligand substitution from complex **5** to yield complex **13** is exergonic by 9.9 kcal/mol. The structure featuring an alkyne molecule coordinated in the η^2^(C,C) mode without coordination of the olefin backbone was found to be the most stable of the possible isomers of **13**. The C–H bond activation process starts with an endergonic (+18.4 kcal/mol) change in coordination modes to form a σ-complex of the alkyne C–H bond **14** with coordination of the olefin backbone. No isomer of **14** without coordination of the olefin was located. From this point, hydride migration to form the alkyl(alkynyl)nickel(II) complex **10** can follow either a stepwise or a concerted pathway with similar energy barriers. The stepwise process involves oxidative addition of the C–H bond with a transition-state energy of 23.1 kcal/mol to yield the high-lying nickel hydride complex **15** (+23.1 kcal/mol). The olefin backbone then inserts in the Ni–H bond (Δ*G*^‡^ = 27.0 kcal/mol) resulting in complex **10** (−0.3 kcal/mol). The concerted process involves hydrogen migration from σ-complex **14** to complex **10** via LLHT with an overall barrier of 27.4 kcal/mol. The difference in energy between the multistep and concerted processes (0.4 kcal/mol) does not allow us to conclusively favor one over the other (section 4.1). Importantly, the small difference in energy between complex **13** and product complex **10** is in good agreement with an equilibrium process (0.3 kcal/mol). Even though the overall calculated energy barrier above 27 kcal/mol is somewhat high for a reaction taking place at room temperature, the difference is within the uncertainty of the DFT calculations.^35^

The C–C alkene–alkyne coupling pathway leads to a more stable thermodynamic product. First, formation of complex **16** involves a change of coordination by ligand exchange of one of the phosphine arms for the olefinic backbone (+9.0 kcal/mol). Oxidative coupling of the alkyne and the alkene takes place with an overall barrier of 26.8 kcal/mol, yielding a slightly distorted tetracoordinated nickelacyclopentene **17** (+7.0 kcal/mol). Additionally, complex **17** could be stabilized by the reversible coordination of a molecule of 4-fluorobenzonitrile to yield complex **12** (−3.9 kcal/mol), which is in good agreement with the experimental data. The next step is the coordination of a second molecule of alkyne to form complex **18** in a slightly endergonic process (+10.5 kcal/mol). Insertion of the second alkyne molecule with a transition-state energy of 21.4 kcal/mol (25.3 kcal/mol overall barrier from **12**) results in the formation of nickelacycloheptadiene intermediate **19** (−21.6 kcal/mol). From this intermediate, β-hydride elimination is feasible (Δ*G*‡ = −14.5 kcal/mol) yielding (η^2^-olefin)nickel hydride intermediate **20** that subsequently undergoes C–H reductive elimination to generate the triene product **7** with an overall energy gain of −51.8 kcal/mol. Theoretical studies have shown that β-hydride elimination in cycles could be possible if the structure is able to distort to allow the required coplanar conformation.^36^ Additionally, a concerted LLHT transition state from complex **19** to product **7** could be located with an energy of −13.4 kcal/mol. Again, the small energy difference between the two processes (0.9 kcal/mol) does not allow conclusively favoring one of them (see Supporting Information, Section 4.2). The overall barrier of the alkene/alkyne coupling process is 26.8 kcal/mol from complex **13**, which is slightly below the energetic barrier of the C–H bond activation. Nevertheless, the prediction that both processes have similar energetic barriers is consistent with experimental data, where using 2 equiv both the C–H activation and the C–C alkene/olefin coupling products are observed. Another pathway that could potentially lead to the formation of complex **19** starts with initial formation of a nickelacyclopentadiene intermediate (see Supporting Information, Section 4.3).^37−44^ However, the associated energy barrier of 38.8 kcal/mol renders it inaccessible. The formation of the nickelacyclopentene is favored by the competition between intramolecular and intermolecular processes (additional alkyne coordination).

## Conclusions

In summary, we have shown the reactivity of (PC=CP)Ni(0) complexes with terminal alkynes at room temperature. The reactivity with internal alkynes presents two processes: reversible C–H activation and alkene/alkyne coupling. Both processes are observed when the olefin backbone bears small substituents (H or CH_3_), and only C–H activation is observed for the bulky substituent [CH(*p*-Tol)_2_]. The facile and reversible C–H activation reaction demonstrates the bifunctional behavior of the olefin pincer ligand. In the alkene/alkyne coupling, the olefin backbone acts as a substrate by undergoing C–C coupling with two alkyne molecules through a proposed nickelacyclopentene intermediate. DFT calculations suggest that the key hydrogen transfer steps in both mechanisms could occur via concerted LLHT processes.

These results illustrate the potential of olefin pincer ligands as cooperative moieties for further catalyst development in the activation of C–H bonds. In addition, the observation of a sequential coupling reaction of the olefin moiety with two alkyne molecules suggests that such structures could be used as initiators for alkyne oligomerization or polymerization processes.^45−49^ In the present system, further chain growth is prevented by facile intramolecular hydrogen transfer from the ligand backbone that yields a stable 18-electron Ni(0) complex. Circumventing this quenching process, e.g., by using PC=CP ligands with a tetrasubstituted olefin, may ultimately lead to new (catalytic) alkyne oligomerization processes controlled by metal–ligand cooperation.

## Experimental Section

### General Information

All of the reactants were purchased from commercial sources and used as-received without further purification. Additionally, Ni(cod)_2_, 4-fluorobenzonitrile, 1-ethynyl-4-fluorobenzene, and 1-ethynylanisole were stored in a glovebox. All of the reactions were performed under an *N*_2_(g) atmosphere using standard Schlenk line or glovebox techniques. Deuterated solvents were purchased from Cambridge Isotope Laboratory Incorporation (Cambridge, USA), degassed by three freeze–pump–thaw cycles, and stored over molecular sieves before use. Common solvents were dried using an MBRAUN MB SPS-80 purification system, except tetrahydrofuran (THF) that was purified by distillation from a THF/benzophenone/Na suspension. Compounds 2,2′-bis(diphenylphosphino)-benzophenone (^Ph^dpbp), ligand **3,** and complex **1** were synthesized according to literature procedures.^21,22^^1^H, ^13^C, ^19^F, and ^31^P NMR spectra (400, 100, 376, and 161 MHz, respectively) were recorded on an Agilent MR400 or a Varian AS400 spectrometer at 297 K. ^1^H and ^13^C NMR chemical shifts relative to tetramethylsilane are referenced to the residual solvent resonance. ^19^F NMR chemical shifts were referenced to CFCl_3_, and ^31^P NMR chemical shifts were referenced to 85% aqueous H_3_PO_4_ solution, both externally. Infrared spectra were recorded using a Perking Elmer Spectrum One FT-IR spectrometer under a N_2_ flow. The bulk purity of the reported compounds is supported by clean NMR spectra (see Supporting Information). Additionally, elemental analysis was conducted on complex **7** and **8** by Medac Ltd., Surret, United Kingdom.

### Computational Methods

DFT calculations were performed using the Gaussian 16 software package version C.01.^50^ Geometry optimizations were carried out in a vacuum at the B3LYP/6-31g(d,p) level of theory on all atoms. Frequency analyses on all stationary points were used to ensure that they are minima (no imaginary frequency) or transition states (one imaginary frequency). Transition states were calculated using the synchronous transit-guided quasi-Newton number 3 (QST3) method or using the opt = TS (Berny algorithm) keyword. The guess structures for TS calculations were based on the results of relaxed potential energy surface scans. Δ*G*° was calculated by single-point calculation at the B3LYP-GDB3J/def2TZVP/SMD(benzene) level of theory adjusting the value with the thermal correction obtained at the B3LYP/6-31g(d,p) level of theory with a temperature of 298.15 K and a pressure of 1 atm.

### Complex **2**

1.2 equiv of 1-ethynyl-4-fluorobenzene (0.015 mmol, 1.7 μL) was added to a solution of 10 mg of complex **1** (0.006 mmol) in approximately 0.6 mL of C_6_D_6_. The solution was immediately transferred to a J-Young NMR tube. After ca. 10 min, a ^1^H NMR spectrum showed full conversion, and all NMR data were recorded. To obtain an IR spectrum, the solvent was evaporated, and the resulting solid was washed four times with 0.3 mL of hexane to yield a red solid (4 mg, 40%).

^1^H NMR (400 MHz, C_6_D_6_, 25 °C): δ(ppm) 7.89 (d, *J* = 5.7 Hz, 4H, Ar–*H*), 7.53 (d, *J* = 8.1 Hz, 2H, Ar–*H*), 7.45–7.36 (m, 4H, Ar–*H*), 7.33–7.25 (m, 2H, Ar–*H*), 7.22–7.17 (m, 6H, Ar–*H*), 7.12 (d, *J* = 7.8 Hz, 2H, Ar–*H*), 7.01 (d, *J* = 8.0 Hz, 4H, Ar–*H*), 6.93 (t, *J* = 6.6 Hz, 6H, Ar–*H*), 6.87 (t, *J* = 7.2 Hz, 8H, Ar–*H*), 6.66 (t, *J* = 8.8 Hz, 2H, Ar–*H*), 4.01 (t, *J*_H–H_ = 7.1 Hz, 1H, C*H*), 2.99 (d, *J*_H–H_ = 7.7 Hz, 2H, C*H*_2_), 2.14 (s, 6H, C*H*_3_).

^31^P{^1^H} NMR (161 MHz, C_6_D_6_, 25 °C): δ(ppm) 35.2 (s, 2P).

^19^F NMR (376 MHz, C_6_D_6_, 25 °C): δ(ppm) −115.76––119.51 (m, 1F).

^13^C{^1^H} NMR (100 MHz, C_6_D_6_, 25 °C): δ 163.3 (t, *J* = 19.5 Hz, Ar), 161.9 (s, Ar), 159.5 (s, Ar), 144.1 (s, Ar), 139.5 (t, *J* = 20.0 Hz, Ar), 137.4–135.6 (m, Ar), 135.1 (s, Ar), 134.8 (d, *J* = 12.2 Hz, Ar), 134.3 (s, Ar), 133.6 (t, *J* = 5.8 Hz, Ar), 131.9 (s, Ar), 130.4 (s, Ar), 130.0 (s, Ar), 129.1 (s, Ar), 128.5 (t, *J* = 4.8 Hz, Ar), 128.4 (s, Ar), 127.3 (s, Ar), 126.5 (d, *J* = 3.3 Hz, Ar), 119.9 (s, Ar), 114.9 (d, *J* = 21.4 Hz, Ar), 112.5 (t, *J* = 35 Hz, C_alkyne_ or Ar) 65.6 (t, *J* = 8.8 Hz, *C*–CH_2_–CH), 58.0 (s, *C*H_2_–CH), 50.9 (d, *J* = 7.5 Hz, CH_2_–*C*H), 21.1 (s, *C*H_3_).

IR (cm^–1^): 3055, 2973, 2919, 1855, 2088, 1578, 1497, 1483, 1433, 1204, 1095, 1066, 1026, 911, 831, 742, 692, 519.

The high sensitivity of this compound did not allow us to obtain elemental analysis data.

### Ligand **4** (^Ph^bppe^H,Me^)

A 2.7 g portion of ethyltriphenylphosphonium bromide (EtTPPBr, 0.007 mol) was suspended in 40 mL of THF. Under constant stirring, 4.8 mL of *n*-BuLi (0.007 mol, 1.6 M in hexanes) was added dropwise, and the mixture was stirred for 30 min. 1.0 g of dpbp (0.0018 mol) was suspended in 30 mL of THF and added dropwise to the reaction mixture over 10 min. The reaction was heated to reflux for 16 h. After that, 50 mL of saturated NaHCO_3_ aqueous solution and 50 mL of Et_2_O were added. The organic phase was separated by decantation and washed with 50 mL of brine. The organic phase was dried over MgSO_4_ and concentrated under a vacuum to obtain a yellow powder. The solid was recrystallized in MeOH. The resulting powder was dried under vacuum yielding (700 mg, 69%).

^1^H NMR (400 MHz, C_6_D_6_, 25 °C): δ(ppm) 7.58–7.47 (m, 5H, Ar–*H*), 7.44–7.30 (m, 6H, Ar–*H*), 7.09–6.99 (m, 13H, Ar–*H*), 6.99–6.92 (m, 2H, Ar–*H*), 6.89 (td, *J* = 7.5, 1.5 Hz, 1H, Ar–*H*), 5.81 (qd, *J*_H–H_ = 6.9, *J*_H–P_ = 3.2 Hz, 1H, =C*H*), 1.40 (d, *J*_H–H_ = 6.9 Hz, 3H, C*H*_3_).

^31^P{^1^H} NMR (161 MHz, C_6_D_6_, 25 °C): δ(ppm) −12.77 (d, *J*_P–P_ = 24.0 Hz), −14.95 (d, *J*_P–P_ = 24.1 Hz).

^13^C{^1^H} NMR (100 MHz, C_6_D_6_, 25 °C) δ(ppm): δ 150.3 (d, *J* = 28.2 Hz, Ar), 147.6 (d, *J* = 32.4 Hz, Ar), 141.3 (t, *J* = 6.1 Hz, Ar), 138.6 (d, *J* = 13.7 Hz, Ar), 138.4 (dd, *J* = 15.3, 2.3 Hz, Ar), 136.8 (d, *J* = 2.4 Hz, Ar), 136.8–136.4 (m, Ar or *C*=CH), 135.4 (d, *J* = 2.2 Hz, Ar), 134.6–133.5 (m, Ar), 132.6 (dd, *J* = 8.4, 1.9 Hz, Ar), 131.8 (dd, *J* = 7.0, 4.1 Hz, Ar or =*C*H), 130.5–130.0 (m, Ar or =*C*H), 129.1 (s, Ar), 128.7 (d, *J* = 6.1 Hz, Ar), 128.6 (d, *J* = 6.4 Hz, Ar), 128.5 (d, *J* = 2.2 Hz, Ar), 127.7 (s, Ar), 126.8 (s, Ar), 15.7 (d, *J* = 2.2 Hz, Me). Some signals are obscured by the residual solvent peak.

IR (cm^–1^): 3069, 3049, 3001, 1581, 1474, 1460, 1432, 1090, 1028, 768, 768, 743, 695, 505, 494.

### Complex **5**

300 mg of **3**, ^Ph^bppe^H,H^ (0.547 mmol), and 150 mg of Ni(cod)_2_ (0.547 mmol) were suspended in 8 mL of toluene. 69 mg of 4-fluorobenzonitrile (0.57 mmol) dissolved in 5 mL of toluene was added slowly. The solution was stirred for 4 h, concentrated down to 2 mL under vacuum, and cooled down to −35 °C. One mL of cold hexane was added, and the solution was kept at −35 °C for 30 min. The precipitate was washed with cold hexane and dried to yield a red, dark solid (260 mg, 65%).

^1^H NMR (400 MHz, C_6_D_6_, 25 °C): δ(ppm) 7.92 (q, *J* = 5.9 Hz, 6H, Ar–*H*), 7.29–7.22 (m, 2H, Ar–*H*), 7.13 (s, 3H, Ar–*H*), 7.07 (t, *J* = 7.6 Hz, 3H, Ar–*H*), 6.95 (d, *J* = 7.2 Hz, 2H, Ar–*H*), 6.89 (q, *J* = 8.1 Hz, 7H, Ar–*H*), 6.65 (dd, *J* = 8.4, 5.2 Hz, 2H, Ar–*H*), 6.28 (t, *J* = 8.4 Hz, 2H, Ar–*H*), 3.72 (s, 2H, =C*H*_2_). Some aromatic signals are obscured by the residual solvent peak.

^31^P{^1^H} NMR (161 MHz, C_6_D_6_, 25 °C): δ(ppm) 18.5 (s, 2P).

^19^F NMR (376 MHz, C_6_D_6_, 25 °C): δ(ppm) −104.0––107.0 (m).

^13^C{^1^H} NMR (100 MHz, C_6_D_6_, 25 °C): δ(ppm) 164.9 (s, Ar), 162.4 (s, Ar), 156.3 (s, Ar), 141.9–141.1 (m, Ar), 140.1 (t, *J* = 8.6 Hz, Ar), 138.9 (t, *J* = 13.7 Hz, Ar), 133.6 (d, *J* = 7.9 Hz, Ar), 133.5 (d, *J* = 6.1 Hz, Ar), 133.2 (dt, *J* = 9.0, 2.6 Hz, Ar), 132.5 (t, *J* = 7.0 Hz, Ar), 128.4 (dd, *J* = 8.0, 3.9 Hz, Ar), 127.3 (s, Ar), 126.2 (t, *J* = 2.0 Hz, Ar), 122.9 (t, *J* = 5.7 Hz, Ar), 116.4 (d, *J* = 22.6 Hz, Ar), 110.8 (d, *J* = 2.7 Hz, Ar), 95.4 (t, *J* = 6.3 Hz, *C*=CH_2_), 59.1 (t, *J* = 8.6 Hz, C=*C*H_2_). Some signals are obscured by the solvent peak.

IR (cm^–1^): 3049, 2973, 2973, 2928, 2855, 2185, 1581, 1502, 1478, 1432, 1235, 1157, 1066, 837, 741, 694, 512, 503.

The high sensitivity of this compound did not allow us to obtain elemental analysis data.

### Complex **6**

The same procedure as for complex **5** was followed using compound **4** (^Ph^bppe^H,CH3^) instead of **3**. 212 mg (53%) of a red solid was obtained.

^1^H NMR (400 MHz, C_6_D_6_, 25 °C): δ(ppm) 7.90 (dt, *J* = 17.0, 7.9 Hz, 5H, Ar–*H*), 7.34–7.25 (m, 3H, Ar–*H*), 7.01 (dt, *J* = 15.3, 7.6 Hz, 8H, Ar–*H*), 6.89 (t, *J* = 7.6 Hz, 5H, Ar–*H*), 6.82 (dt, *J* = 9.1, 4.5 Hz, 4H, Ar–*H*), 6.74 (dd, *J* = 8.6, 5.4 Hz, 2H, Ar–*H*), 6.31 (t, *J* = 8.5 Hz, 2H, Ar–*H*), 3.88 (p, *J*_H–H_ = 6.2 Hz, *J*_H–P_ = 6.2 Hz, 1H, =C*H*), 1.66 (dt, *J*_H–P_ = 6.3 Hz, *J*_H–H_ = 3.2 Hz, 3H, Me). Some aromatic signals are obscured by the residual solvent peak.

^31^P{^1^H} NMR (161 MHz, C_6_D_6_, 25 °C): δ(ppm) 28.5 (d, *J*_P–P_ = 64.1 Hz, 1P), 9.15 (d, *J*_P–P_ = 64.2 Hz, 1P).

^19^F NMR (376 MHz, C_6_D_6_, 25 °C): δ(ppm) −105.9 (m, 1F).

^13^C{^1^H} NMR (100 MHz, C_6_D_6_, 25 °C) δ(ppm): δ 164.8 (s, Ar), 162.3 (s, Ar), 156.9 (d, *J* = 39.7 Hz, Ar), 153.4 (d, *J* = 42.3 Hz, Ar), 147.3 (dd, *J* = 36.4, 6.3 Hz, Ar or *C*N), 141.3 (d, *J* = 10.3 Hz, Ar), 139.7 (dd, *J* = 17.4, 9.7 Hz, Ar), 139.3 (dd, *J* = 17.4, 12.0 Hz, Ar), 138.9 (s, Ar), 137.2 (d, *J* = 29.0 Hz, Ar), 134.6 (s, Ar), 133.7 (d, *J* = 15.2 Hz, (s, Ar)), 133.4–133.0 (m, Ar), 132.8 (d, *J* = 8.7 Hz, Ar), 132.6 (s, Ar), 132.1 (d, *J* = 13.3 Hz, Ar), 130.3 (d, *J* = 14.5 Hz, Ar), 128.6 (s, Ar), 128.5 (d, *J* = 4.0 Hz, Ar), 128.4 (s, Ar), 127.3–127.0 (m, Ar), 125.6 (d, *J* = 12.1 Hz, Ar), 125.2 (d, *J* = 3.1 Hz, Ar), 121.4 (d, *J* = 6.9 Hz, Ar), 116.5 (d, *J* = 22.7 Hz, Ar), 111.1 (s, Ar), 103.7–97.5 (m, *C*=CH), 70.7 (dd, *J* = 10.4, 7.4 Hz, =*C*H), 20.3 (d, *J* = 4.6 Hz, Me). Some aromatic signals are obscured by the solvent peak.

IR (cm^–1^): 3047, 2973, 2928, 1855, 2188, 1584, 1502, 1432, 1235, 1196, 1065, 912, 838, 741, 698, 540.

The high sensitivity of this compound did not allow us to obtain elemental analysis data.

### Complex **7**

A 100 mg portion of complex **5** (0.14 mmol) and 32 μL of 1-ethynyl-4-fluorobenzene (0.28 mmol) were dissolved in 10 mL of toluene. The solution was stirred for 16 h. The solution was concentrated down to 2 mL under vacuum, and 4 mL of hexane was added inducing precipitation. After 15 min, the solid was filtered and washed with cold hexane and dried to yield a red dark solid (107 mg, 90%).

^1^H NMR (400 MHz, C_6_D_6_, 25 °C): δ(ppm) 7.57 (dd, *J* = 7.7, 3.1 Hz, 1H, Ar–*H*), 7.49 (dq, *J* = 17.5, 7.2 Hz, 5H, Ar–*H*), 7.41–7.32 (m, 2H, Ar–*H*), 7.22 (q, *J* = 5.5 Hz, 2H, Ar–*H*), 7.09 (td, *J* = 7.8, 3.8 Hz, 3H, Ar–*H*), 6.96 (qd, *J* = 6.0, 3.6 Hz, 10H, Ar–*H*), 6.81 (dt, *J* = 19.4, 7.2 Hz, 3H, Ar–*H*), 6.66 (ddt, *J* = 9.2, 5.8, 3.5 Hz, 7H, Ar–*H*), 6.49 (td, *J* = 7.8, 1.8 Hz, 2H, Ar–*H*), 5.58 (dd, *J*_H–P_ = 8.4, *J*_H–H_ = 2.9 Hz, 1H, =CH–C*H*=C–CH=), 4.39 (q, *J* = 3.9 Hz, 1H, =C*H*–CH=C–CH=), 4.16–4.00 (m, 2H, CH=C–C*H*=C*H*). Some aromatic signals are obscured by the residual solvent peak.

^31^P{^1^H} NMR (161 MHz, C_6_D_6_, 25 °C): δ(ppm) 38.7 (s, 1P), 29.0 (s, 1P).

^19^F NMR (376 MHz, C_6_D_6_, 25 °C): δ(ppm) −115.43 (td, *J* = 8.6, 4.3 Hz), −121.1 (p, *J* = 6.7 Hz).

^13^C{^1^H} NMR (100 MHz, C_6_D_6_, 25 °C): δ(ppm) 164.1 (s, Ar), 161.5 (d, *J* = 34.7 Hz, Ar), 158.9 (s, Ar), 156.8 (d, *J* = 29.0 Hz, Ar), 151.9 (d, *J* = 38.1 Hz, Ar), 147.9 (d, *J* = 5.7 Hz, Ar), 146.9 (d, *J* = 6.5 Hz, Ar), 146.5 (s, Ar), 144.5 (s, Ar), 140.1 (dd, *J* = 32.8, 7.6 Hz, Ar), 138.2 (d, *J* = 25.2 Hz, Ar), 136.7 (d, *J* = 25.2 Hz, Ar), 136.3 (s, Ar), 135.9 (s, Ar), 135.4 (d, *J* = 16.0 Hz, Ar), 134.2 (d, *J* = 33.2 Hz, Ar), 133.4 (d, *J* = 13.2 Hz, Ar), 132.6 (dd, *J* = 11.5, 3.3 Hz, Ar), 132.4 (s, Ar), 131.4, 130.2 (d, *J* = 14.0 Hz, Ar), 129.7 (s, Ar), 129.0–128.7 (m, Ar), 128.6 (d, *J* = 6.1 Hz, Ar), 127.7 (d, *J* = 8.8 Hz, Ar), 127.2 (d, *J* = 4.7 Hz, Ar), 126.2 (dd, *J* = 8.4, 5.0 Hz, Ar), 125.4 (d, *J* = 4.1 Hz, Ar), 124.5 (d, *J* = 6.8 Hz, =CH–*C*H=C–CH=CH), 115.9–114.1 (m, Ar), 98.7 (d, *J* = 16.4 Hz, *C*=CH–CH=C–CH=), 89.2 (d, *J* = 14.4 Hz, =*C*H–CH=C–CH=), 78.9 (d, *J* = 16.8 Hz, CH=C–CH=*C*H or CH=C–*C*H=*C*H), 69.3 (d, *J* = 5.3 Hz, CH=C–CH=*C*H or CH=C–*C*H=*C*H). Some aromatic signals are obscured by the residual solvent peak.

IR (cm^–1^): 3049, 2973, 2928, 2857, 1581, 1505, 1480, 1457, 1432, 1225, 1155, 1093, 1067, 824, 743, 695, 520.

Elemental analysis: C_54_H_40_NiF_2_P_2_ calcd: C, 76.53%; H 4.76%. Found: C, 75.45%; H, 4.59%.

### Complex **8**

The same procedure as for complex **7** was followed using 20 mg of complex **5** (0.028 mmol) and 7.3 μL of 1-ethynylanisole (0.056 mmol). The product was obtained as a red powder (23 mg, 95%). Crystals suitable for X-ray determination were obtained by the slow vapor diffusion of hexane to a saturated THF solution.

^1^H NMR (400 MHz, C_6_D_6_, 25 °C): δ(ppm) 7.69–7.57 (m, 3H, Ar–*H*), 7.56–7.40 (m, 3H, Ar–*H*), 7.37–7.31 (m, 2H, Ar–*H*), 7.30–7.25 (m, 1H, Ar–*H*), 7.25–7.18 (m, 2H, Ar–*H*), 7.18–7.11 (m, 5H, Ar–*H*), 7.11–7.05 (m, 1H, Ar–*H*), 7.05–6.93 (m, 9H, Ar–*H*), 6.82 (dd, *J* = 8.5, 6.5 Hz, 4H, Ar–*H*), 6.65 (ddd, *J* = 19.0, 8.1, 2.0 Hz, 5H, Ar–*H*), 6.52 (td, *J* = 7.7, 1.7 Hz, 2H, Ar–*H*), 5.70 (dd, *J*_H–P_ = 8.8, *J*_H–H_ = 2.9 Hz, 1H, =CH–C*H*=C–CH=), 4.46 (q, *J* = 3.9 Hz, 1H, =C*H*–CH=C–CH=), 4.40–4.20 (m, 2H, CH=C–C*H*=C*H*), 3.37 (s, 3H, O*Me*), 3.26 (s, 3H, O*Me*). Some aromatic signals are obscured by the residual solvent peak.

^31^P{^1^H} NMR (161 MHz, C_6_D_6_, 25 °C): δ(ppm) 39.3 (s, 1P), 28.0 (s, 1P).

^13^C{^1^H} NMR (100 MHz, C_6_D_6_, 25 °C): δ(ppm) 159.6 (s, Ar), 157.1 (d, *J* = 29.5 Hz, Ar), 156.7 (s, Ar), 152.3 (d, *J* = 40.1 Hz, Ar), 148.9 (d, *J* = 7.4 Hz, Ar), 147.3 (d, *J* = 7.0 Hz, Ar), 146.9 (d, *J* = 7.0 Hz, Ar), 141.2 (s, Ar), 140.5 (d, *J* = 8.1 Hz, Ar), 140.2 (d, *J* = 8.1 Hz, Ar), 138.6 (dd, *J* = 22.7, 1.9 Hz, Ar), 137.3 (d, *J* = 23.7 Hz, Ar), 136.2 (dd, *J* = 21.2, 2.5 Hz, Ar), 135.3 (d, *J* = 16.0 Hz, Ar), 134.2 (d, *J* = 32.8 Hz, Ar), 133.4 (d, *J* = 13.3 Hz, Ar), 132.8 (s, Ar), 132.7 (dd, *J* = 5.8, 4.0 Hz, Ar), 131.5 (s, Ar), 130.4 (d, *J* = 13.8 Hz, Ar), 129.3 (d, *J* = 1.9 Hz, Ar), 128.8–128.4 (m, Ar), 127.6 (d, *J* = 8.6 Hz, Ar), 127.1 (d, *J* = 4.7 Hz, Ar), 126.3 (s, Ar), 126.2 (d, *J* = 9.6 Hz), 125.1 (d, *J* = 4.0 Hz, =CH–*C*H=C–CH=CH), 114.2 (s, Ar), 113.8 (s, Ar), 98.2 (d, *J* = 16.1 Hz, Ar), 89.3 (d, *J* = 14.3 Hz, =*C*H–CH=C–CH=CH), 80.0 (dd, *J* = 15.9, 1.8 Hz, =CH–CH=C–*C*H=CH or =CH–CH=C–CH=*C*H), 70.9 (d, *J* = 5.1 Hz, =CH–CH=C–*C*H=CH or =CH–CH=C–CH=*C*H), 55.0 (s, O*Me*), 54.7 (s, O*Me*). Some aromatic signals are obscured by the residual solvent peak.

IR (cm^–1^): 3051, 2956, 2925, 2853, 1603, 1507, 1480, 1462, 1244, 1174, 1092, 1066, 1034, 823, 742, 695, 517.

Elemental analysis: C_56_H_46_NiO_2_P_2_ calcd: C, 77.17%; H, 5.32%. Found: C, 76.75%; H, 5.45%.

### Complex **9**

The same procedure as for complex **7** was followed using 30 mg of complex **6** (0.041 mmol) and 10 μL of 1-ethynyl4-fluorobenzene (0.09 mmol). The product was obtained as a red-brown powder (27 mg, 75%).

^1^H NMR (400 MHz, C_6_D_6_, 25 °C): δ(ppm) 7.72–7.62 (m, 3H, Ar–*H*), 7.50 (dd, *J* = 7.6, 3.1 Hz, 1H, Ar–*H*), 7.48–7.44 (m, 1H, Ar–*H*), 7.31 (td, *J* = 8.0, 1.6 Hz, 2H, Ar–*H*), 7.22 (ddd, *J* = 9.8, 7.5, 1.9 Hz, 2H, Ar–*H*), 7.13–7.07 (m, 4H, Ar–*H*), 7.04 (t, *J* = 7.5 Hz, 2H, Ar–*H*), 6.92 (tdd, *J* = 11.5, 5.8, 3.2 Hz, 6H, Ar–*H*), 6.87–6.79 (m, 4H, Ar–*H*), 6.75 (td, *J* = 8.9, 2.4 Hz, 4H, Ar–*H*), 6.69 (d, *J* = 8.7 Hz, 1H, Ar–*H*), 6.67–6.61 (m, 1H, Ar–*H*), 6.47 (td, *J* = 7.7, 1.8 Hz, 2H, Ar–*H*), 5.53 (d, *J*_H–P_ = 7.3 Hz, 1H, =(CH_3_)C–C*H*=C–CH=CH), 4.18–4.10 (m, 1H, =(CH_3_)C–CH=C–C*H*=CH or =(CH_3_)C–CH=C–CH=C*H*), 3.78 (td, *J*_H–H_ = 11.3, *J*_H–P_ = 2.8 Hz, 1H, =(CH_3_)C–CH=C–C*H*=CH or =(CH_3_)C–CH=C–CH=C*H*), 1.34 (d, *J*_H–P_ = 10.7 Hz, 3H, C*H*_3_).

^31^P{^1^H} NMR (161 MHz, C_6_D_6_, 25 °C): δ(ppm) 38.9 (d, *J* = 8.6 Hz, 1P), 27.6 (d, *J* = 8.4 Hz, 1P).

^19^F NMR (376 MHz, C_6_D_6_, 25 °C): δ(ppm) −115.45 (tt, *J* = 8.8, 5.5 Hz, 1F), −121.07 (dd, *J* = 7.9, 6.1 Hz, 1F).

IR (cm^–1^): 3052, 2962, 2925, 2852, 1598, 1581, 1502, 1482, 1454, 1223, 1158, 1065, 906, 827, 745, 695, 517.

The poor solubility of this compound in common solvents did not allow us to obtain ^13^C NMR data.

The high sensitivity of this compound did not allow us to obtain elemental analysis data.

### Complex **10**

The compound was generated in situ by adding 1.2 equiv of 1-ethynyl-4-fluorobenzene (0.016 mmol, 1.8 μL) to a solution of 10 mg of complex **5** (0.014 mmol) in approximately 0.6 mL of C_6_D_6_. The solution was immediately transferred to a J-Young NMR tube, and NMR data was recorded approximately 5 min after mixing. A small amount of complex **7** is also observed.

^1^H NMR (400 MHz, C_6_D_6_, 25 °C): δ(ppm) 8.14 (q, *J* = 6.6 Hz, 3H, Ar–*H*), 7.76–7.70 (m, 3H, Ar–*H*), 7.70–7.64 (m, 2H, Ar–*H*), 7.35 (dd, *J* = 7.7, 3.9 Hz, 2H, Ar–*H*), 7.10 (d, *J* = 7.2 Hz, 8H, Ar–*H*), 7.02–6.91 (m, 8H, Ar–*H*), 6.88 (t, *J* = 7.3 Hz, 2H, Ar–*H*), 6.61 (t, *J* = 8.7 Hz, 2H, Ar–*H*), 2.08 (t, *J*_H–P_ = 2.1 Hz, 3H, Me).

^31^P{^1^H} NMR (161 MHz, C_6_D_6_, 25 °C): δ(ppm) 38.3 (s, 2P).

^19^F NMR (376 MHz, C_6_D_6_, 25 °C): δ(ppm) −117.14 (ddd, *J* = 14.4, 9.0, 5.6 Hz, 1F).

Full ^13^C NMR and IR data were not obtained because of the instability of the compound.

### Complex **11**

The same procedure as that for complex 10 was followed using 1.2 equiv of 1-ethynyl-4-fluorobenzene (0.016 mmol, 1.8 μL) to a solution of 10 mg of complex **6** (0.013 mmol) in approximately 0.6 mL of C_6_D_6_. Full conversion is observed.

^1^H NMR (400 MHz, C_6_D_6_, 25 °C): δ(ppm) 8.21 (q, *J* = 6.0 Hz, 3H, Ar–*H*), 7.65 (q, *J* = 6.2 Hz, 3H, Ar–*H*), 7.56 (d, *J* = 8.0 Hz, 2H, Ar–*H*), 7.36 (dt, *J* = 8.3, 4.3 Hz, 2H, Ar–*H*), 7.13–7.07 (m, 3H, Ar–*H*), 7.06–6.83 (m, 15H, Ar–*H*), 6.61 (t, *J* = 8.8 Hz, 2H, Ar–*H*), 2.36 (q, *J* = 7.8 Hz, 2H, C*H*_2_), 1.41 (t, *J* = 7.4 Hz, 3H, Me). Some signals are obscured by the residual solvent peak.

^31^P{^1^H} NMR (161 MHz, C_6_D_6_, 25 °C): δ(ppm) 38.3 (s, 2P).

^19^F NMR (376 MHz, C_6_D_6_, 25 °C): δ(ppm) −117.15 (ddd, *J* = 14.3, 9.0, 5.5 Hz, 1F).

Full ^13^C NMR and IR data were not obtained because of the instability of the compound.

## Supporting Information Available

NMR spectra and supplementary experimental and computational data (PDF)
Cartesian coordinates of optimized structures (XYZ)

## Supplementary Material

om3c00404_si_001.pdf

om3c00404_si_002.xyz

## Accession Codes

CCDC 2294468 contains the supplementary crystallographic data for this paper. These data can be obtained free of charge via www.ccdc.cam.ac.uk/data_request/cif, or by emailing data_request@ccdc.cam.ac.uk, or by contacting The Cambridge Crystallographic Data Centre, 12 Union Road, Cambridge CB2 1EZ, UK; fax: +44 1223 336033.

## References

[ref1] Elsby M. R.; Baker R. T. Strategies and Mechanisms of Metal-Ligand Cooperativity in First-Row Transition Metal Complex Catalysts. Chem. Soc. Rev. 2020, 49 (24), 8933–8987. 10.1039/D0CS00509F. 33164012

[ref2] Gandeepan P.; Müller T.; Zell D.; Cera G.; Warratz S.; Ackermann L. 3d Transition Metals for C–H Activation. Chem. Rev. 2019, 119 (4), 2192–2452. 10.1021/acs.chemrev.8b00507. 30480438

[ref3] Tiddens M. R.; Moret M.-E. Metal-Ligand Cooperation at Phosphine-Based Acceptor Pincer Ligands. In Metal-Ligand Co-operativity; van Koten G., Kirchner K., Moret M.-E., Eds.; Springer, 2020, p 68. 10.1007/3418_2020_70.

[ref4] Khusnutdinova J. R.; Milstein D. Metal-Ligand Cooperation. Angew. Chem., Int. Ed. 2015, 54 (42), 12236–12273. 10.1002/anie.201503873. 26436516

[ref5] Van Der Vlugt J. I. Cooperative Catalysis with First-Row Late Transition Metals. Eur. J. Inorg. Chem. 2012, 2012 (3), 363–375. 10.1002/ejic.201100752.

[ref6] Harman W. H.; Peters J. C. Reversible H 2 Addition across a Nickel-Borane Unit as a Promising Strategy for Catalysis. J. Am. Chem. Soc. 2012, 134 (11), 5080–5082. 10.1021/ja211419t. 22380492

[ref7] Harman W. H.; Lin T. P.; Peters J. C. A d^10^ Ni–(H_2_) Adduct as an Intermediate in H-H Oxidative Addition across a Ni-B Bond. Angew. Chem., Int. Ed. 2014, 53 (4), 1081–1086. 10.1002/anie.201308175. 24323761

[ref8] MacMillan S. N.; Hill Harman W.; Peters J. C. Facile Si-H Bond Activation and Hydrosilylation Catalysis Mediated by a Nickel-Borane Complex. Chem. Sci. 2014, 5 (2), 590–597. 10.1039/c3sc52626g.

[ref9] Verhoeven D. G. A.; Moret M. E. Metal-Ligand Cooperation at Tethered π-Ligands. Dalton Trans. 2016, 45 (40), 15762–15778. 10.1039/C6DT02184K. 27460960

[ref10] Chase P. A.; Gossage R. A.; Van Koten G. Modern Organometallic Multidentate Ligand Design Strategies: The Birth of the Privileged “Pincer” Ligand Platform. In The Privileged Pincer-Metal Platform: Coordination Chemistry & Applications; Springer, 2015. 10.1007/3418_2015_131.

[ref11] Manar K. K.; Ren P. Recent Progress on Group 10 Metal Complexes of Pincer Ligands: From Synthesis to Activities and Catalysis. In Advances in Organometallic Chemistry, 1st ed.; Elsevier Inc., 2021; Vol. 76. 10.1016/bs.adomc.2021.04.003.

[ref12] Alig L.; Fritz M.; Schneider S. First-Row Transition Metal (De)Hydrogenation Catalysis Based on Functional Pincer Ligands. Chem. Rev. 2019, 119 (4), 2681–2751. 10.1021/acs.chemrev.8b00555. 30596420

[ref13] Verhoeven D. G. A.; Negenman H. A.; Orsino A. F.; Lutz M.; Moret M. E. Versatile Coordination and C-C Coupling of Diphosphine-Tethered Imine Ligands with Ni(II) and Ni(0). Inorg. Chem. 2018, 57 (17), 10846–10856. 10.1021/acs.inorgchem.8b01478. 30113165 PMC6150681

[ref14] Orsino A. F.; Moret M. E. Nickel-Catalyzed Alkyne Cyclotrimerization Assisted by a Hemilabile Acceptor Ligand: A Computational Study. Organometallics 2020, 39 (10), 1998–2010. 10.1021/acs.organomet.0c00172.

[ref15] Verhoeven D. G. A.; van Wiggen M. A. C.; Kwakernaak J.; Lutz M.; Klein Gebbink R. J. M.; Moret M. E. Periodic Trends in the Binding of a Phosphine-Tethered Ketone Ligand to Fe, Co, Ni, and Cu. Chem.—Eur. J. 2018, 24 (20), 5163–5172. 10.1002/chem.201703254. 29077236

[ref16] Verhoeven D. G. A.; Kwakernaak J.; van Wiggen M. A. C.; Lutz M.; Moret M. E. Cobalt(II) and (I) Complexes of Diphosphine-Ketone Ligands: Catalytic Activity in Hydrosilylation Reactions. Eur. J. Inorg. Chem. 2019, 2019 (5), 660–667. 10.1002/ejic.201801221. 31007578 PMC6472597

[ref17] Barrett B. J.; Iluc V. M. Coordination of a Hemilabile Pincer Ligand with an Olefinic Backbone to Mid-to-Late Transition Metals. Inorg. Chem. 2014, 53 (14), 7248–7259. 10.1021/ic500549z. 24959947

[ref18] Barrett B. J.; Iluc V. M. Group 10 Metal Complexes Supported by Pincer Ligands with an Olefinic Backbone. Organometallics 2014, 33 (10), 2565–2574. 10.1021/om500256r.

[ref19] Verhoeven D. G. A.; Orsino A. F.; Bienenmann R. L. M.; Lutz M.; Moret M. E. Cooperative Si-H Addition to Side-On Ni(0)-Imine Complexes Forms Reactive Hydrosilazane Complexes. Organometallics 2020, 39 (4), 623–629. 10.1021/acs.organomet.0c00059.

[ref20] Orsino A. F.; Gutiérrez del Campo M.; Lutz M.; Moret M. E. Enhanced Catalytic Activity of Nickel Complexes of an Adaptive Diphosphine-Benzophenone Ligand in Alkyne Cyclotrimerization. ACS Catal. 2019, 9 (3), 2458–2481. 10.1021/acscatal.8b05025. 30854242 PMC6400243

[ref21] Sansores-Paredes M. L. G.; van der Voort S.; Lutz M.; Moret M. Divergent Reactivity of an Isolable Nickelacyclobutane. Angew. Chem., Int. Ed. 2021, 60 (51), 26518–26522. 10.1002/anie.202111389. PMC9298726 34608737

[ref22] Saes B. W. H.; Verhoeven D. G. A.; Lutz M.; Klein Gebbink R. J. M.; Moret M. E. Coordination of a Diphosphine-Ketone Ligand to Ni(0), Ni(I), and Ni(II): Reduction-Induced Coordination. Organometallics 2015, 34 (12), 2710–2713. 10.1021/acs.organomet.5b00264.

[ref23] Polukeev A. V.; Wendt O. F. Iridium Pincer Complexes with an Olefin Backbone. Organometallics 2015, 34 (17), 4262–4271. 10.1021/acs.organomet.5b00495.

[ref24] Sung S.; Tinnermann H.; Krämer T.; Young R. D. Direct Oxide Transfer from an Η2-Keto Ligand to Generate a Cobalt PCcarbeneP(O) Pincer Complex. Dalton Trans. 2019, 48 (27), 9920–9924. 10.1039/C9DT02313E. 31184666

[ref25] Vigalok A.; Kraatz H.; Konstantinovsky L.; Milstein D. Evidence for Direct Trans Insertion in a Hydrido-Olefin Rhodium Complex—Free Nitrogen as a Trap in a Migratory Insertion Process. Chem.—Eur. J. 1997, 3 (2), 253–260. 10.1002/chem.19970030214. 24022956

[ref26] Barrett B. J.; Iluc V. M. An Adaptable Chelating Diphosphine Ligand for the Stabilization of Palladium and Platinum Carbenes. Organometallics 2017, 36 (3), 730–741. 10.1021/acs.organomet.6b00924.

[ref27] Polukeev A. V.; Marcos R.; Ahlquist M. S. G.; Wendt O. F. Formation of a C-C Double Bond from Two Aliphatic Carbons. Multiple C-H Activations in an Iridium Pincer Complex. Chem. Sci. 2015, 6 (3), 2060–2067. 10.1039/C4SC03839H. 28717458 PMC5496475

[ref28] Sansores-Paredes M. L. G.; Lutz M.; Moret M.-E. Cooperative H_2_ Activation at a Nickel(0)-Olefin Center Nat. Chem. 2023 10.1038/s41557-023-01380-1 38052947

[ref29] Ramani A.; Desai B.; Patel M.; Naveen T. Recent Advances in the Functionalization of Terminal and Internal Alkynes. Asian J. Org. Chem. 2022, 11, e202200047 10.1002/ajoc.202200047.

[ref30] He B.; Huang J.; Liu X.; Zhang J.; Lam J. W. Y.; Tang B. Z. Polymerizations of Activated Alkynes. Prog. Polym. Sci. 2022, 126, 101503 10.1016/j.progpolymsci.2022.101503.

[ref31] Guihaumé J.; Halbert S.; Eisenstein O.; Perutz R. N. Hydrofluoroarylation of Alkynes with Ni Catalysts. C-H Activation via Ligand-to-Ligand Hydrogen Transfer, an Alternative to Oxidative Addition. Organometallics 2012, 31 (4), 1300–1314. 10.1021/om2005673.

[ref32] Perutz R. N.; Sabo-Etienne S. The σ-CAM Mechanism: σ Complexes as the Basis of σ-Bond Metathesis at Late-Transition-Metal Centers. Angew. Chem., Int. Ed. 2007, 46 (15), 2578–2592. 10.1002/anie.200603224. 17380532

[ref33] Tang S.; Eisenstein O.; Nakao Y.; Sakaki S. Aromatic C-H σ-Bond Activation by Ni0, Pd0, and Pt0 Alkene Complexes: Concerted Oxidative Addition to Metal vs Ligand-to-Ligand H Transfer Mechanism. Organometallics 2017, 36 (15), 2761–2771. 10.1021/acs.organomet.7b00256.

[ref34] CCDC 2294468 contains the supplementary crystallographic data for this paper. These data can be obtained free of charge from The Cambridge Crystallographic Data Centre via www.ccdc.cam.ac.uk/data_request/cif.

[ref35] Ryu H.; Park J.; Kim H. K.; Park J. Y.; Kim S. T.; Baik M. H. Pitfalls in Computational Modeling of Chemical Reactions and How to Avoid Them. Organometallics 2018, 37 (19), 3228–3239. 10.1021/acs.organomet.8b00456.

[ref36] Huang X.; Zhu J.; Lin Z. β-Hydrogen Elimination of Five-Membered-Ring Metallacycles. Is It Possible?. Organometallics 2004, 23 (17), 4154–4159. 10.1021/om049570o.

[ref37] Vivancos Á.; Hernández Y. A.; Paneque M.; Poveda M. L.; Salazar V.; Álvarez E. Formation of β-Metallanaphthalenes by the Coupling of a Benzo-Iridacyclopentadiene with Olefins. Organometallics 2015, 34 (1), 177–188. 10.1021/om5010435.

[ref38] Campora J.; Llebaria A.; Moreto J. M.; Poveda M. L.; Carmona E. Reactions of the Benzonickelacyclopentene Complex [Cyclic] (Me3P)2Ni(CH2CMe2-o-C6H4) with Alkynes. Synthesis of 1,2-Dihydronaphthalenes. Organometallics 1993, 12 (10), 4032–4038. 10.1021/om00034a040.

[ref39] Qiu Z.; Xie Z. Nickel-Catalyzed Three-Component [2 + 2 + 2] Cycloaddition Reaction of Arynes, Alkenes, and Alkynes. Angew. Chem., Int. Ed. 2009, 48 (31), 5729–5732. 10.1002/anie.200902006. 19557782

[ref40] Paneque M.; Posadas C. M.; Poveda M. L.; Rendón N.; Álvarez E.; Mereiter K. Investigations on the Coupling of Ethylene and Alkynes in [IrTp] Compounds: Water as an Effective Trapping Agent. Chem.—Eur. J. 2007, 13 (18), 5160–5172. 10.1002/chem.200601500. 17377937

[ref41] Vivancos Á.; Rendón N.; Paneque M.; Poveda M. L.; Álvarez E. Reactivity of a Tp-Iridacyclopentene Complex. Organometallics 2015, 34 (22), 5438–5453. 10.1021/acs.organomet.5b00775.

[ref42] Paneque M.; Poveda M. L.; Rendón N.; Álvarez E.; Carmona E. The Synthesis of Iridabenzenes by the Coupling of Iridacyclopentadienes and Olefins. Eur. J. Inorg. Chem. 2007, 2007 (18), 2711–2720. 10.1002/ejic.200601257.

[ref43] Bennett M. A.; Hockless D. C. R.; Wenger E. Generation of (2,3-.eta.)-Naphthalyne-Nickel(0) Complexes and Their Reactions with Unsaturated Molecules. Organometallics 1995, 14 (4), 2091–2101. 10.1021/om00004a071.

[ref44] Bennett M. A.; Glewis M.; Hockless D. C. R.; Wenger E. Successive Insertion of Tetrafluoroethylene and CO and of Tetrafluoroethylene and Acetylenes into Aryne-Nickel(0) Bonds. J. Chem. Soc., Dalton Trans. 1997, 3105–3114. 10.1039/a702375h.

[ref45] Saraev V. V.; Kraikivskii P. B.; Vilms A. I.; Zelinskii S. N.; Yunda A. Y.; Danilovtseva E. N.; Kuzakov A. S. Cyclotrimerization and Linear Oligomerization of Phenylacetylene on the Nickel(I) Monocyclopentadienyl Complex CpNi(PPh3)2. Kinet. Catal. 2007, 48 (6), 778–784. 10.1134/S002315840706002X.

[ref46] Chen M.; Montgomery J. Nickel-Catalyzed Intermolecular Enantioselective Heteroaromatic C-H Alkylation. ACS Catal. 2022, 12, 11015–11023. 10.1021/acscatal.2c03228.

[ref47] Zhan X.; Xu S.; Yang M.; Lei Z. Chloro-Nickel and Chloro-Cobalt Complexes Containing Phosphine Ligands: Efficient Initiators for Polymerization of Alkynes. Catal. Lett. 2002, 80 (1/2), 59–61. 10.1023/A:1015374610059.

[ref48] Zhan X.; Yang M. Polymerization of P-Diethynylbenzene and Its Derivatives with Nickelocene Acetylide Catalysts Containing Different Phosphine and Alkynyl Ligands. Macromol. Rapid Commun. 2000, 21 (17), 1263–1266. 10.1002/1521-3927(20001101)21:17<1263::aid-marc1263>3.0.co;2-x.

[ref49] Li Y.; Yang M. Transition Metal Acetylide Catalysts for Polymerization of Alkynes. J. Mol. Catal. A: Chem. 2002, 184 (1–2), 161–165. 10.1016/S1381-1169(02)00058-4.

[ref50] Frisch M. J.; Trucks G. W.; Schlegel H. B.; Scuseria G. E.; Robb M. a.; Cheeseman J. R.; Scalmani G.; Barone V.; Petersson G. a.; Nakatsuji H.; G16_C01, p Gaussian 16, Revision C.01; Gaussian, Inc.: Wallin, 2016.

