# Supplementary material for: Reactions of Nickel(0)–Olefin Pincer Complexes with Terminal Alkynes: Cooperative C–H Bond Activation and Alkyne Coupling
Source: Organometallics. 2023 Nov 22;42(23):3418–27. doi: 10.1021/acs.organomet.3c00404 (PMC10716905; doi:10.1021/acs.organomet.3c00404)
Supplement: Supplementary file 1 — om3c00404_si_001.pdf [file om3c00404_si_001.pdf]

Supporting information for:

Reactions of nickel(0) olefin pincer complexes with terminal alkynes: cooperative C–H bond activation and alkyne coupling.

María L. G. Sansores-Paredes[1], Tú T. T. Nguyen[1], Martin Lutz[2] and Marc-Etienne Moret[1]*

[1]Organic Chemistry & Catalysis, Institute for Sustainable and Circular Chemistry, Utrecht University, 3584 CG Utrecht, The Netherlands.
Email corresponding author: M.moret@uu.nl
[2]Structural Biochemistry, Bijvoet Centre for Biomolecular Research, Utrecht University, 3584 CG Utrecht, The Netherlands.

Content

1. Additional experiments *1*

1.1 Experiment with 1.2 equivalent of 1-ethynyl4-fluorobenzene and complex 5 1

1.2 Experiment 1.2 equivalent of 1-ethynyl-4-fluorobenzene and complex 6 10

2. Spectra of new compounds *17*

3. X-ray crystal structure determination of complex 8 .... *55*

4. Computational studies *56*

4.1 Additional comments about C–H activation mechanism 56

4.2 Additional comments about the alkene/alkyne coupling 57

4.3 Comparison nickelacyclopentadiene intermediate 59

4.4 Table of energies 61

5. References *62*

1. Additional experiments

1.1 Experiment with 1.2 equivalent of 1-ethynyl4-fluorobenzene and complex 5

Adding a limited amount of 1-ethynyl-4-fluorobenzene to complex **5** allows the observation C–H bond activation to form product **10** and its reversibility (Scheme S1). 10 min after adding 1-ethynl-4-fluorobenzene to a C_6D_6 solution of complex **5**, full conversion to complex **10** is observed. Over time, formation of complex **7** and regeneration of **5** are observed, showing the reversibility in the C–H activation process. The ^{1}H NMR spectra of the process are depicted in Figure S1 and Figure S2. The process can also be followed by ^{31}P (Figure S3 and Figure S4) and ^{19}F NMR (Figure S5 and Figure S6). An additional nickelacyclopentene intermediate **12** is observed, further details are discussed below.

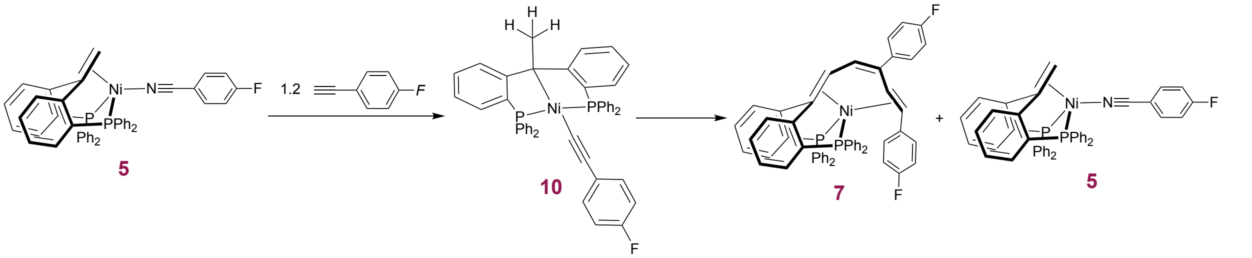

Scheme S1. Reaction of complex 3 with 1.2 equivalents of 1-ethynyl-4-fluorobenzene.

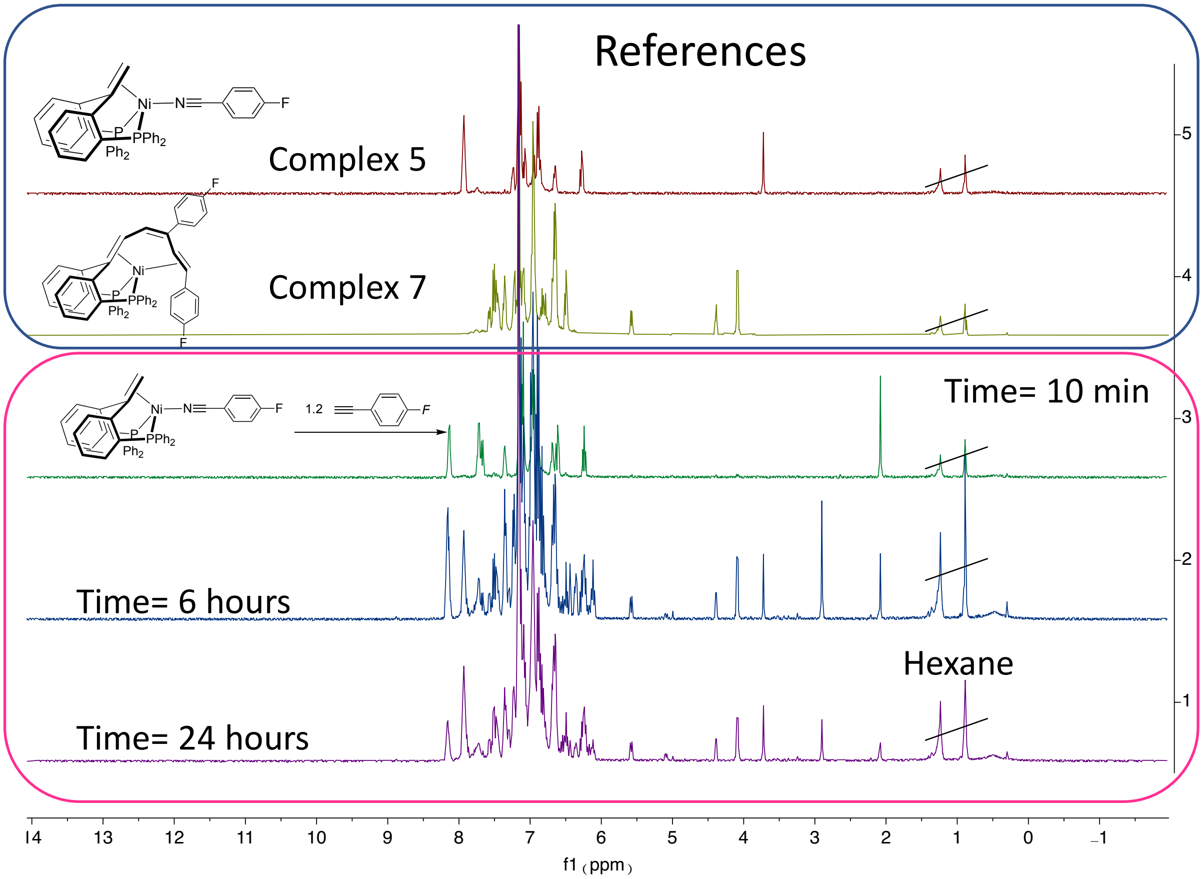

Figure S1. Full ^{1}H NMR spectra of the reaction of complex **5** with 1.2 equivalents of 1-ethynyl-4-fluorobenzene in C_6D_6. On top inside the blue rectangle: references of complexes **5** and **7**. Inside the pink rectangle: reaction monitoring at different times.

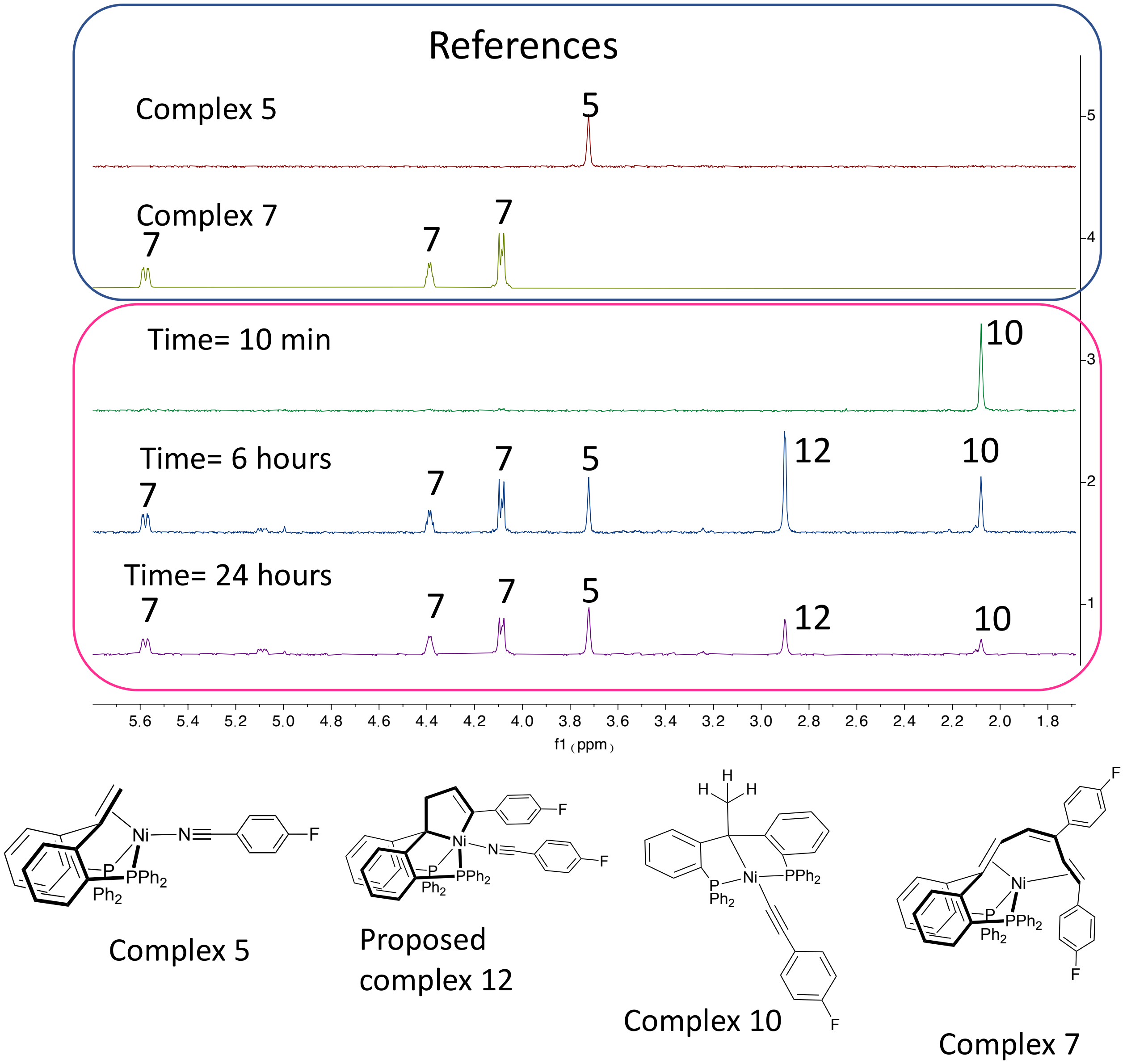

Figure S2. ¹H NMR spectra of the of reaction of complex **5** with 1.2 equivalents of 1-ethynyl-4-fluorobenzene in the range 1.8-6 ppm region in C_6D_6. On top inside the blue rectangle: references of complex **5** and **7**. Inside the pink rectangle: reaction monitoring at different times.

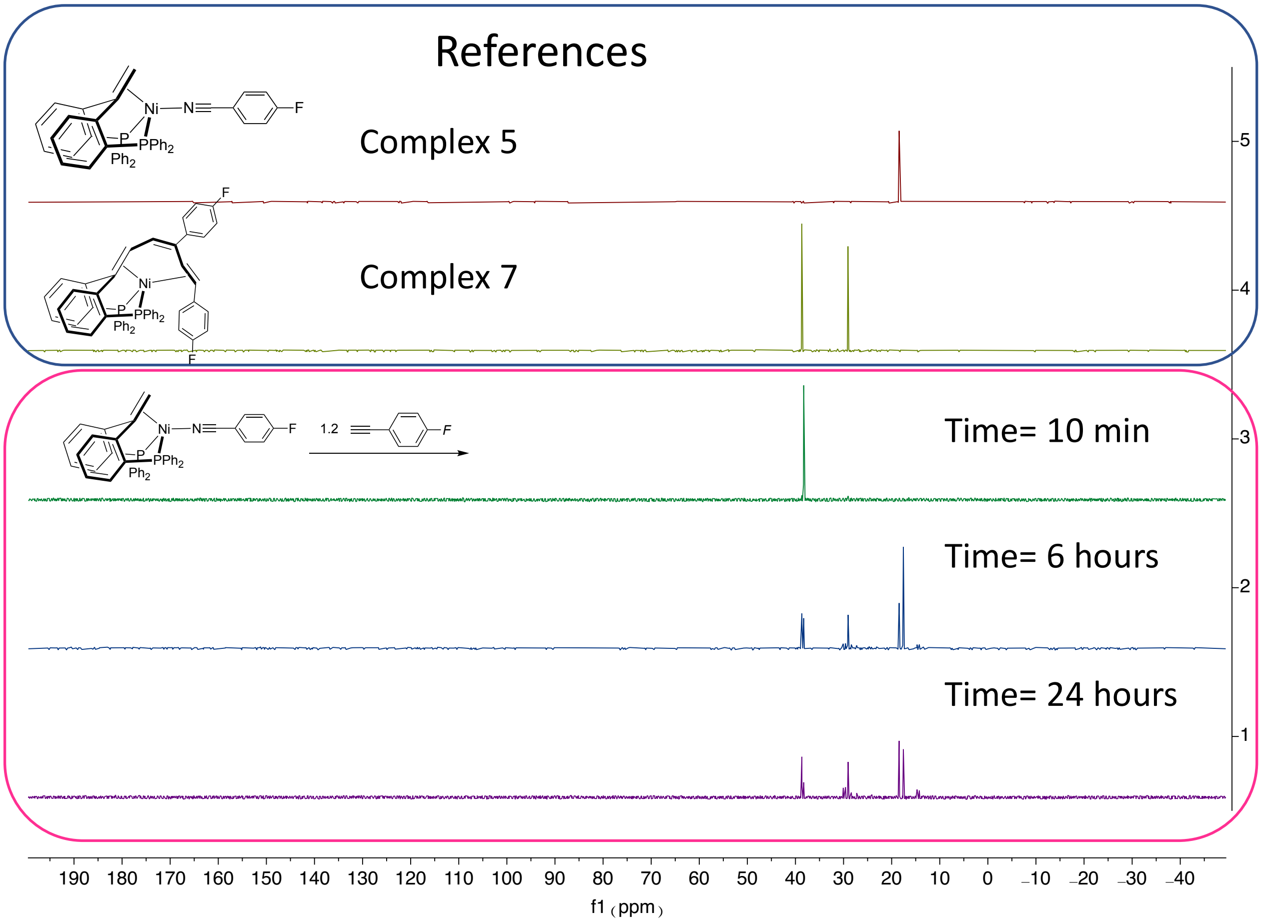

Figure S3. Full $^{31}P\{^{1}H\}$ NMR spectra of the reaction of complex **5** with 1.2 equivalents of 1-ethynyl-4-fluorobenzene in C_6D_6. On top inside the blue rectangle: references of complex **5** and **7**. Inside the pink square: reaction follow-up at different times.

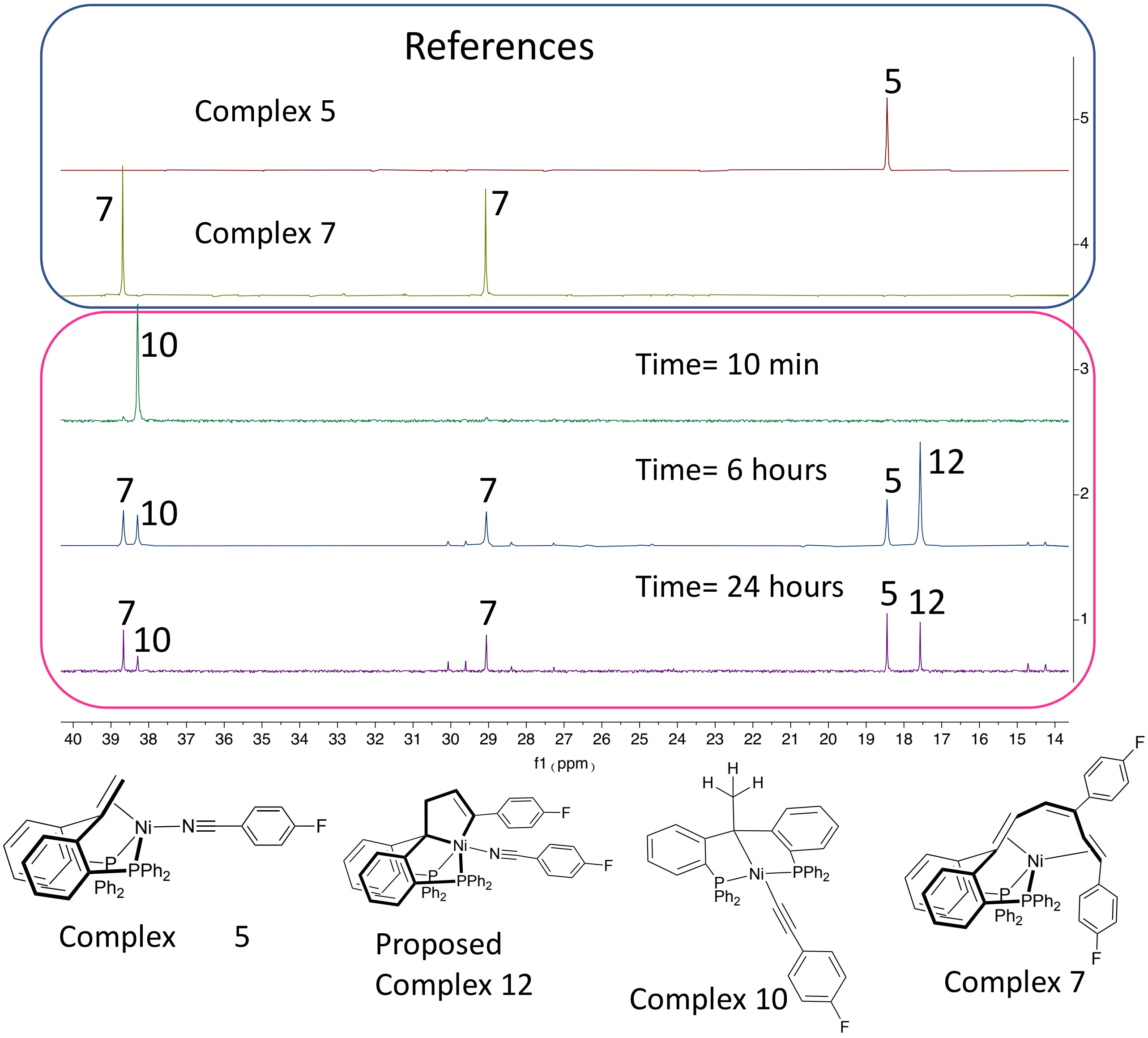

Figure S4. $^{31}P\{^{1}H\}$ NMR spectra of the reaction of complex **5** with 1.2 equivalents of 1-ethynyl-4-fluorobenzene in C_6D_6 in the 14-40 ppm region. On top inside the blue rectangle: references of complex **5** and **7**. Inside the pink rectangle: reaction monitoring at different times.

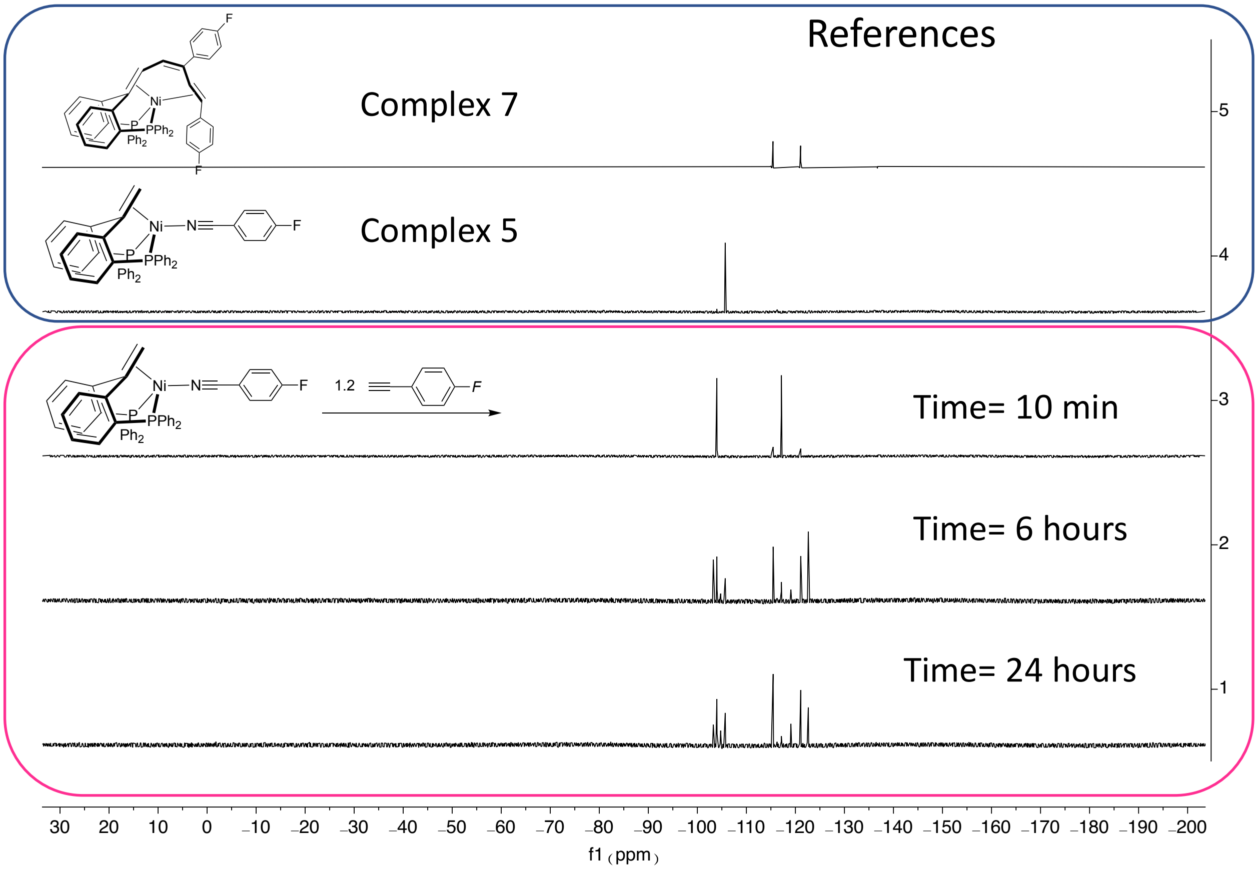

Figure S5. Full ^{19}F NMR spectra of the reaction of complex **5** with 1.2 equivalents of 1-ethynyl-4-fluorobenzene in C_6D_6. On top inside the blue rectangle: references of complex **5** and **7**. Inside the pink square: reaction monitoring at different times.

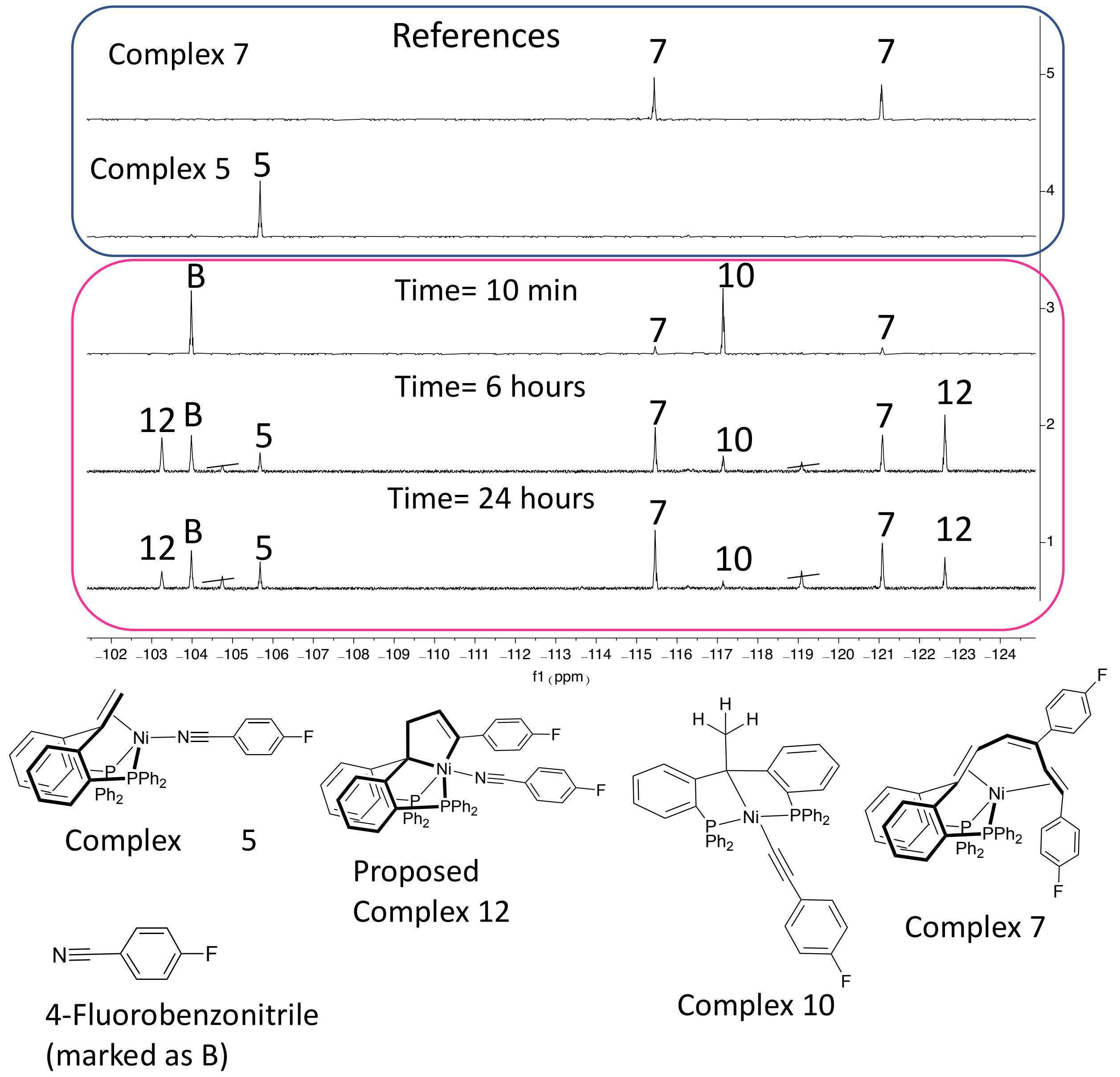

Figure S6. ^{19}F NMR spectra of the reaction of complex **5** with 1.2 equivalents of 1-ethynyl-4-fluorobenzene in C_6D_6 in the –101 - –125 ppm region. On top inside the blue rectangle: references of complex **5** and **7**. Inside the pink rectangle: reaction follow-up at different times. Crossed peaks correspond to an unidentified compound.

From the data in Figure S6, it may at first sight be surprising that complex **7** is observed without any intermediate **12** at early reaction times. This can be explained by the fact that the rate of conversion of **12** to **7** is dependent on the concentration of free alkyne. At early times, there is a significant concentration of free alkyne from the excess 0.2 equivalents, which results in intermediate **12** being consumed faster than it is formed. Under these conditions, intermediate **12** does not accumulate. When the excess is consumed, the second molecule of alkyne comes from the displacement of the alkyne ligand in the equilibrium mixture **13** <--> **10** by fluorobenzonitrile, which is an endergonic process.

Therefore, the effective concentration of alkyne is exceedingly low, slowing down the conversion of **12** to **7** to the extent that **12** accumulates and can be observed.

Figure S7 and Figure S8 contain NMR spectra relevant to the identification of intermediate **12** with relevant integrals after 6 and 24 hours, respectively. The peaks associated to intermediate **12** are found at: ^{1}H NMR δ(C_6D_6) 2.90 ppm (2H, broad doublet, CH_2); ^{31}P{^{1}H} NMR δ(C_6D_6) 17.6 ppm (2P, singlet); ^{19}F NMR δ(C_6D_6) –103.25 (1F, multiplet) and –122.6 ppm (1F, multiplet). One of the ^{19}F NMR signals is found in the area associated with 4-fluorobenzonitrile (downfield) and the other in the of 1-ethynyl-4-fluorobenzene area (upfield). An analysis of the integrals supports these peaks are associated to the same structure (Table S1and Table S2). Formation of this intermediate is also supported by DFT calculations (see section 4.2).

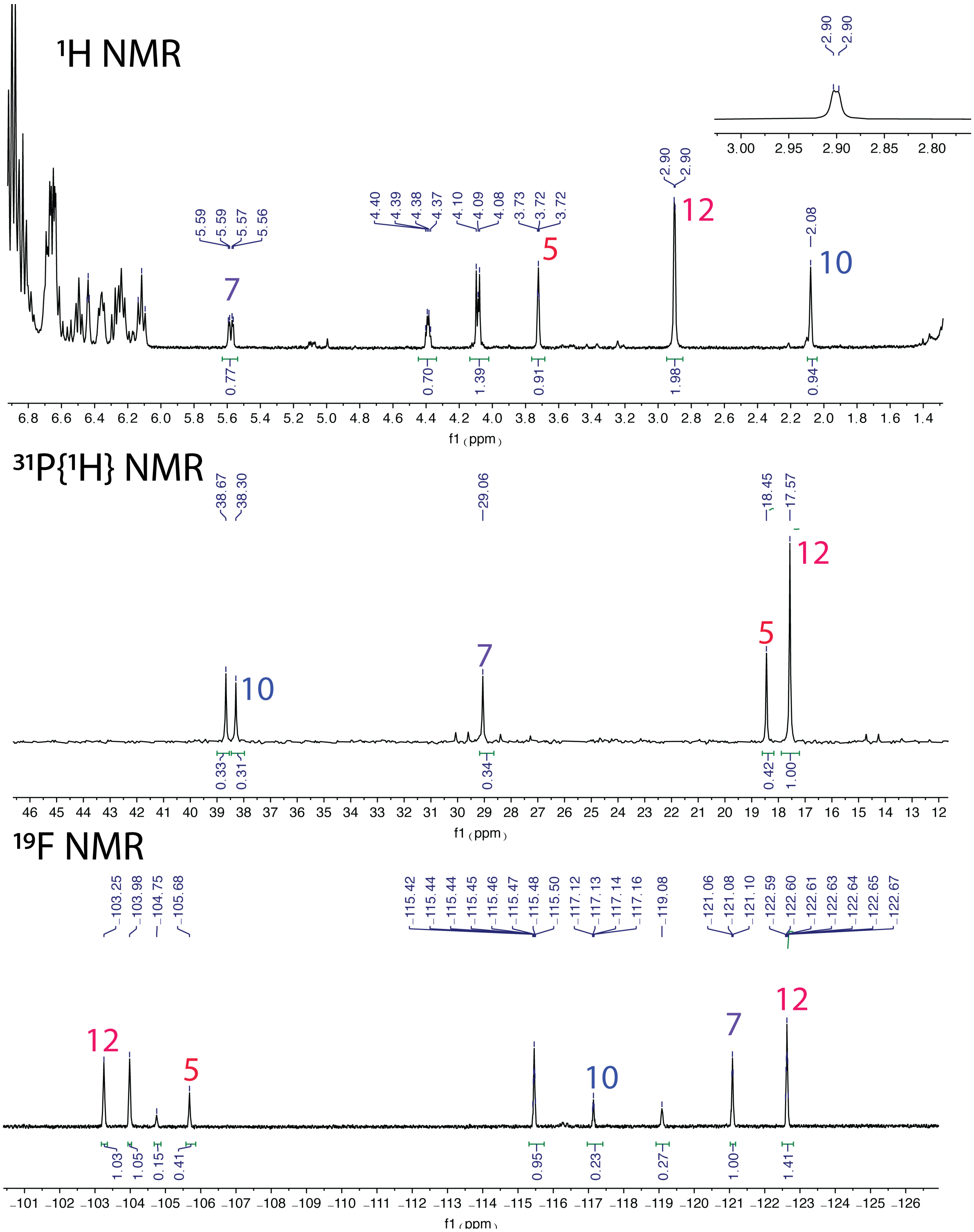

Figure S7. NMR spectra with relevant integrals of the reaction of complex **5** with 1.2 equivalents of 1-ethynyl-4-fluorobenzene in C_6D_6 after 6 hours. Species observed: complex 5, complex **10**, complex **7**, proposed intermediate **12**.

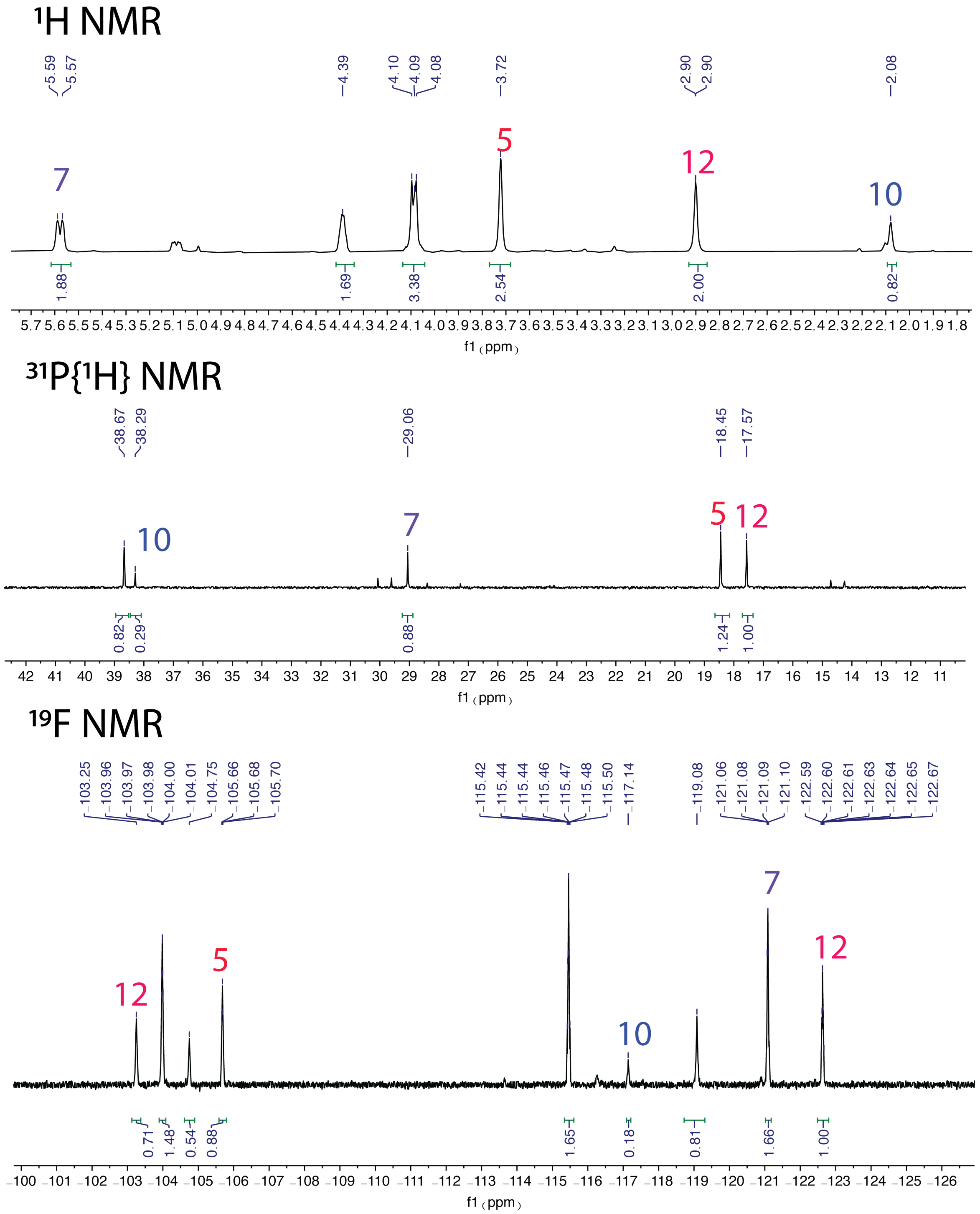

Figure S8. NMR spectra with relevant integrals of the reaction of complex **5** with 1.2 equivalents of 1-ethynyl-4-fluorobenzene in C_6D_6 after 24 hours. Species observed: complex 5, complex **10**, complex **7**, proposed intermediate **12**.

Table S1: Integral and percentage of observed intermediates after 6 hours.

Compound	Integral ^{1}H NMR (%)	Integral ^{31}P NMR (%)	Integral ^{19}F NMR (%)
Intermediate 12[a]	1.98 (39)	1 (41)	1.03 (39)
Complex 7[b]	0.77 (31)	0.34 (29)	1 (37)
Complex 5[c]	0.91 (18)	0.42 (17)	0.41 (15)
Complex 10[d]	0.94 (12)	0.31 (13)	0.23 (9)

[a]Selected peaks: ^{1}H NMR δ 2.90 ppm, ^{31}P NMR δ 17.6 ppm, ^{19}F NMR δ -103.25. [b]Selected peaks: ^{1}H NMR δ 5.58 ppm, ^{31}P NMR δ 29.0 ppm, ^{19}F NMR δ -121.08 ppm. [c]Selected peaks: ^{1}H NMR δ 3.72 ppm, ^{31}P NMR δ 18.5 ppm, ^{19}F NMR δ -105.68 ppm. [d]Selected peaks: ^{1}H NMR δ 2.09 ppm, ^{31}P NMR δ 38.3 ppm, ^{19}F NMR δ -117.14 ppm.

Table S2. Integral and percentage of observed intermediates after 24 hours.

Compound	Integral ^{1}H NMR (%)	Integral ^{31}P NMR (%)	Integral ^{19}F NMR (%)
Intermediate 12[a]	2 (23)	1 (23)	0.71 (21)
Complex 7[b]	1.88 (42)	0.88 (41)	1.66 (48)
Complex 5[c]	1.27 (29)	1.24 (29)	0.88 (26)
Complex 10[d]	0.27 (6)	0.29 (7)	0.18 (5)

[a]Selected peaks: ^{1}H NMR δ 2.90 ppm, ^{31}P NMR δ 17.6 ppm, ^{19}F NMR δ -103.25. [b]Selected peaks: ^{1}H NMR δ 5.58 ppm, ^{31}P NMR δ 29.0 ppm, ^{19}F NMR δ -121.08 ppm. [c]Selected peaks: ^{1}H NMR δ 3.72 ppm, ^{31}P NMR δ 18.5 ppm, ^{19}F NMR δ -105.68 ppm. [d]Selected peaks: ^{1}H NMR δ 2.09 ppm, ^{31}P NMR δ 38.3 ppm, ^{19}F NMR δ -117.14 ppm.

1.2 Experiment 1.2 equivalent of 1-ethynyl-4-fluorobenzene and complex 6

As observed in section 1.1, reversible C–H bond activation by complex **6** can be observed with a limited amount of 1-ethynyl-4-fluorobenzene. The reversible formation of complex **11** is observed (Scheme S2). 10 min after addition of 1-ethynl-4-fluorobenzene to a C_6D_6 solution of complex **6**, full conversion to complex **11** is observed. Overtime, formation of complex **9** and regeneration of **6** are observed, showing the reversibility in the C–H activation process also for this ligand framework. The ^{1}H NMR spectra of the process are depicted in Figure S9 and Figure S10. ^{31}P NMR (Figure S11 and Figure S12) and ^{19}F NMR (Figure S13 and Figure S14) are also presented.

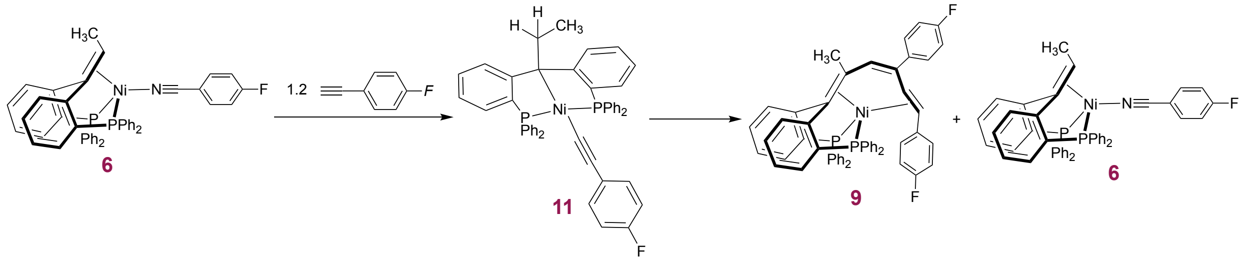

Scheme S2. Reaction of complex **6** with 1.2 equivalents of 1-ethynyl-4-fluorobenzene.

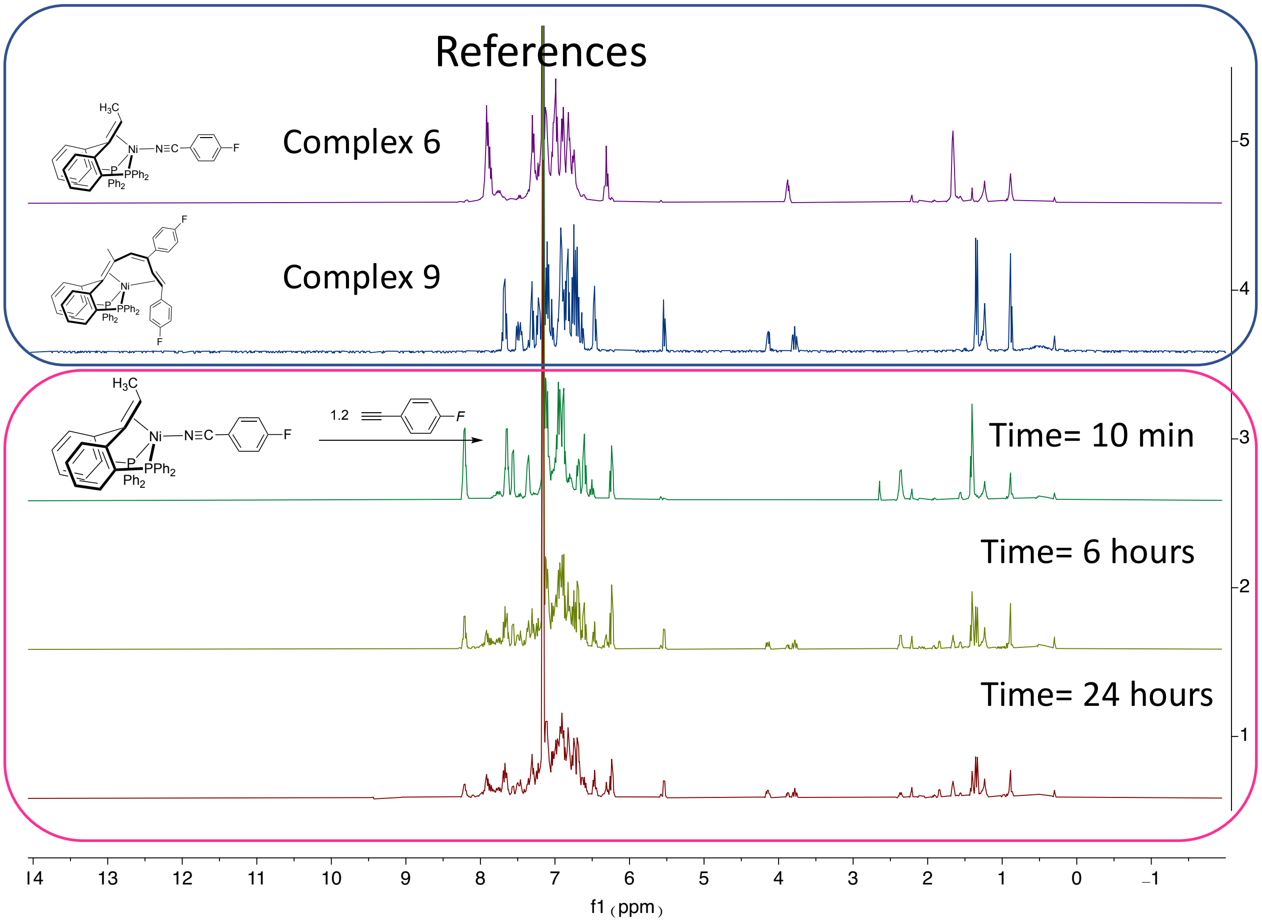

Figure S9. Full ^{1}H NMR spectra of the reaction of complex **6** with 1.2 equivalents of 1-ethynyl-4-fluorobenzene in C_6D_6. On top inside the blue rectangle: reference spectra of complex **6** and **9**. Inside the pink rectangle: reaction monitoring at different times.

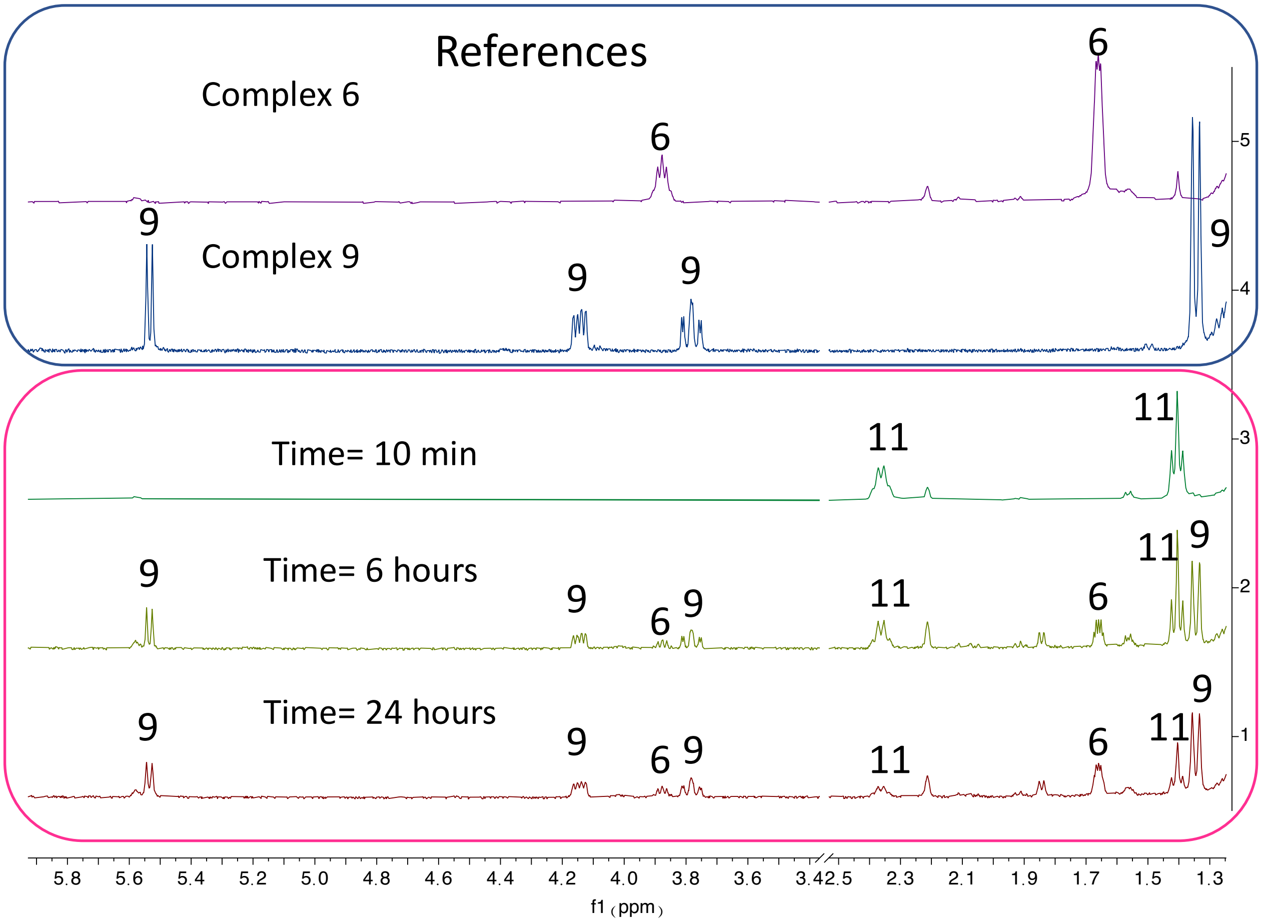

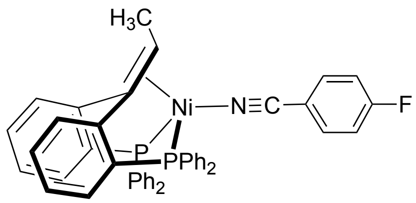

Complex 6

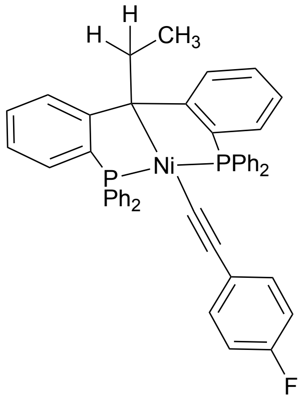

Complex 11

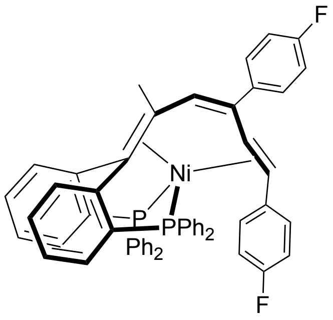

Complex 9

Figure S10. ^{1}H NMR spectra of the reaction of complex **6** with 1.2 equivalents of 1-ethynyl-4-fluorobenzene in the range 1.8-6 ppm region in C_6D_6. On top inside the blue rectangle: references of complex **6** and **9**. Inside the pink rectangle: reaction follow-up at different times.

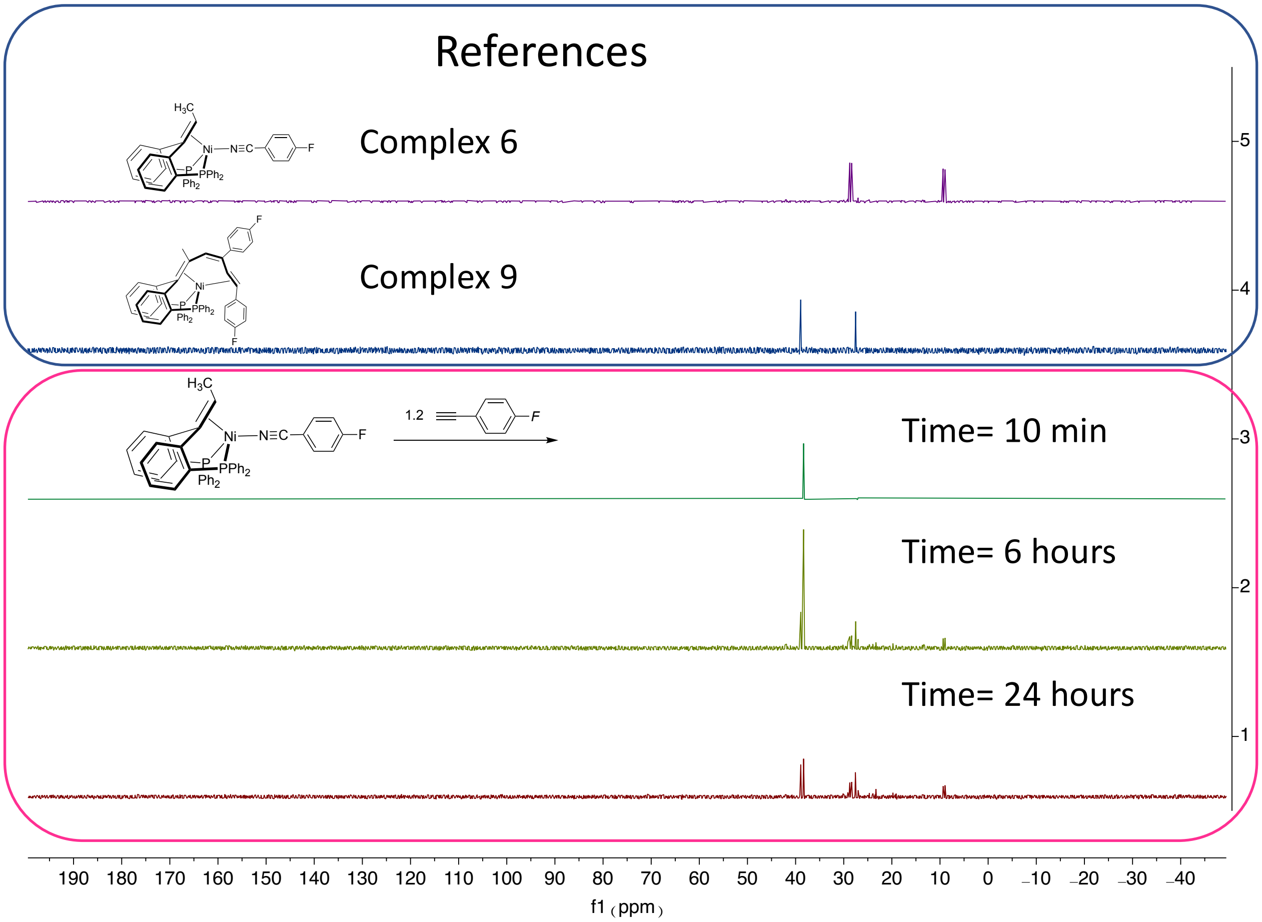

Figure S11. Full $^{31}P\{^{1}H\}$ NMR spectra of the reaction of complex **6** with 1.2 equivalents of 1-ethynyl-4-fluorobenzene in C_6D_6. On top inside the blue rectangle: references of complex **6** and **9**. Inside the pink rectangle: reaction follow-up at different times.

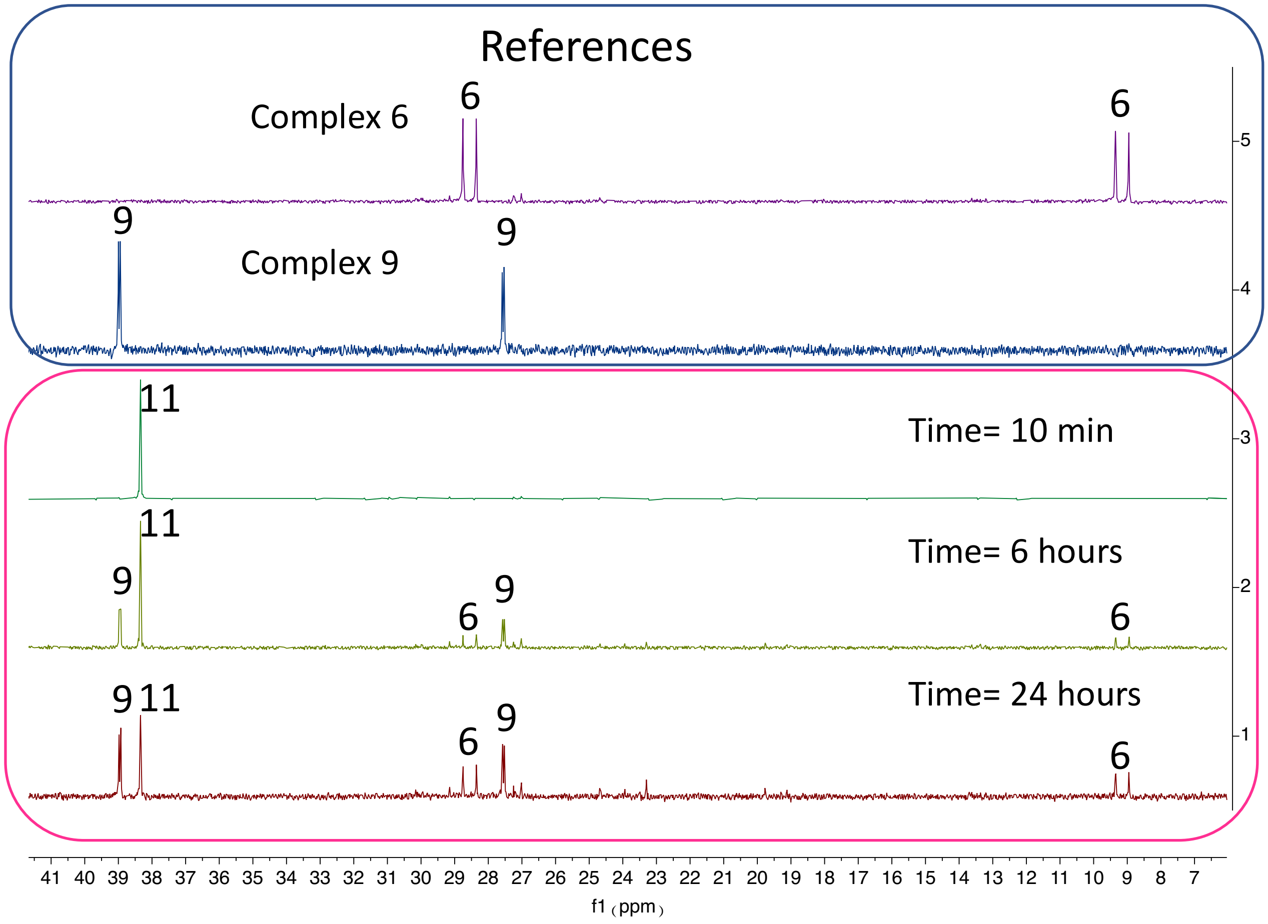

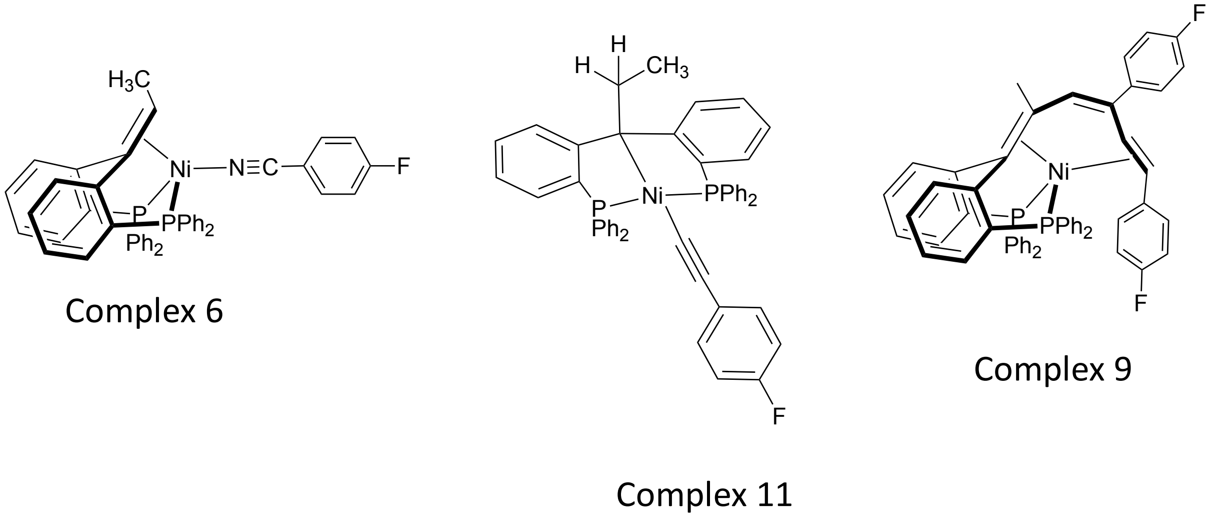

Figure S12. $^{31}P\{^1H\}$ NMR spectra of the reaction of complex **6** with 1.2 equivalents of 1-ethynyl-4-fluorobenzene in C_6D_6 in the 9-42 ppm region. On top inside the blue rectangle: references of complex **6** and **9**. Inside the pink rectangle: reaction monitoring at different times.

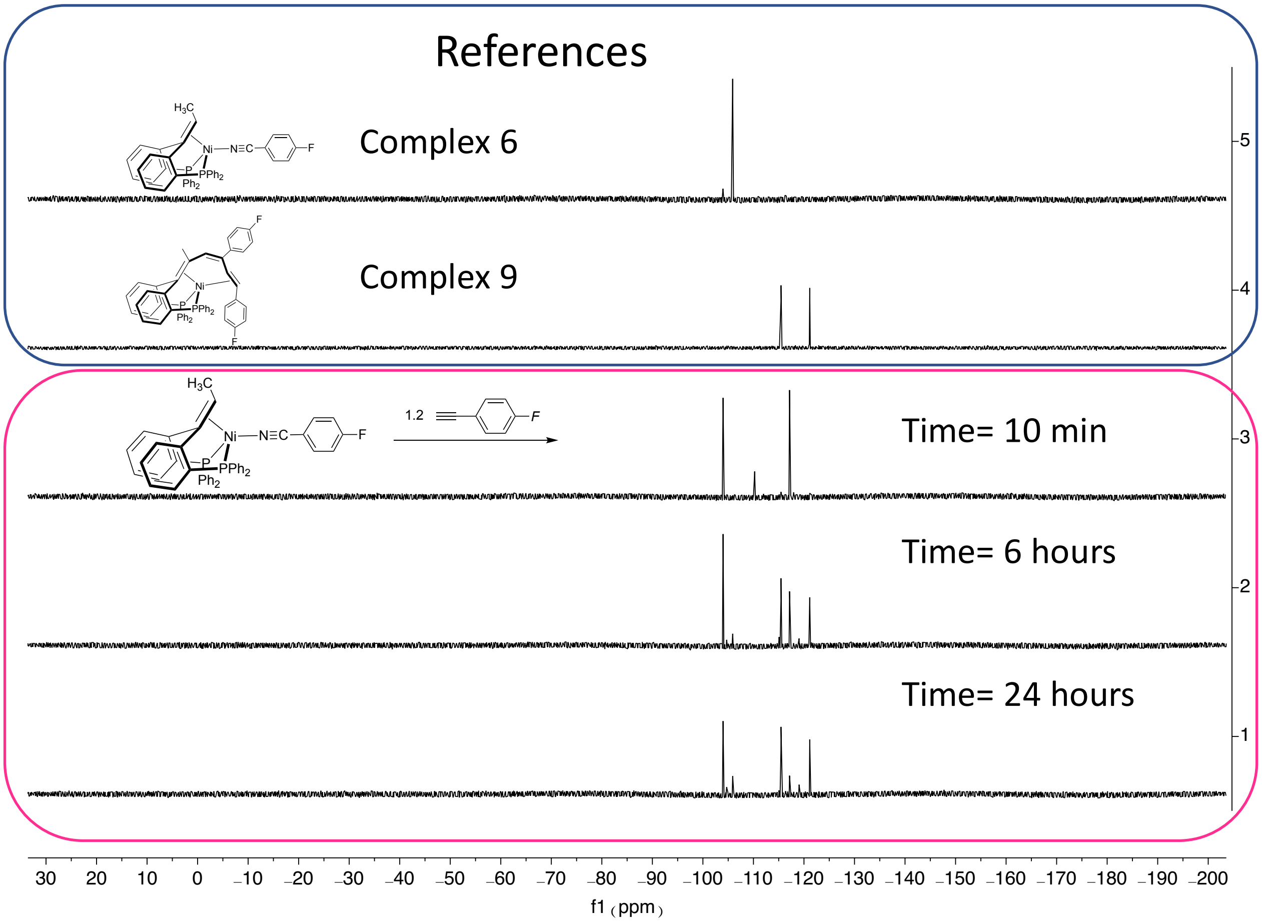

Figure S13. ^{19}F NMR spectra of the reaction of complex **6** with 1.2 equivalents of 1-ethynyl-4-fluorobenzene in C_6D_6. On top inside the blue rectangle: references of complex **6** and **9**. Inside the pink rectangle: reaction monitoring at different times.

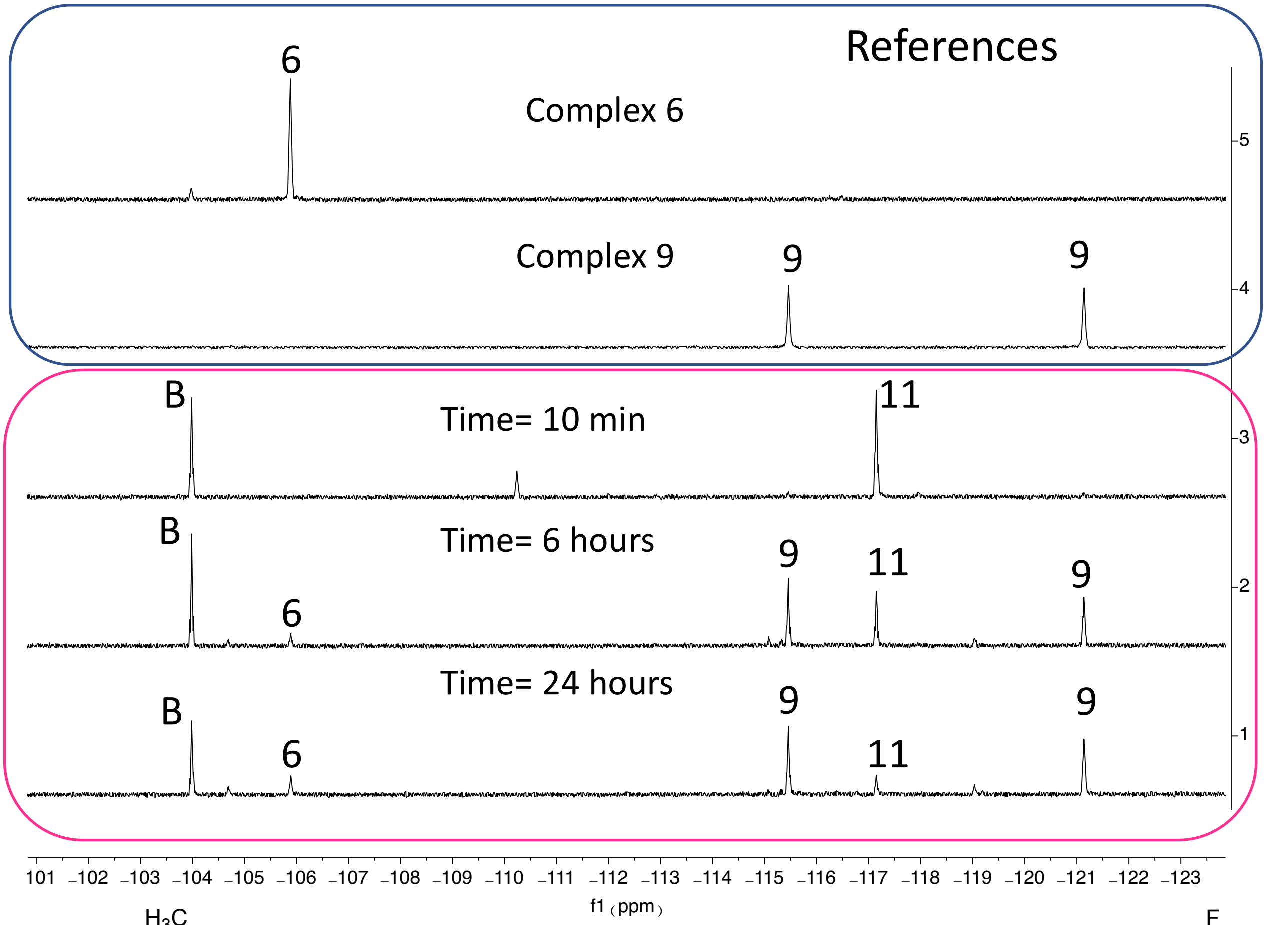

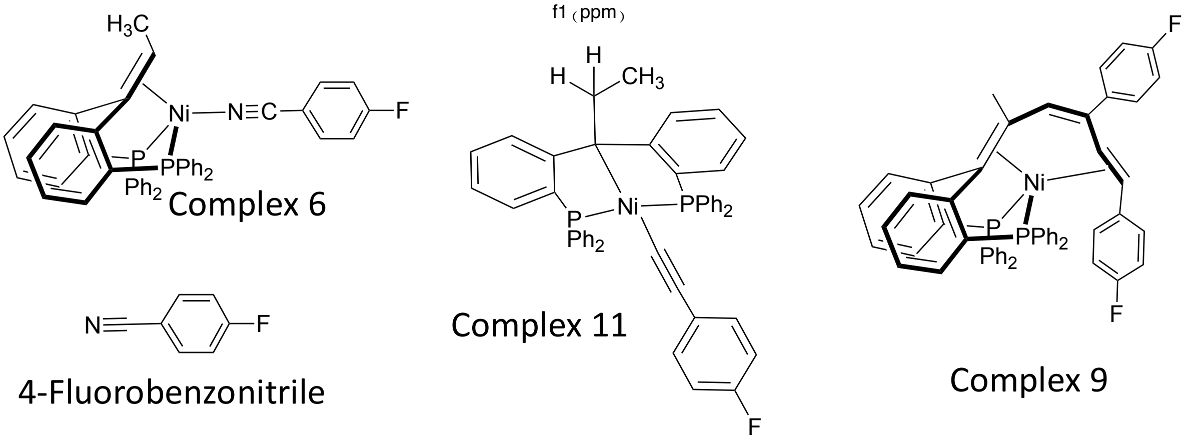

Figure S14. ^{19}F NMR spectra of the reaction of complex **6** with 1.2 equivalents of 1-ethynyl-4-fluorobenzene in C_6D_6 in the –101 - –124 ppm region. On top inside the blue rectangle: references of complex **6** and **9**. Inside the pink rectangle: reaction monitoring at different times.

2. Spectra of new compounds

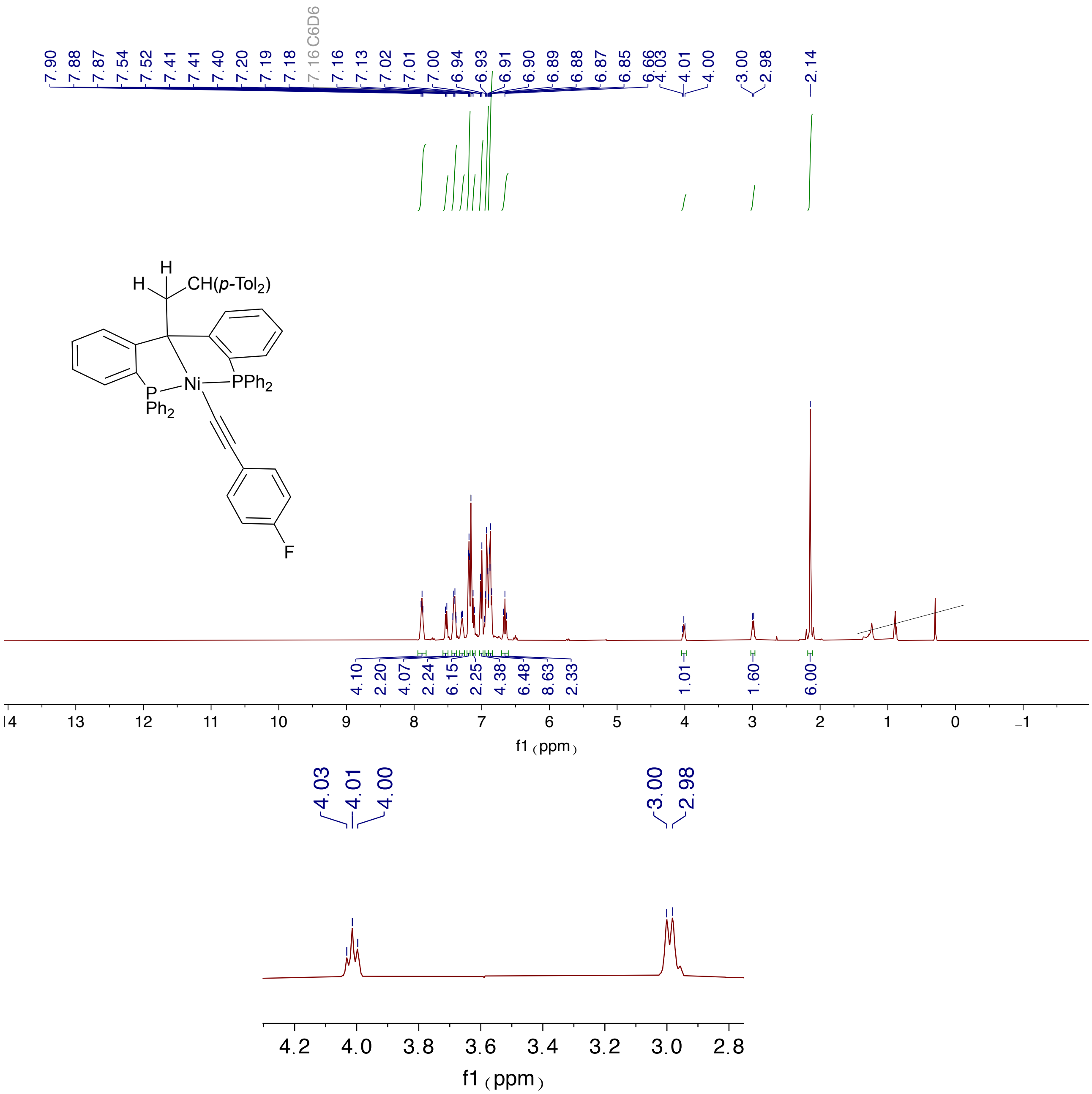

Figure S15. ^{1}H NMR spectrum of complex **2** in C_6D_6 at 25 ºC. Crossed peaks correspond to hexane and silicon grease.

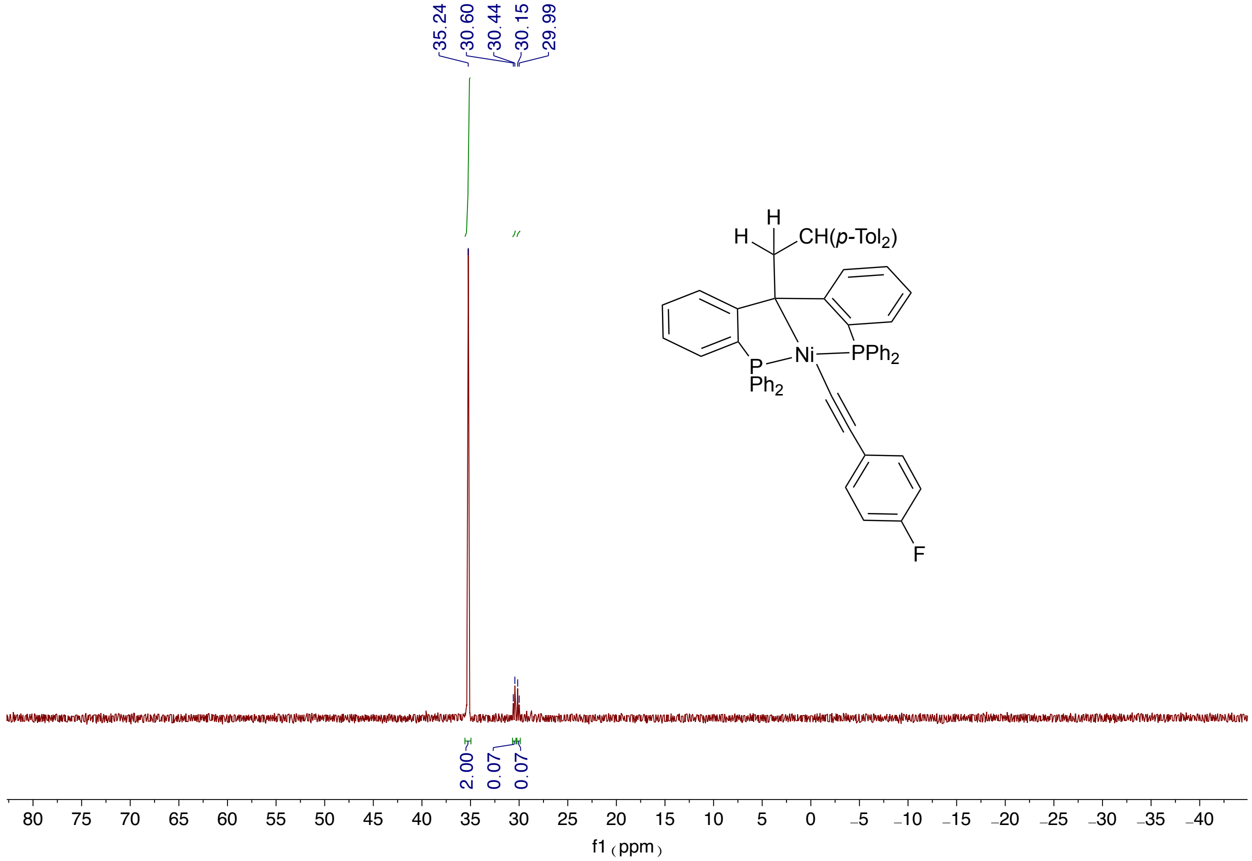

Figure S16. $^{31}P\{^{1}H\}$ NMR spectrum of complex **2** in C_6D_6 at 25 ºC.

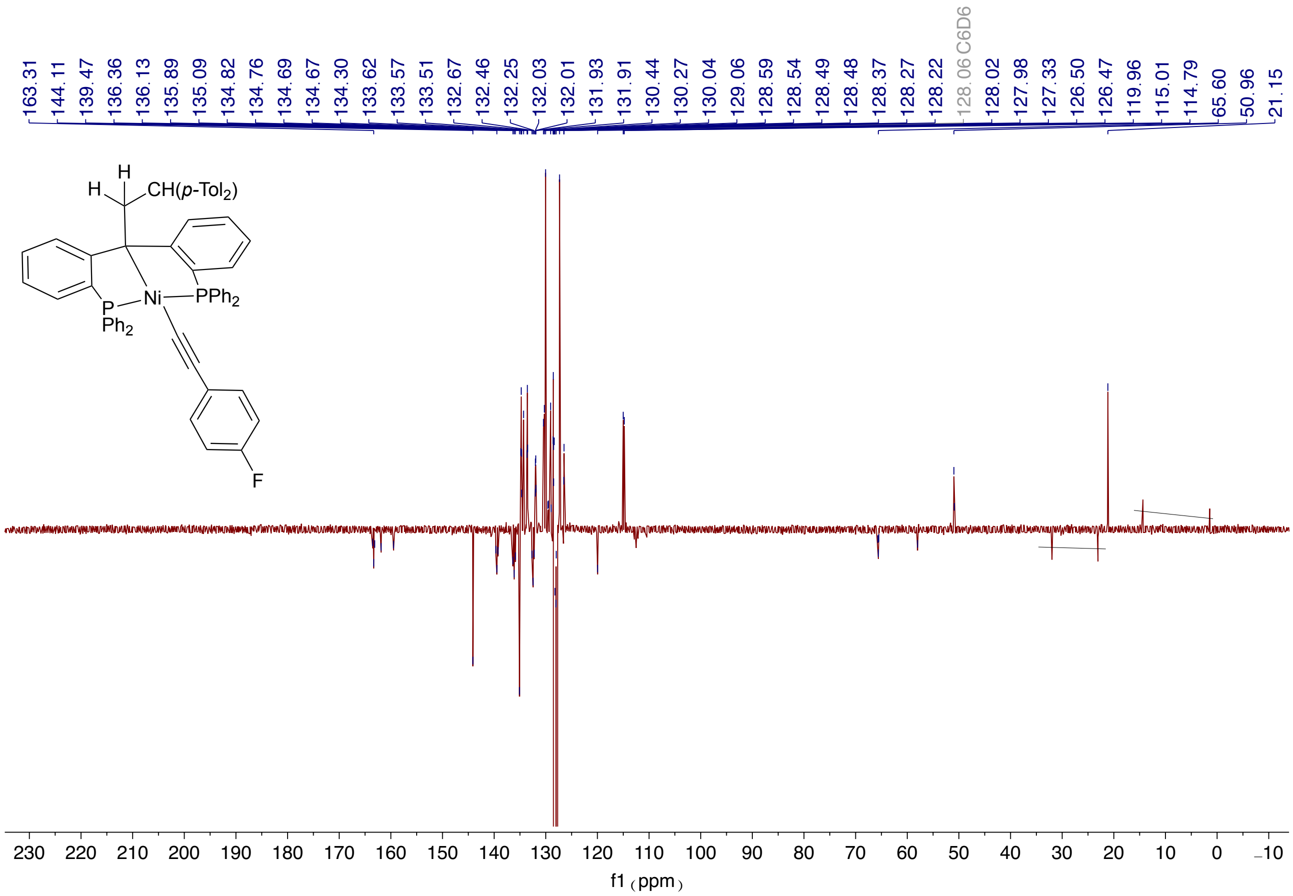

Figure S17. ^{13}C APT spectrum of complex **2** in C_6D_6 at 25 °C. Crossed peaks correspond to hexane.

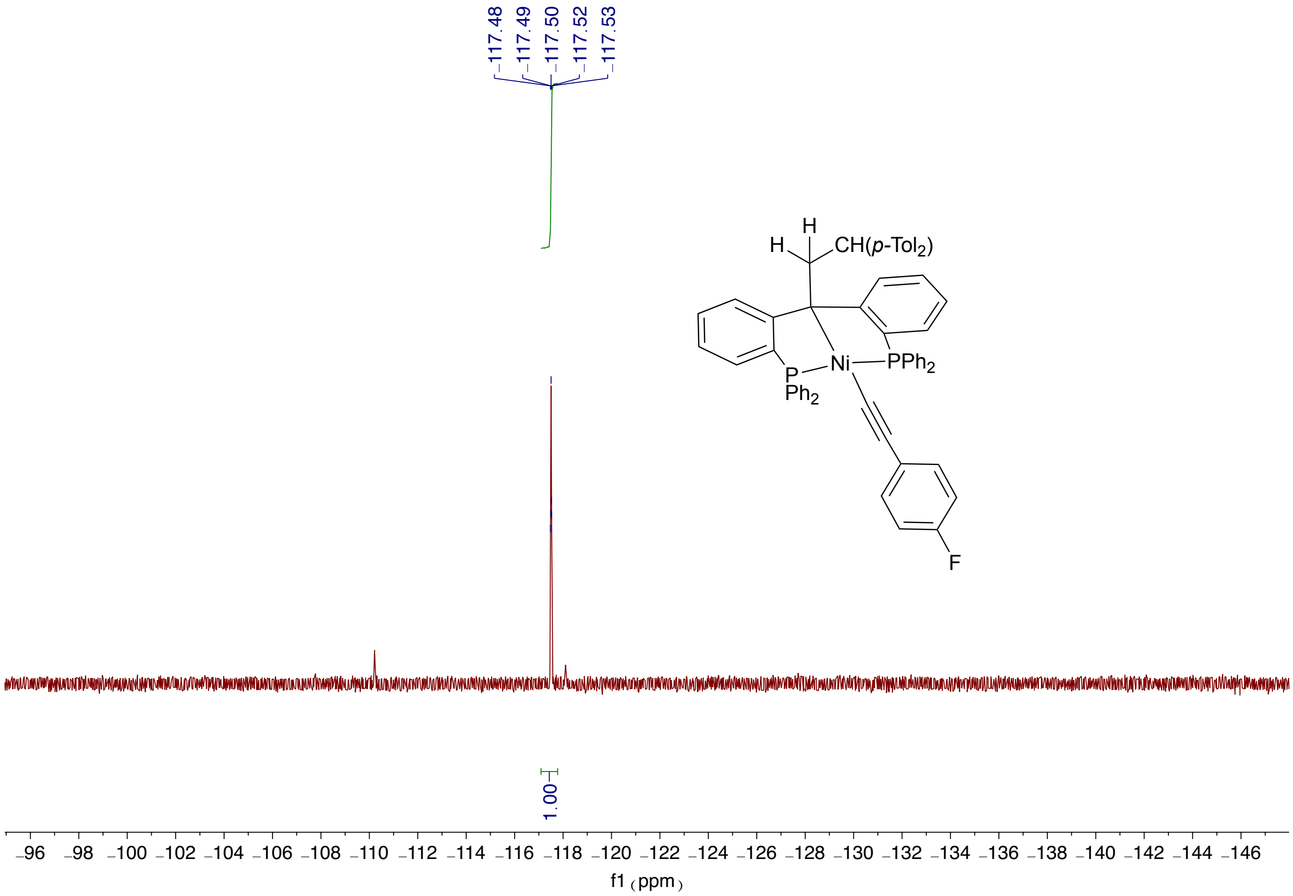

Figure S18. ^{19}F NMR spectrum of complex **2** in C_6D_6 at 25 ºC.

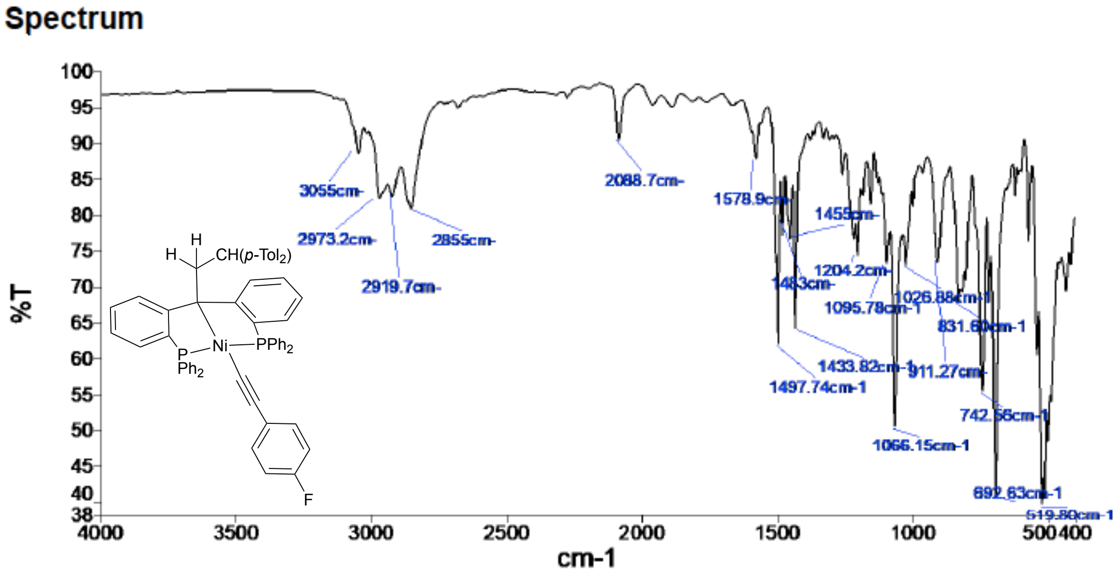

Figure S19. FTIR spectrum of complex **2** at 25 ºC.

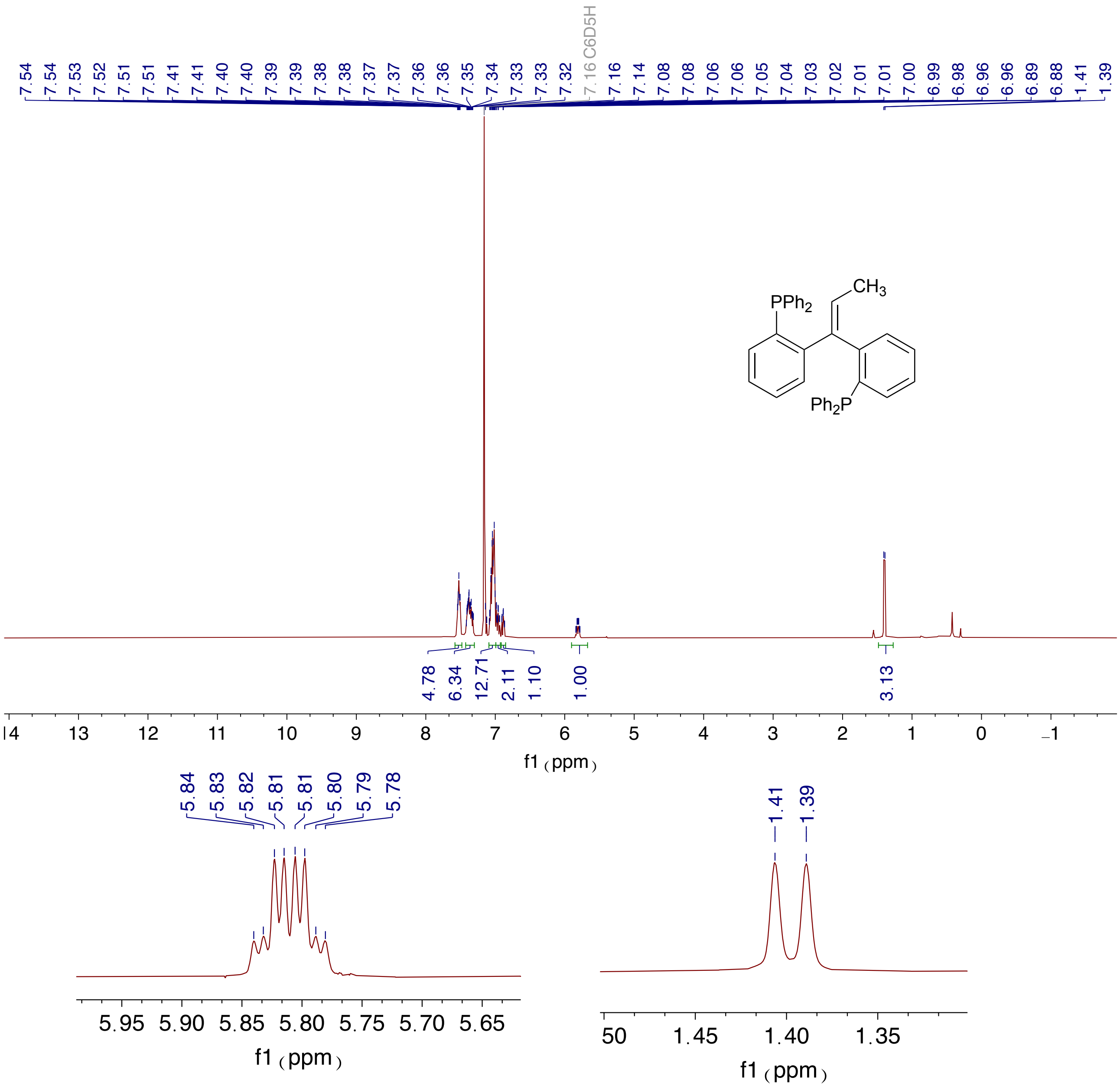

Figure S20. ^{1}H NMR spectrum of ligand **4** in C_6D_6 at 25 ºC.

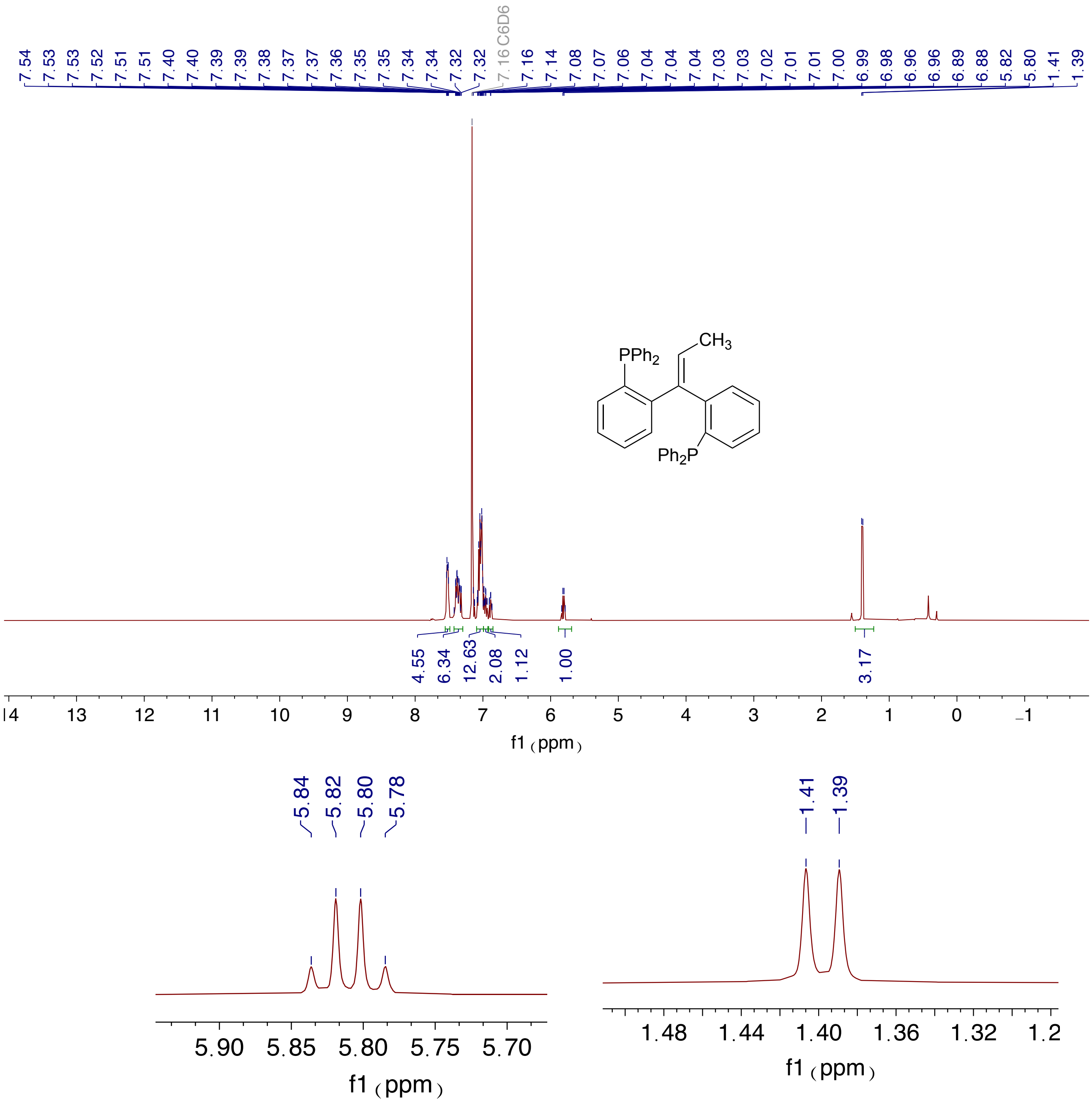

Figure S21. $^{1}H\{^{31}P\}$ NMR spectrum of ligand **4** in C_6D_6 at 25 °C.

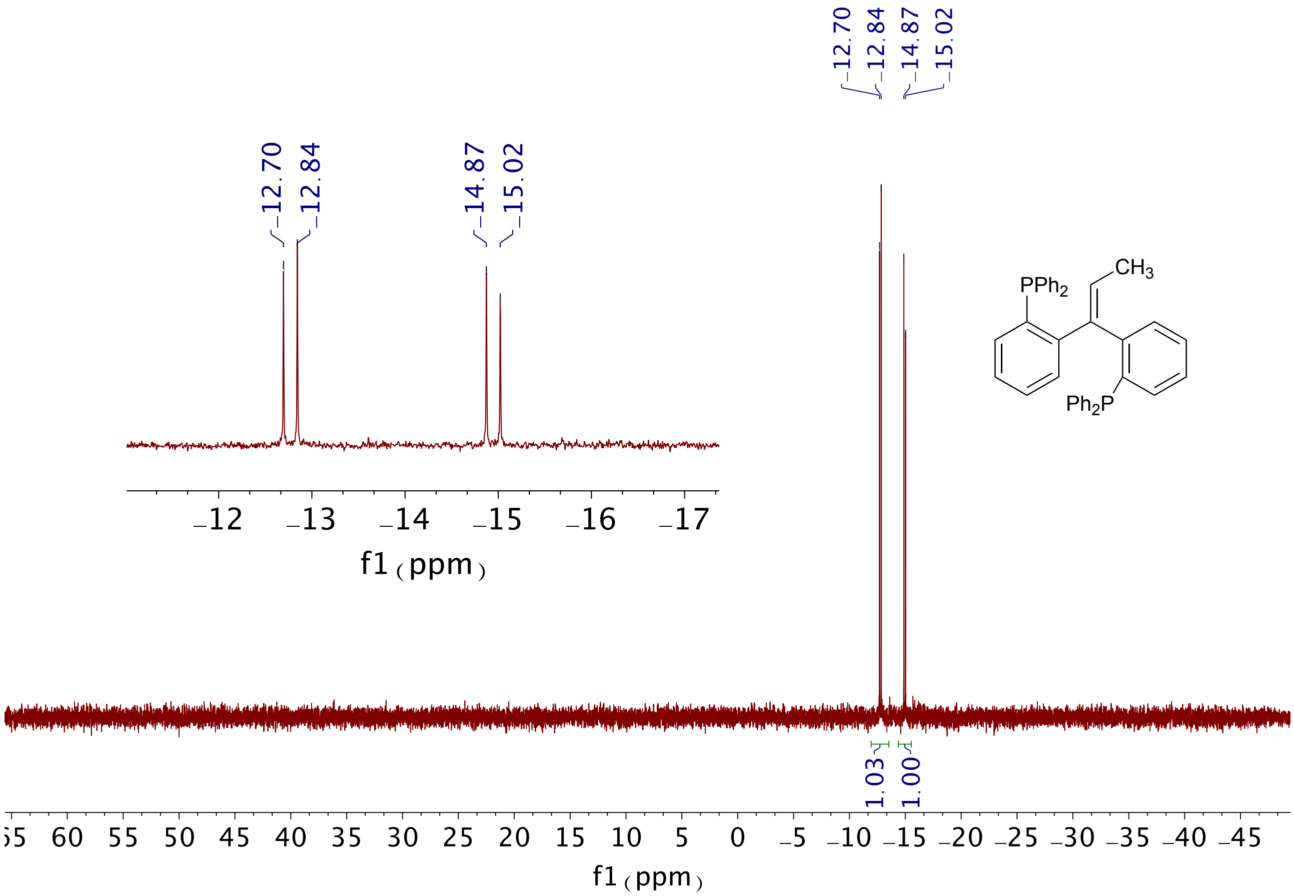

Figure S22. $^{31}P\{^{1}H\}$ NMR spectrum of ligand **4** in C_6D_6 at 25 °C.

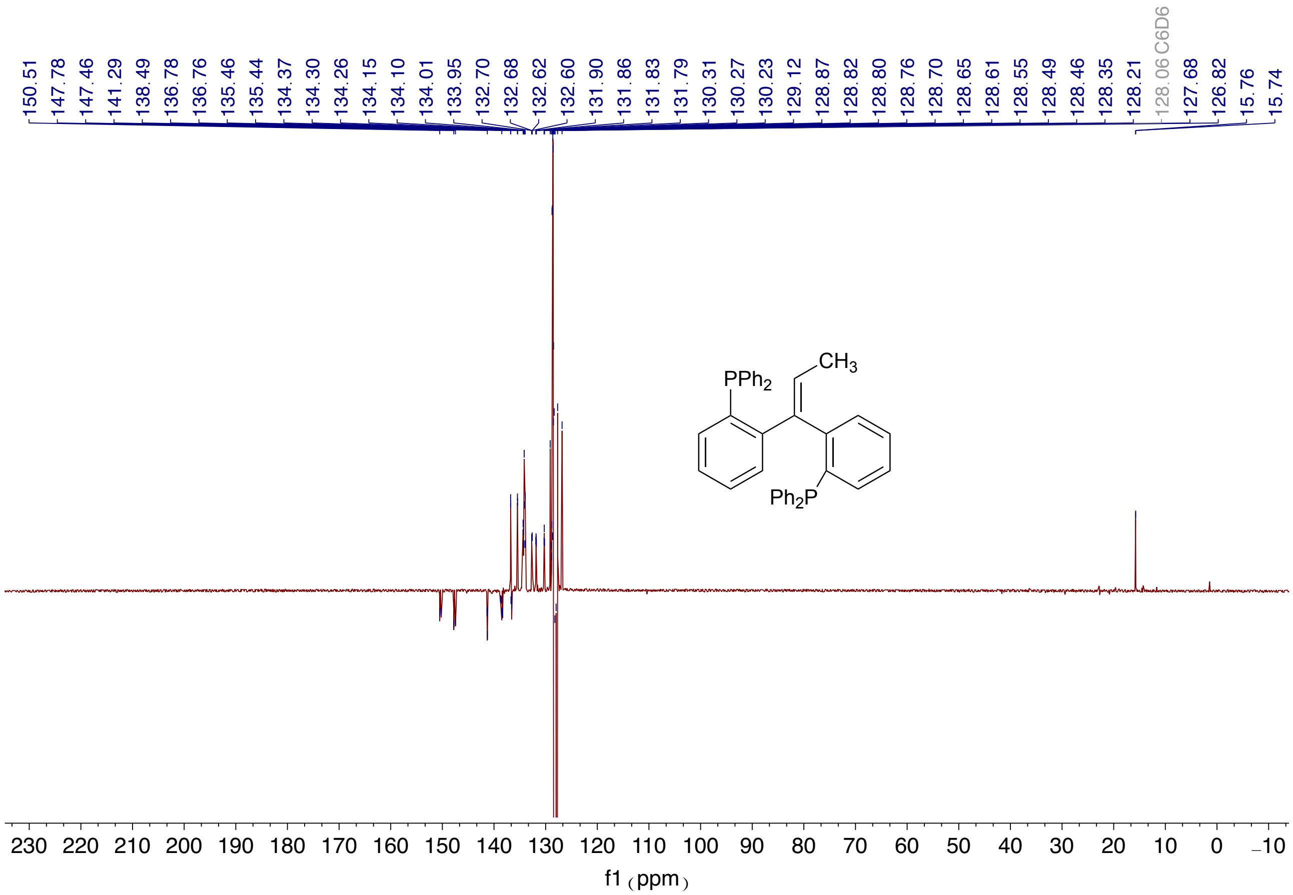

Figure S23. ^{13}C APT spectrum of ligand **4** in C_6D_6 at 25 °C.

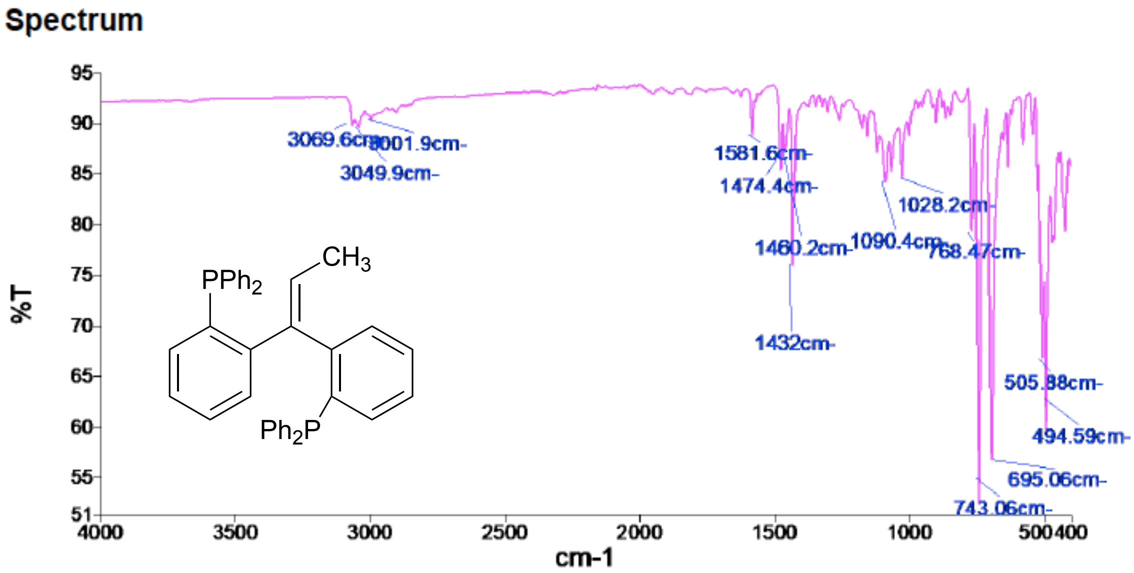

Figure S24. FTIR spectrum of ligand **4** at 25 °C.

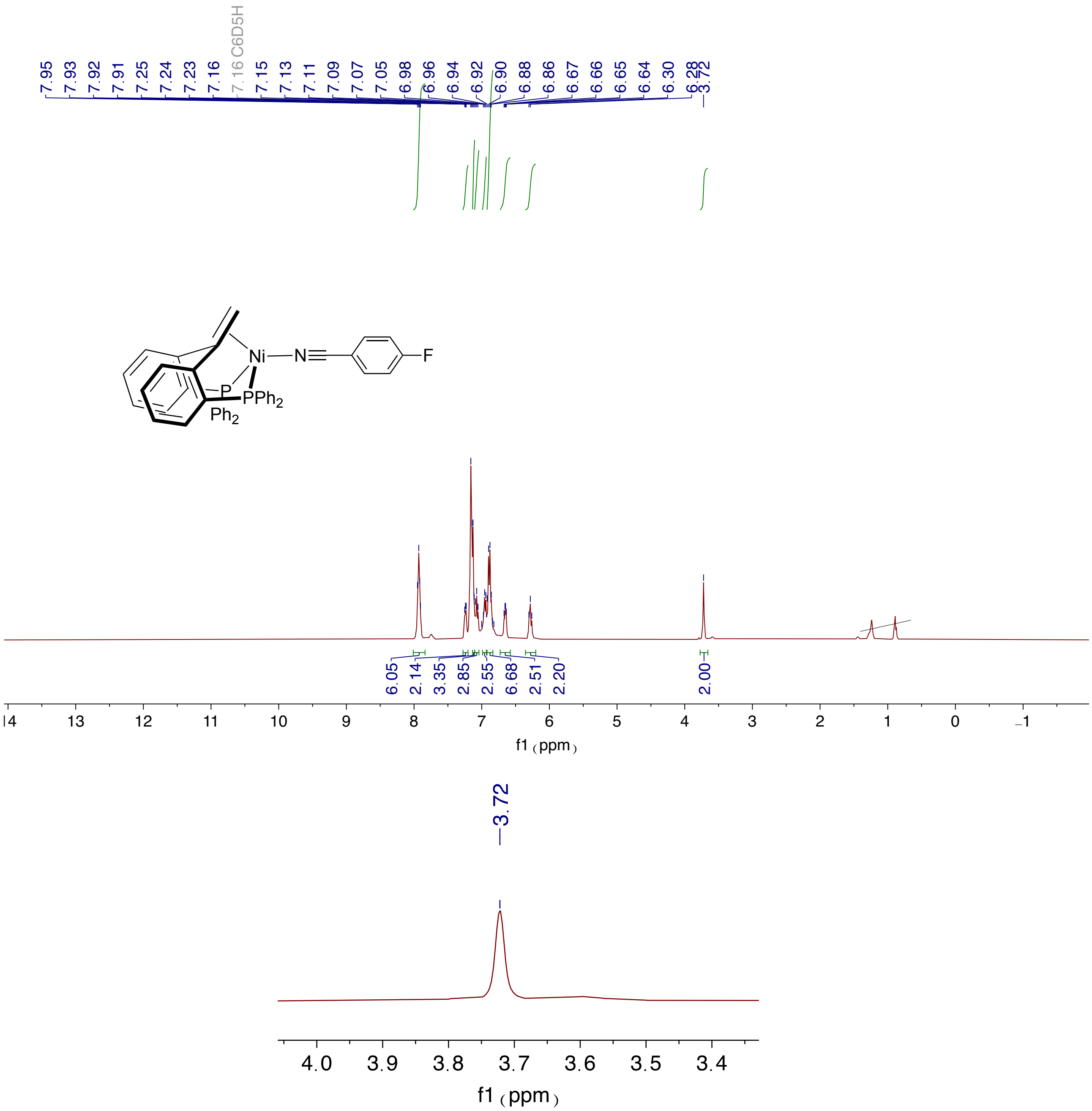

Figure S25. ^1H NMR spectrum of complex **5** in C_6D_6 at 25 ºC. Crossed peaks correspond to hexane.

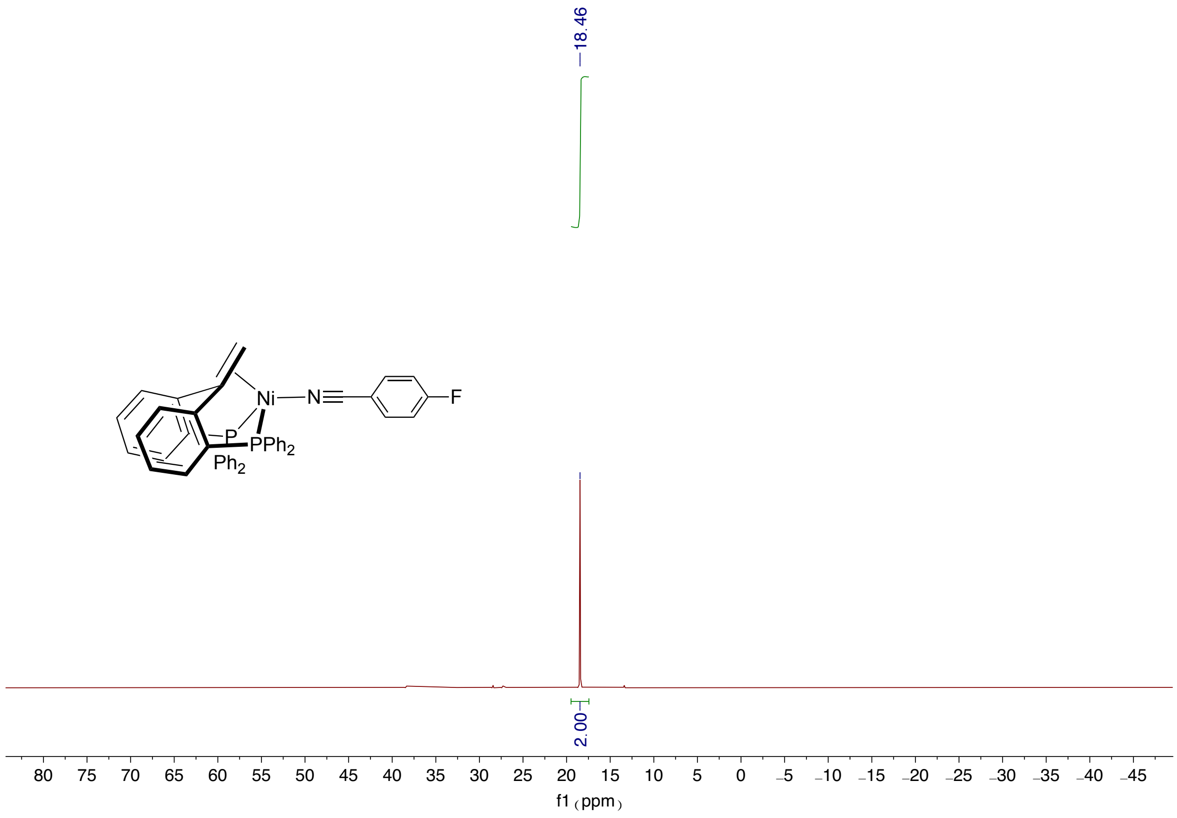

Figure S 26. $^{31}P\{^{1}H\}$ NMR spectrum of complex **5** in C_6D_6 at 25 ºC.

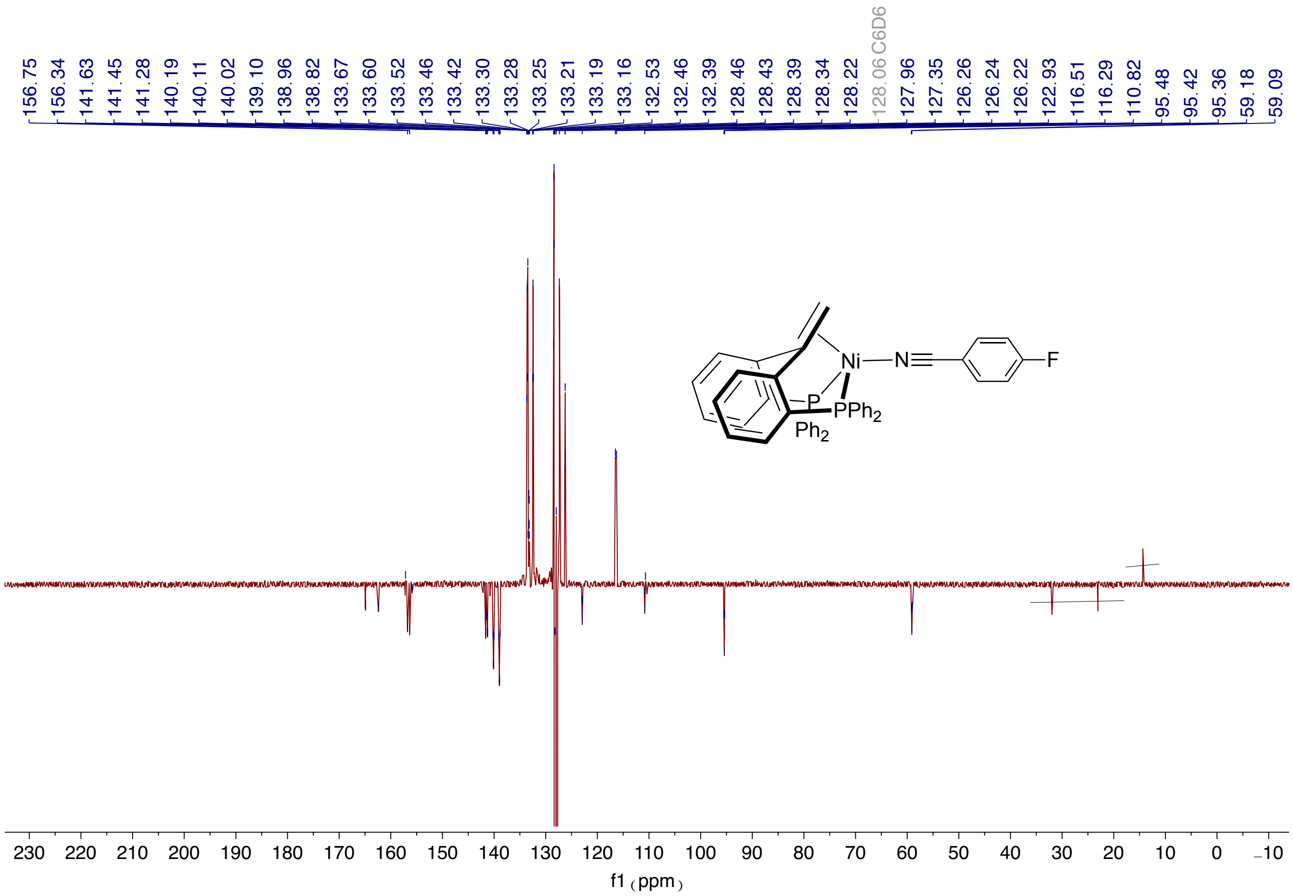

Figure S27. ^{13}C APT spectrum of complex **5** in C_6D_6 at 25 °C. Crossed peaks correspond to hexane.

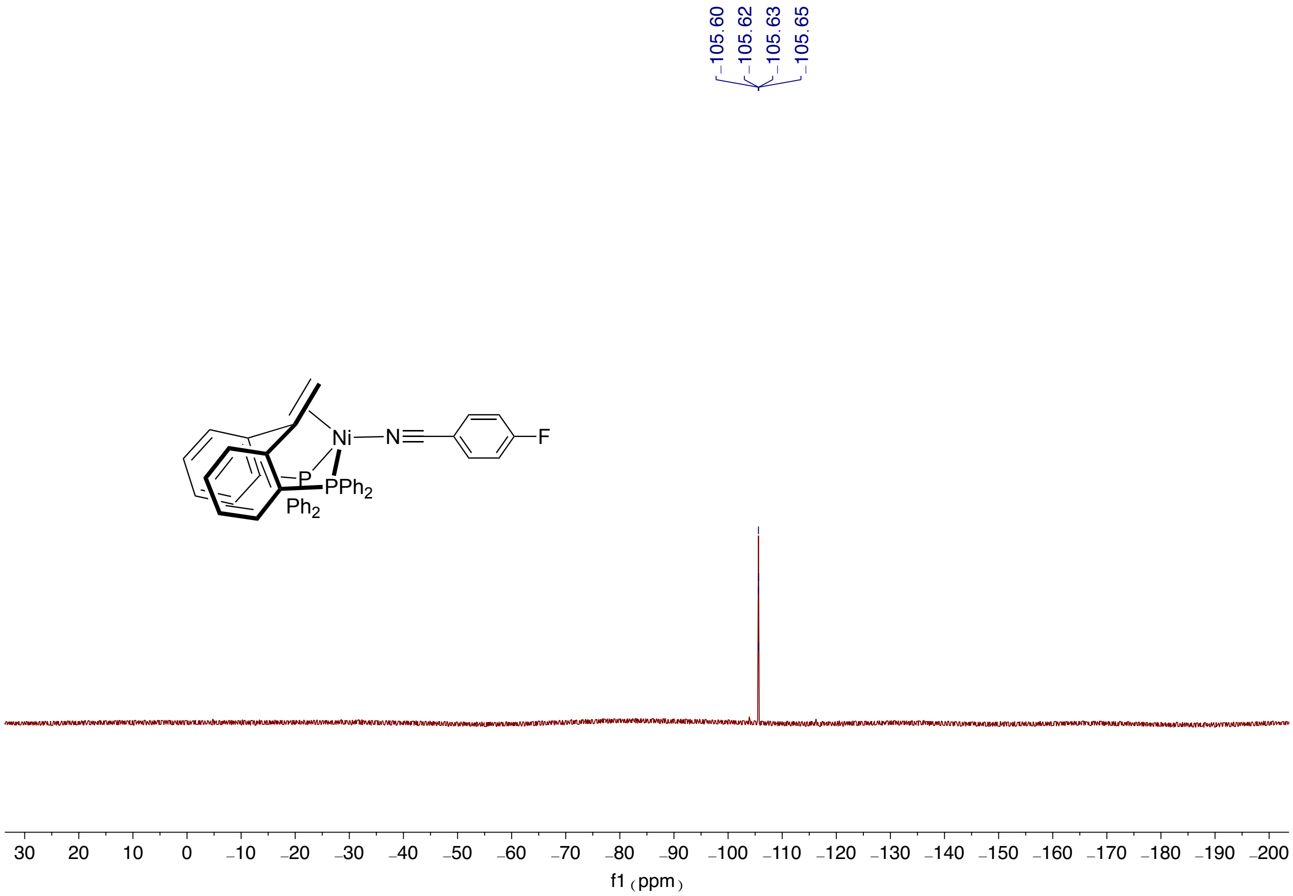

Figure S28. ^{19}F NMR spectrum of complex **5** in C_6D_6 at 25 °C.

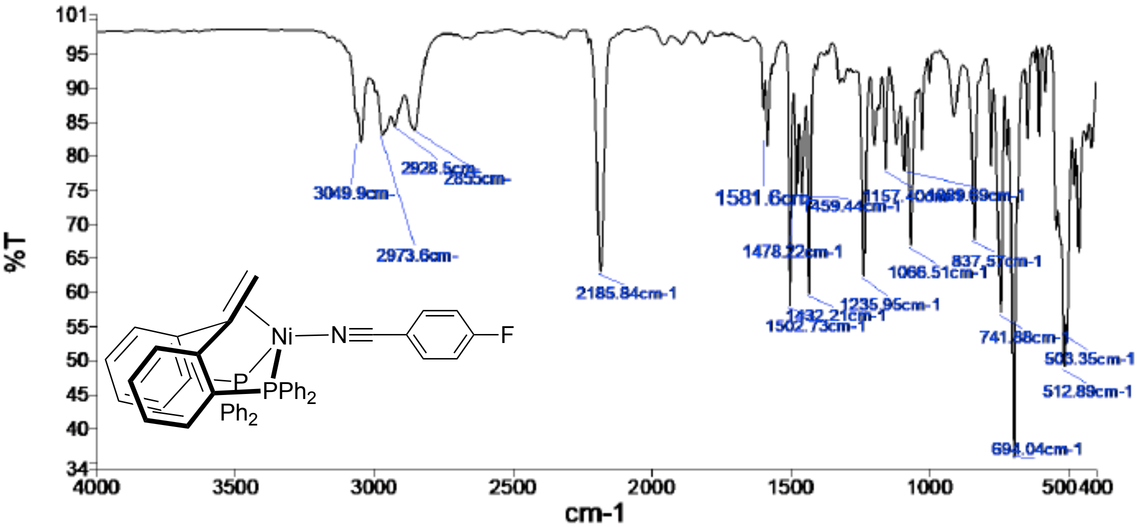

Figure S29. FTIR spectrum of complex **5** at 25 °C.

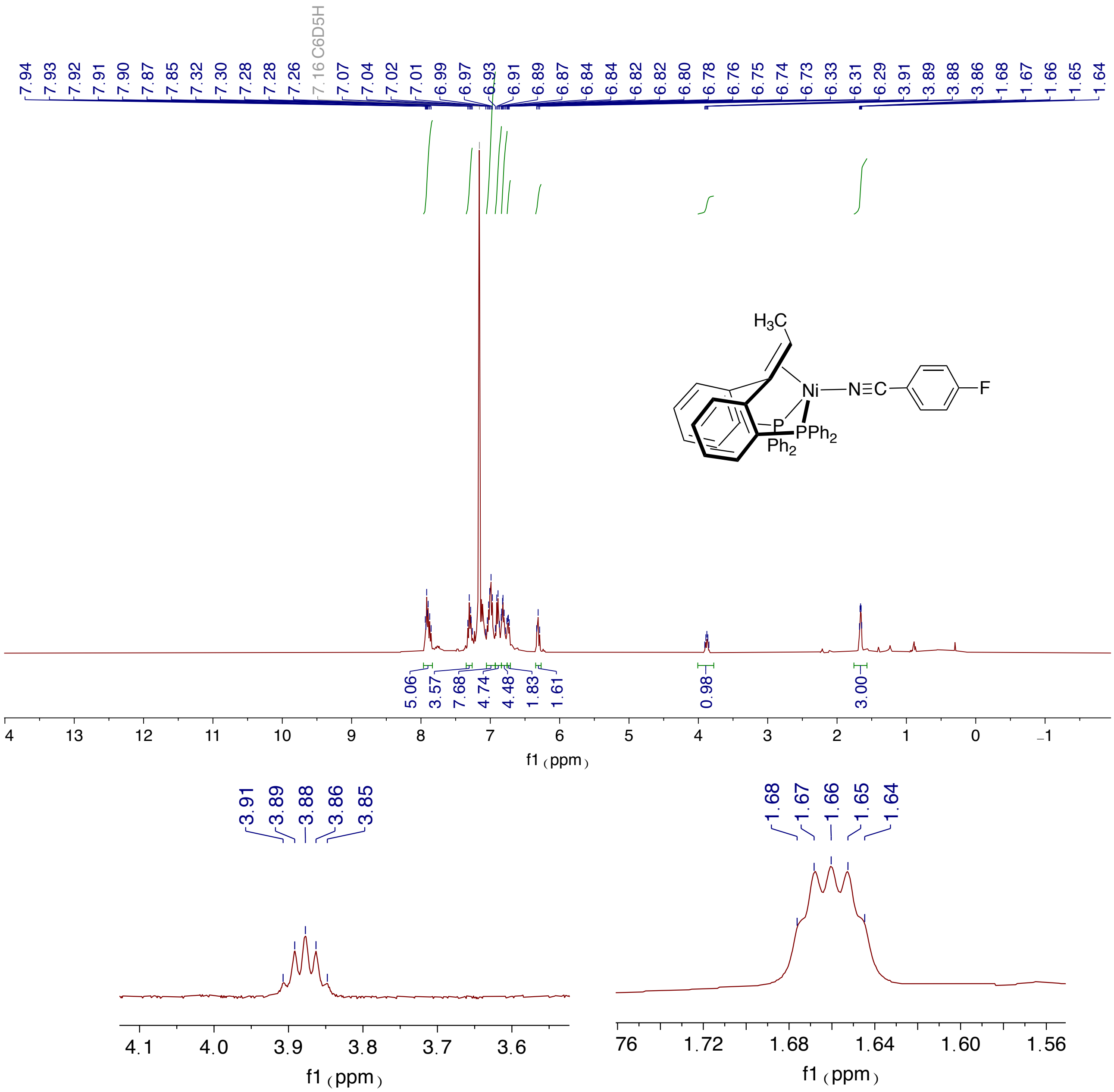

Figure S30. ^{1}H NMR spectrum of complex **6** in C_6D_6 at 25 °C.

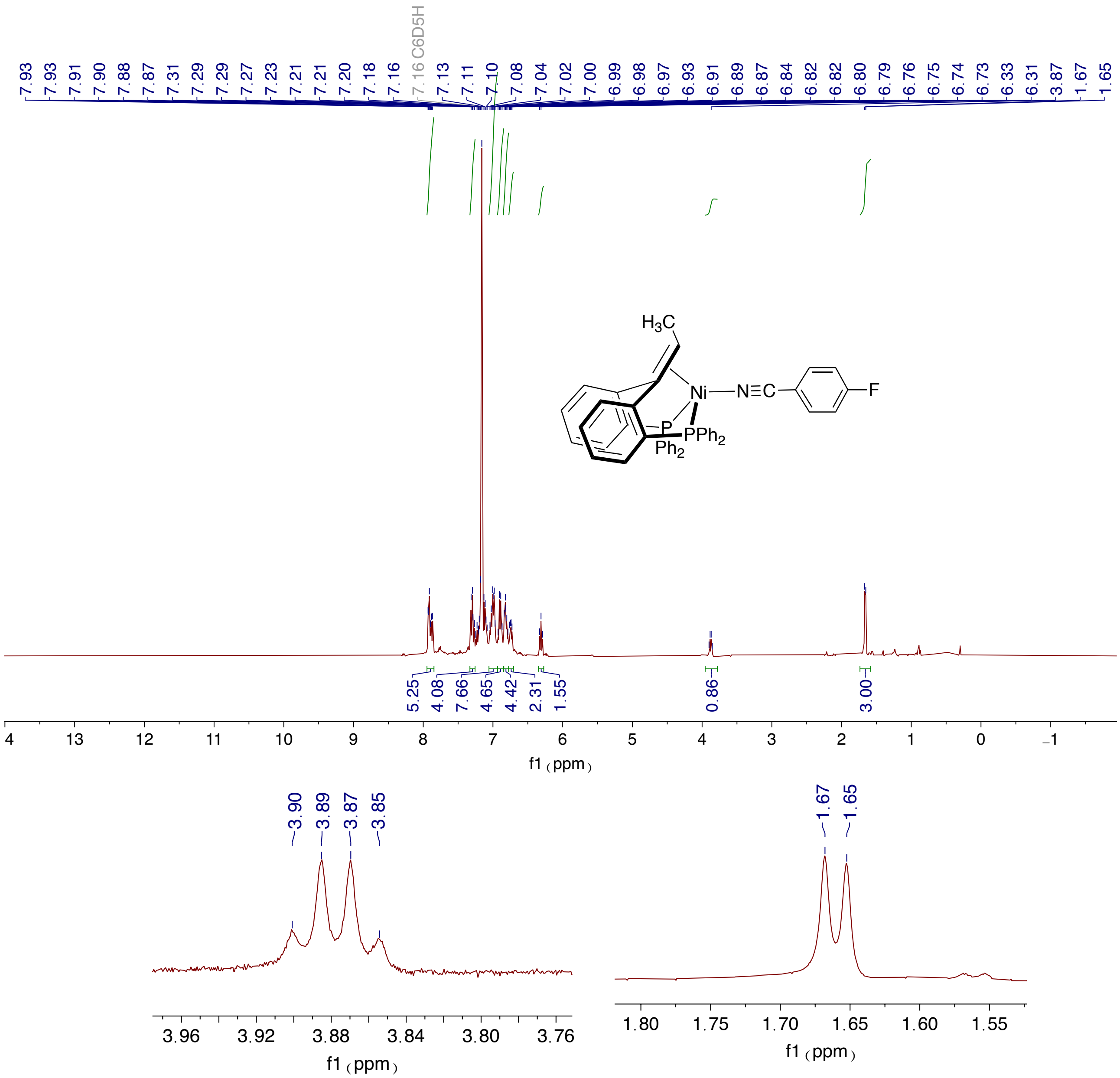

Figure S31. $^{1}H\{^{31}P\}$ NMR spectrum of complex **6** in C_6D_6 at 25 °C.

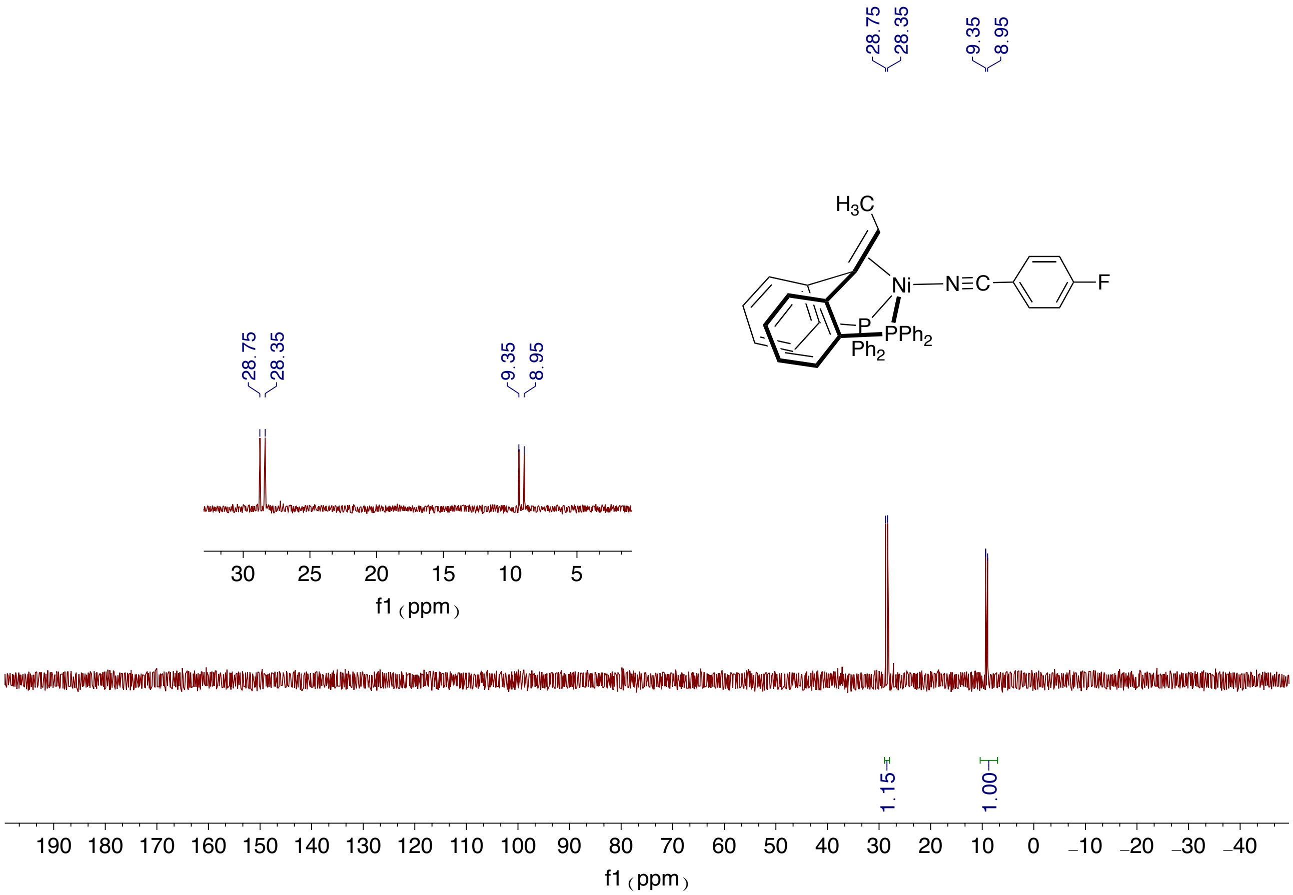

Figure S32. ^{31}P NMR spectrum of complex **6** in C_6D_6 at 25 ºC.

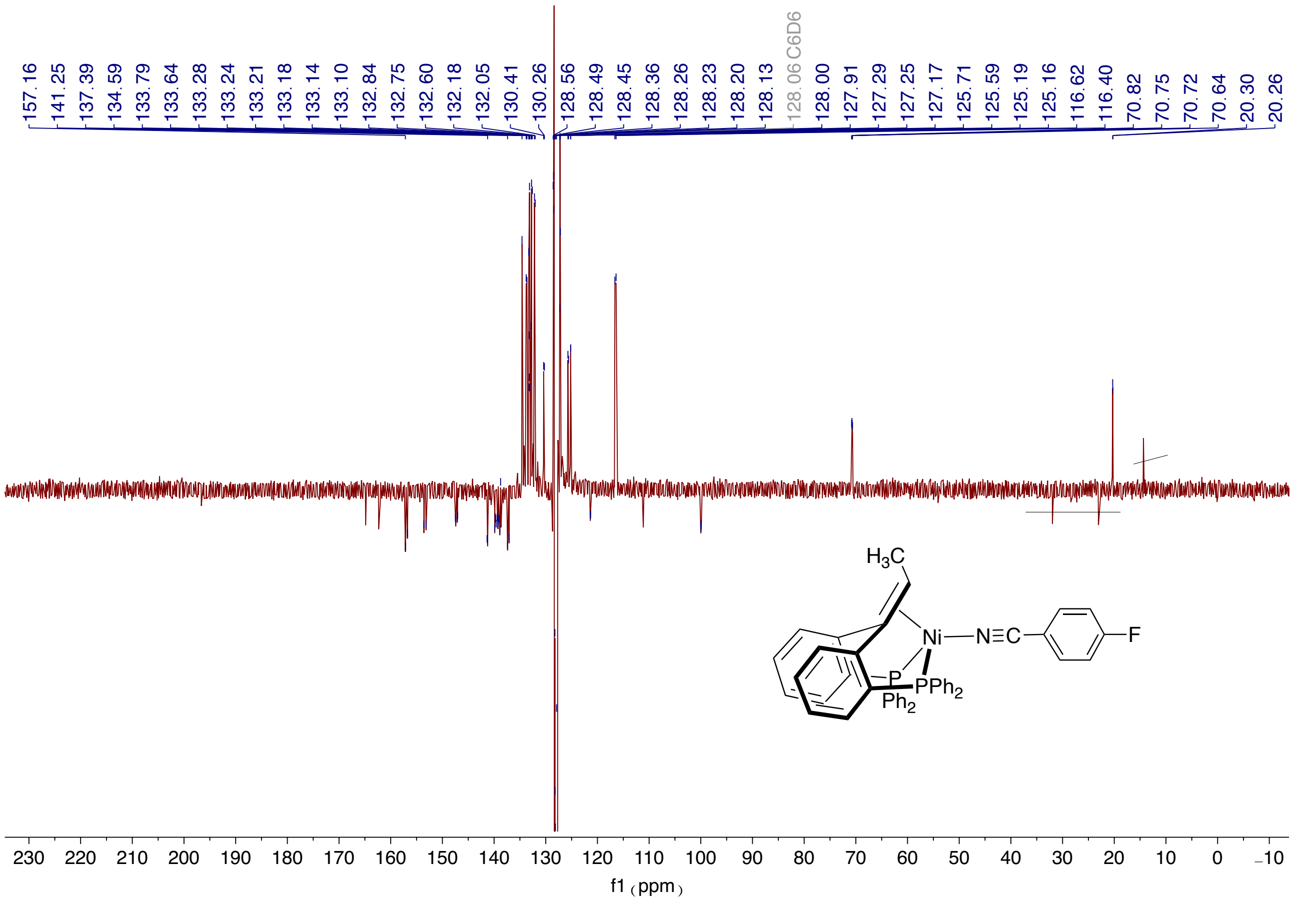

Figure S33. ^{13}C APT spectrum of complex **6** in C_6D_6 at 25 ºC. Crossed peaks correspond to hexane.

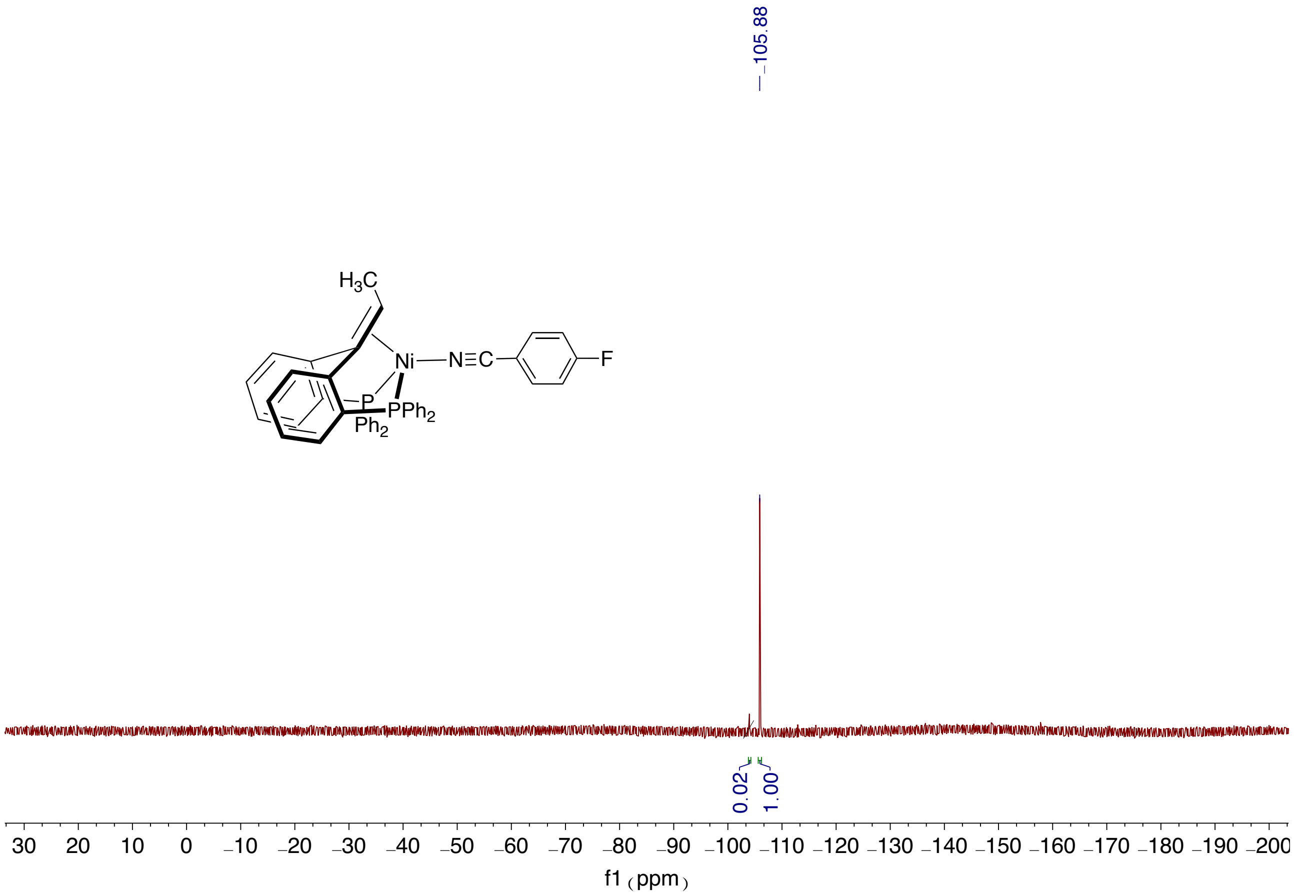

Figure S34. ^{19}F NMR spectrum of complex **6** in C_6D_6 at 25 ºC.

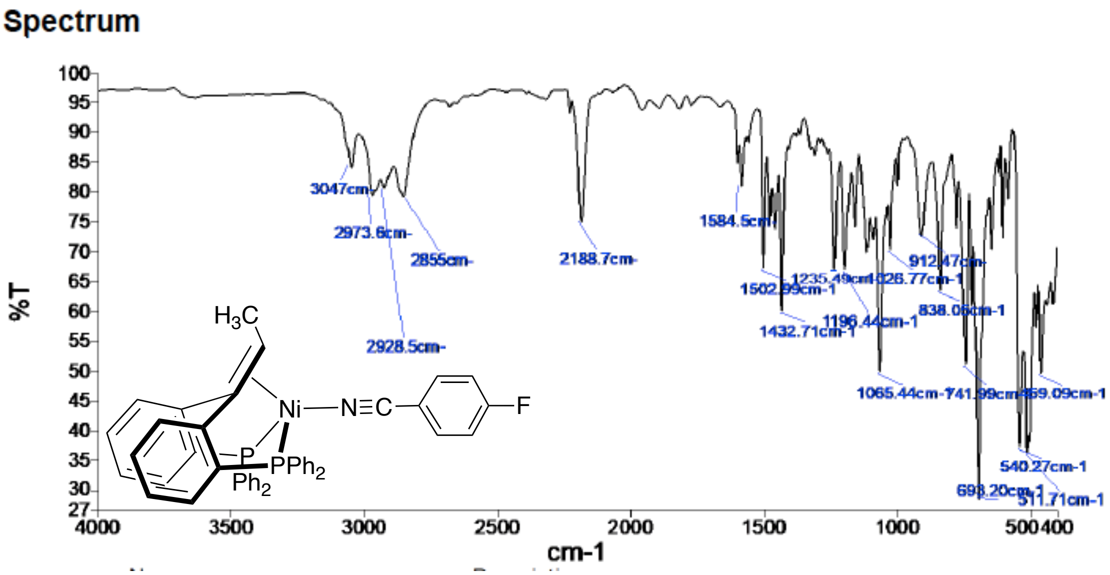

Figure S35. FTIR spectrum of complex **6** at 25 ºC.

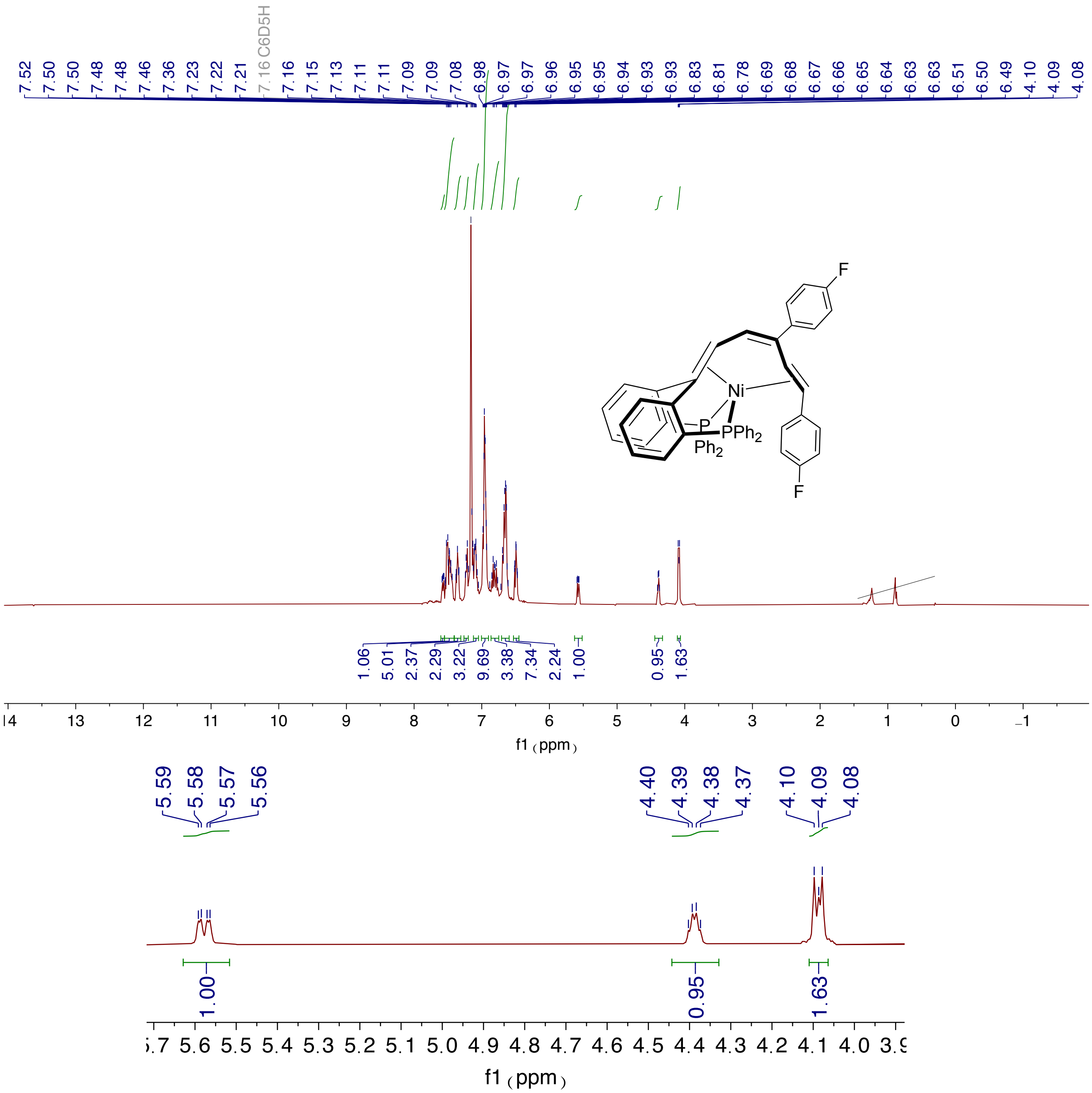

Figure S36. ^1H NMR spectrum of complex **7** in C_6D_6 at 25 ºC. Crossed peaks correspond to hexane.

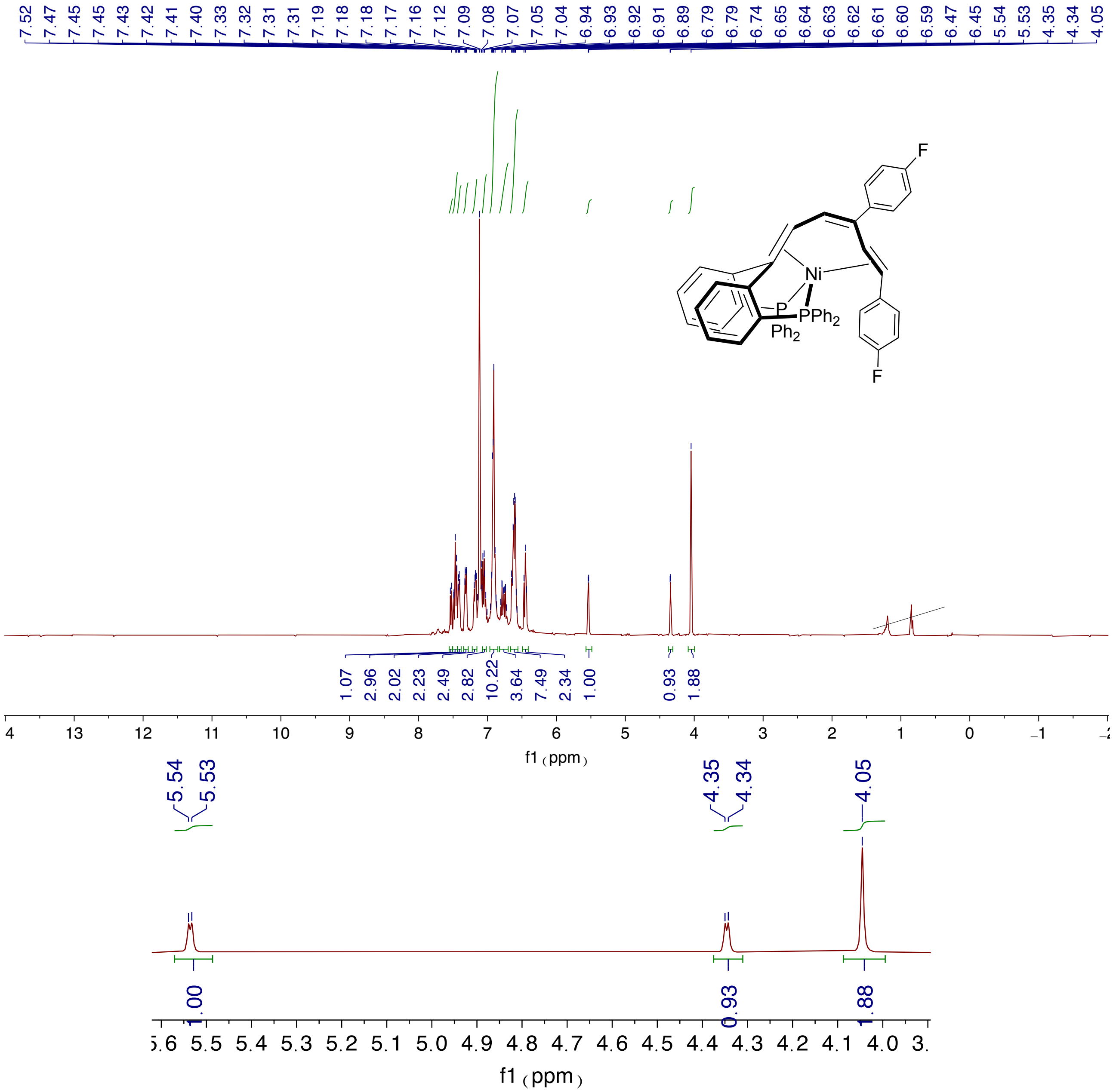

Figure S37. $^{1}H\{^{31}P\}$ NMR spectrum of complex **7** in C_6D_6 at 25 °C. Crossed peaks correspond to hexane.

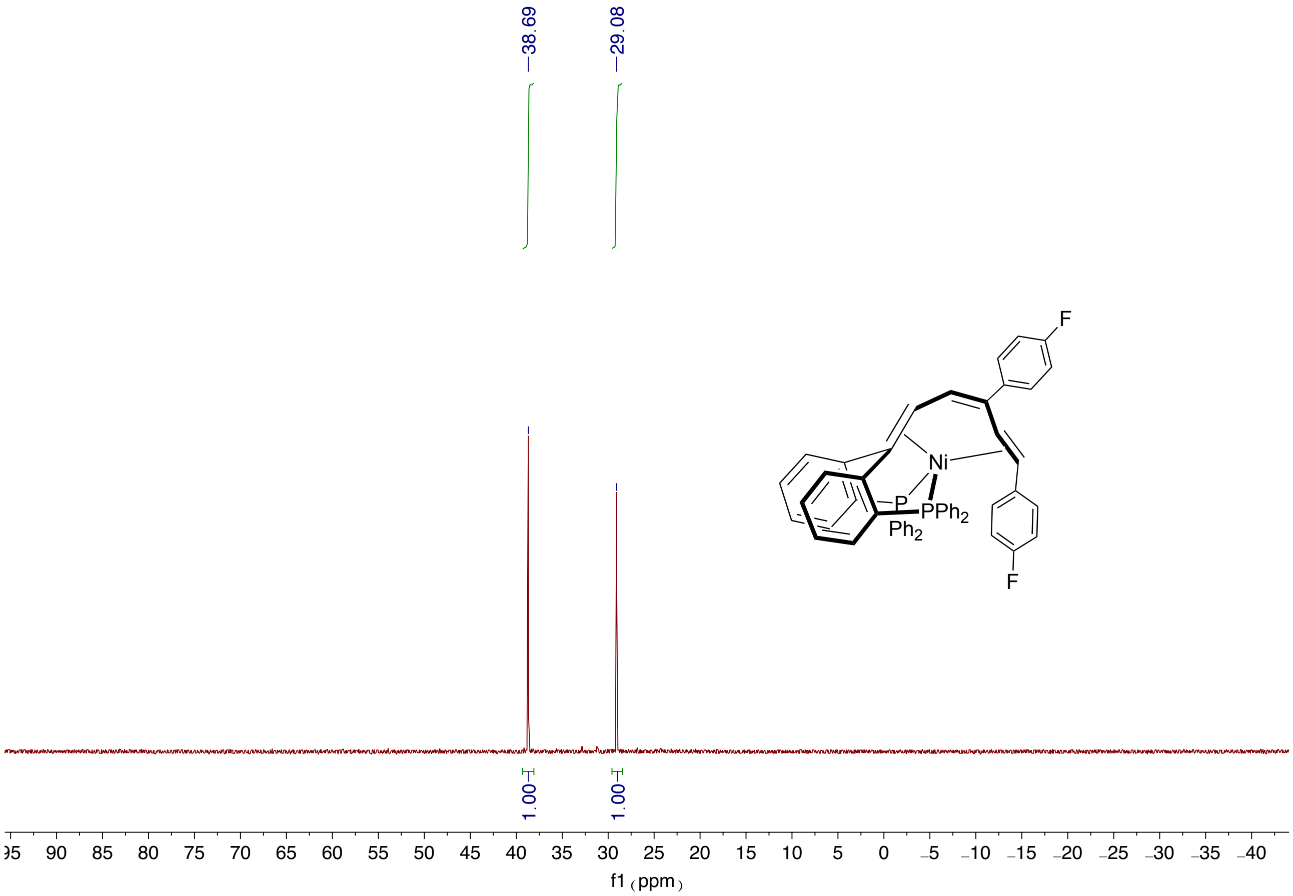

Figure S38. ^{31}P NMR spectrum of complex **7** in C_6D_6 at 25 ºC.

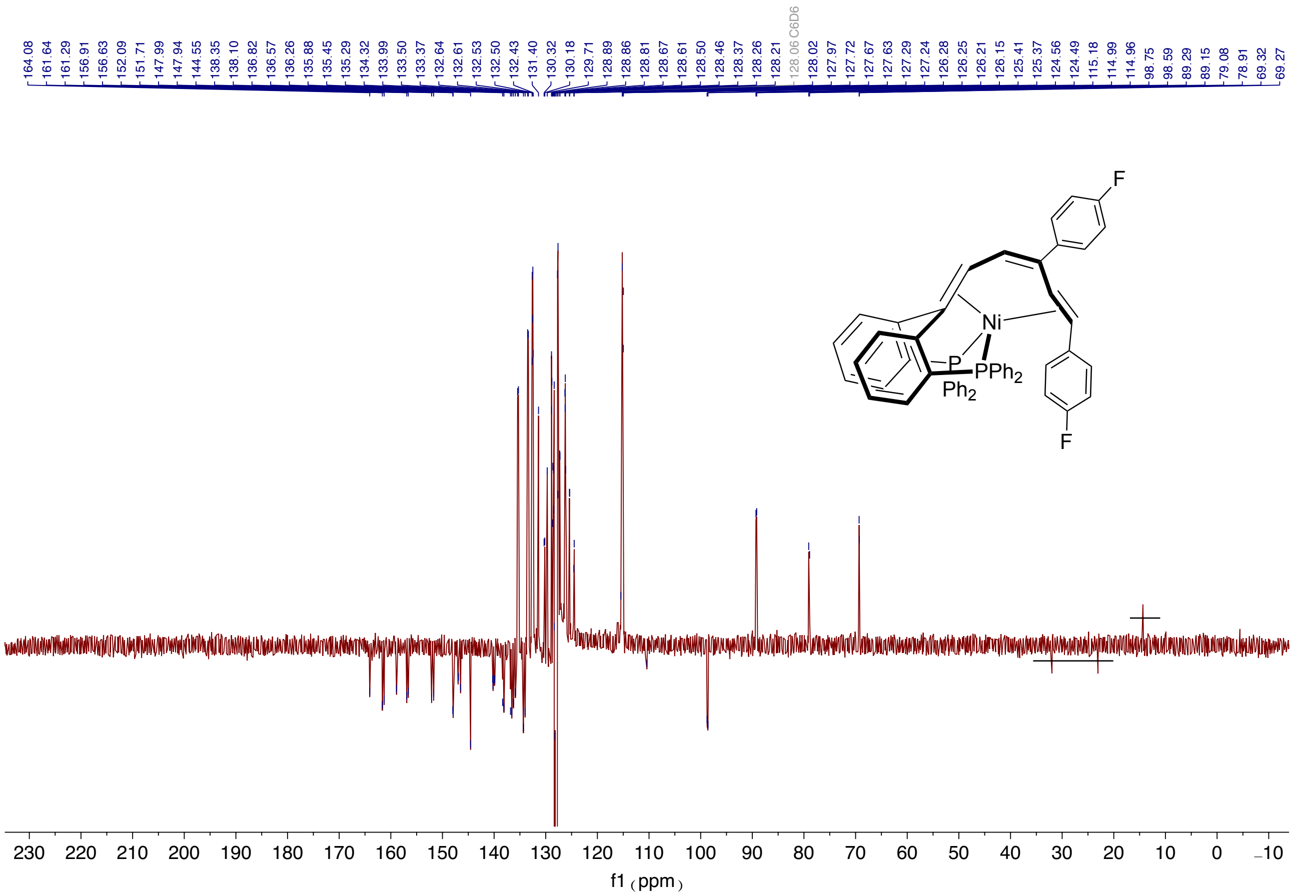

Figure S39. ${}^{13}C$ APT spectrum of complex **7** in C_6D_6 at 25 ºC.

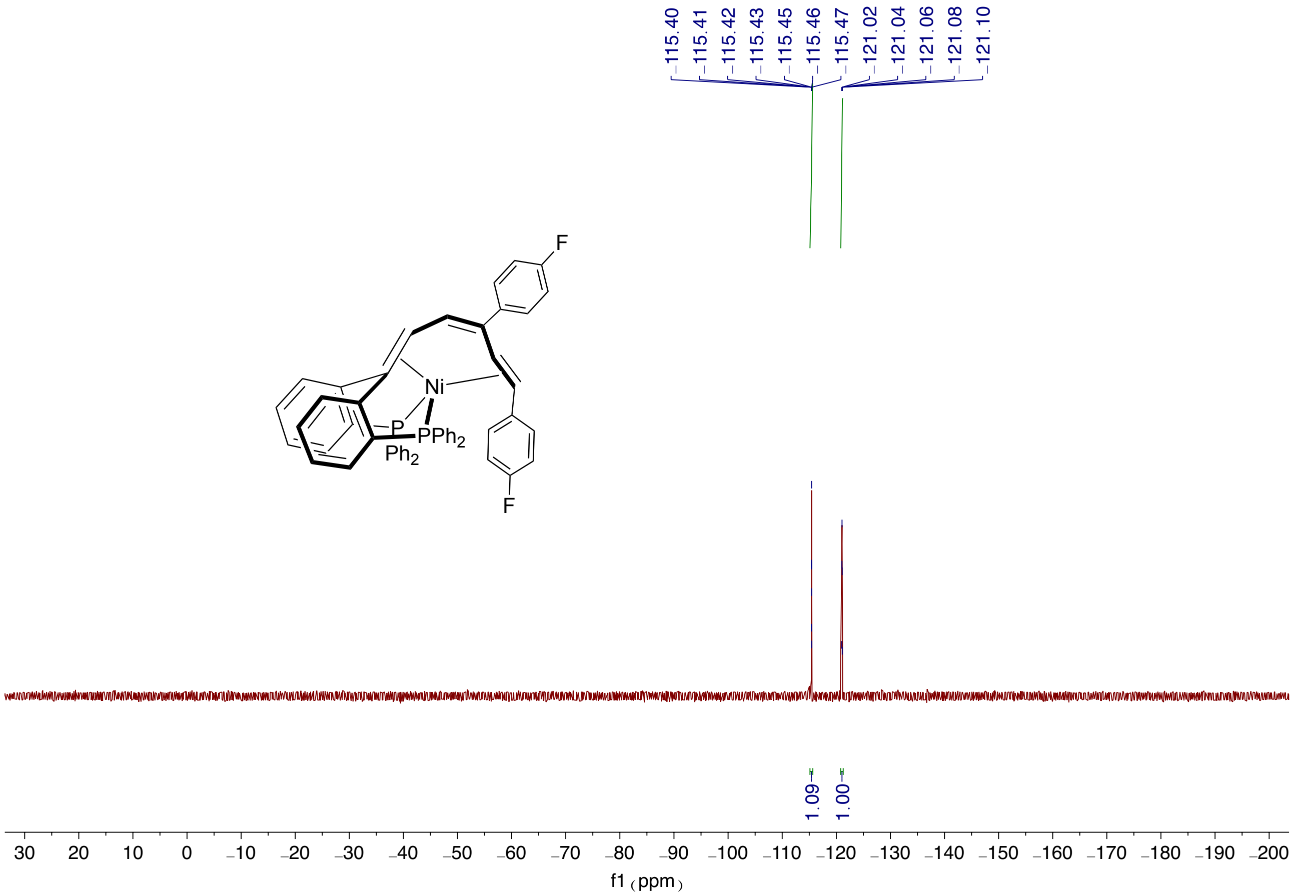

Figure S40. ^{19}F NMR spectrum of complex **7** in C_6D_6 at 25 ºC.

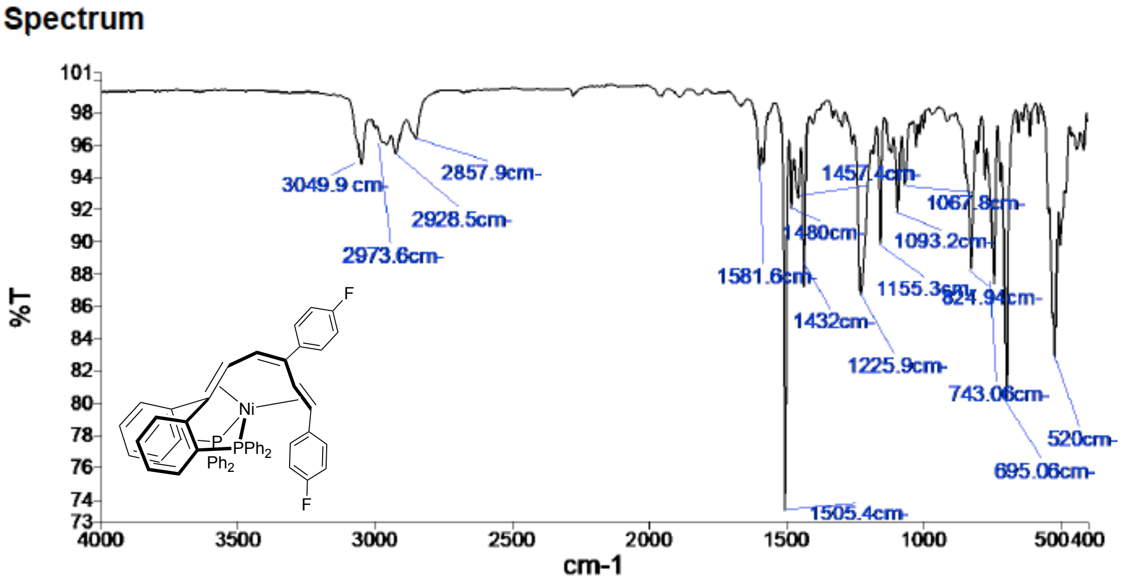

Figure S41. FTIR spectrum of complex **7** at 25 ºC.

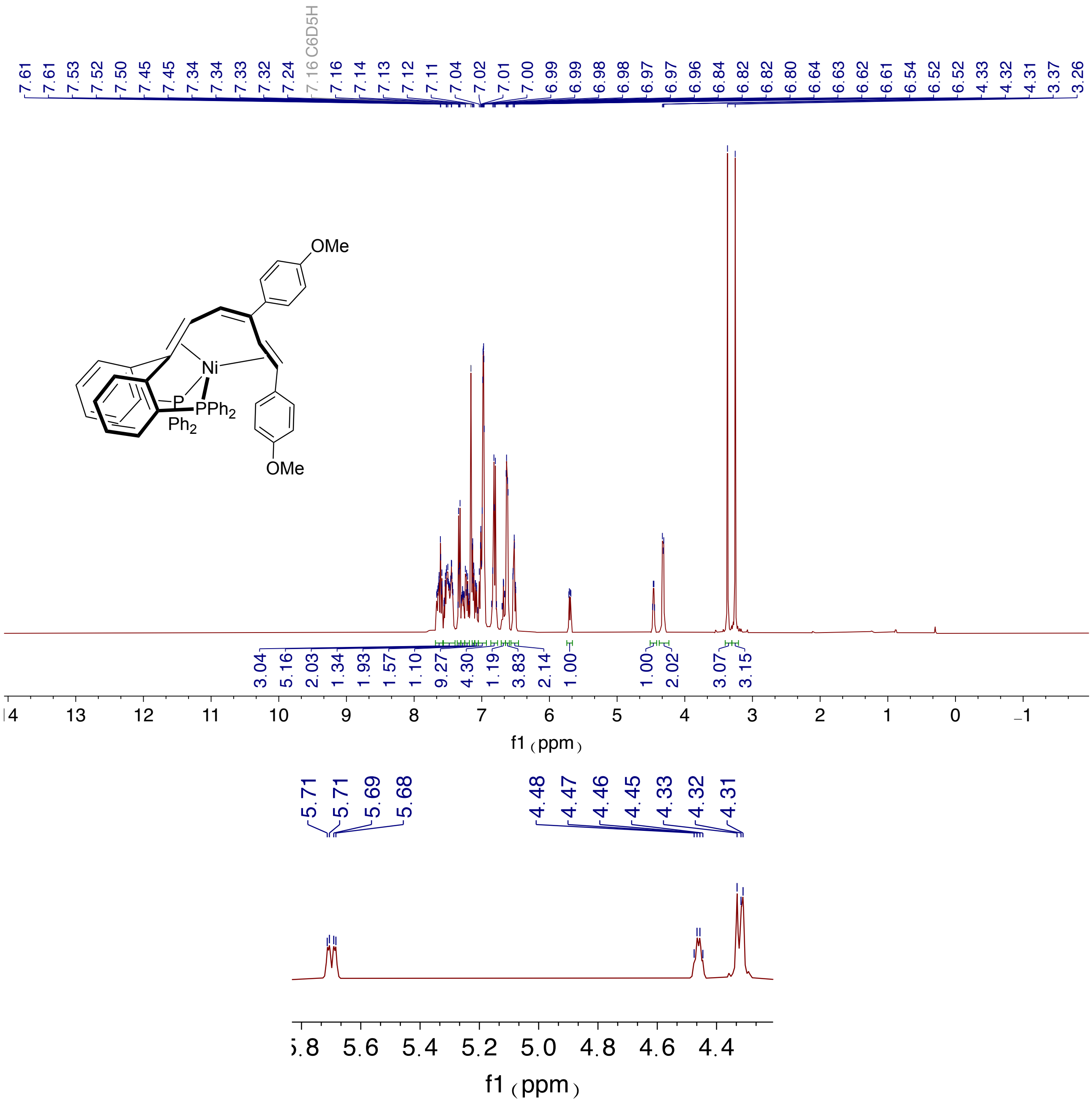

Figure S42. ^1H NMR spectrum of complex **8** in C_6D_6 at 25 ºC

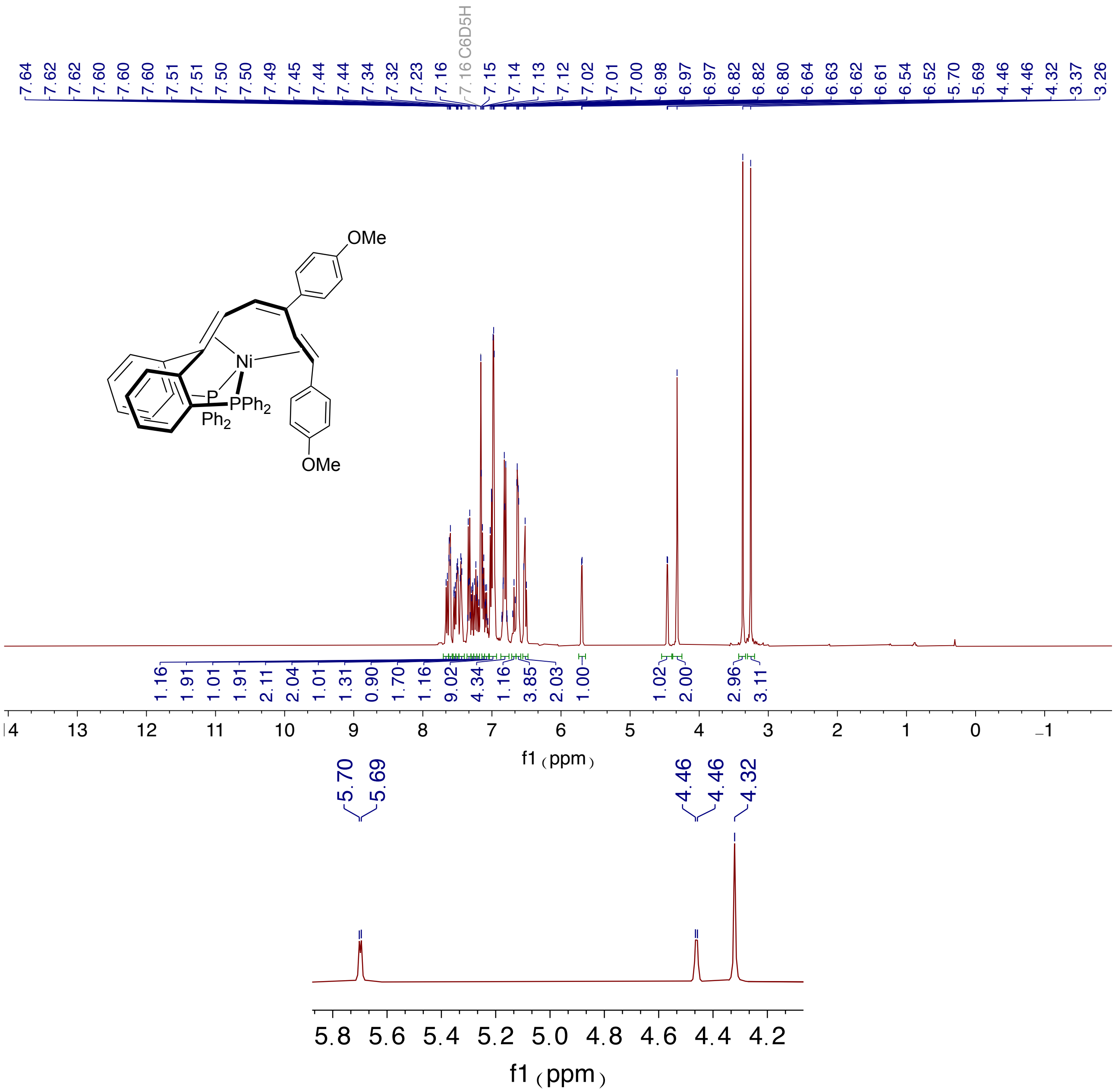

Figure S43. $^{1}H\{^{31}P\}$ NMR spectrum of complex **8** in C_6D_6 at 25 °C.

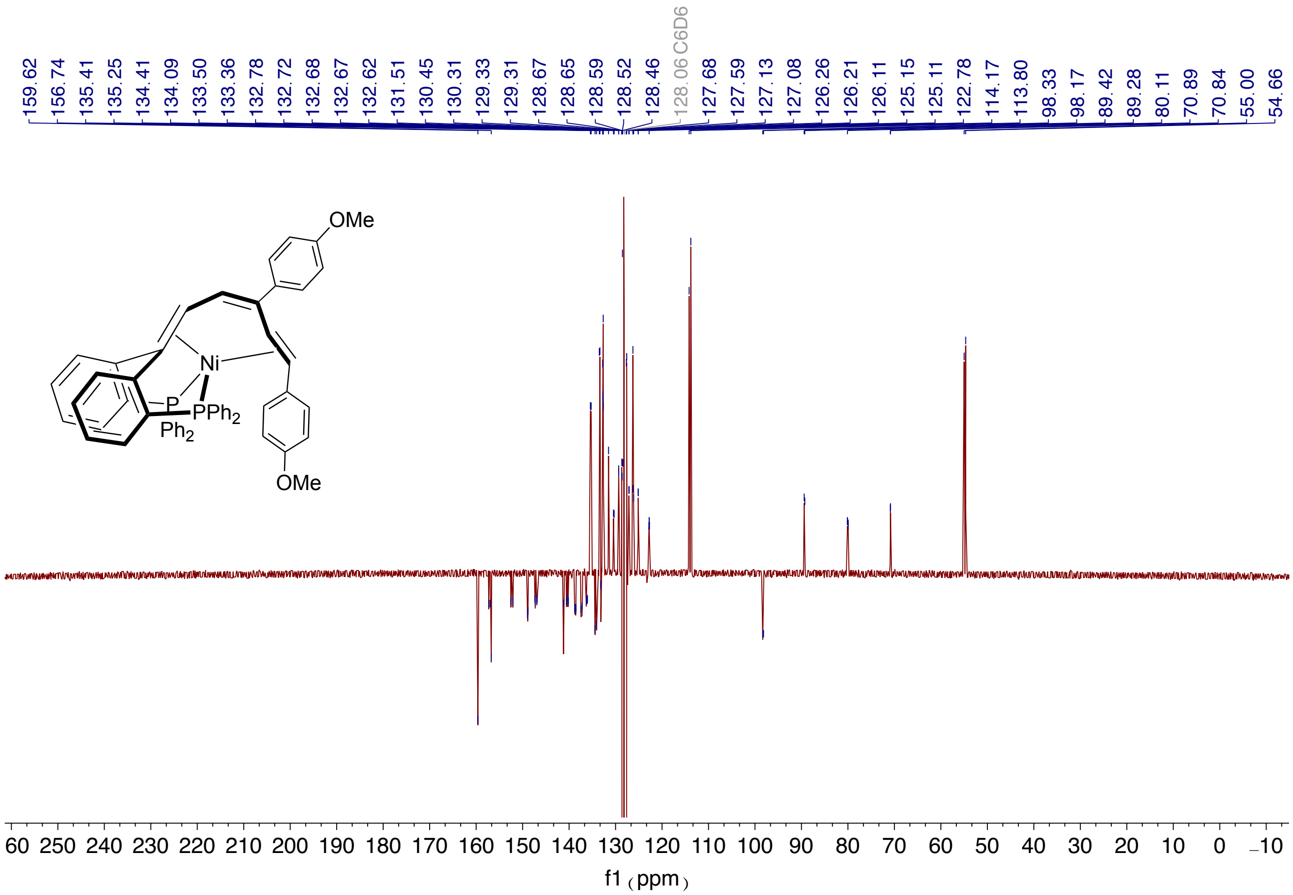

Figure S44. ^{13}C APT spectrum of complex **8** in C_6D_6 at 25 ºC

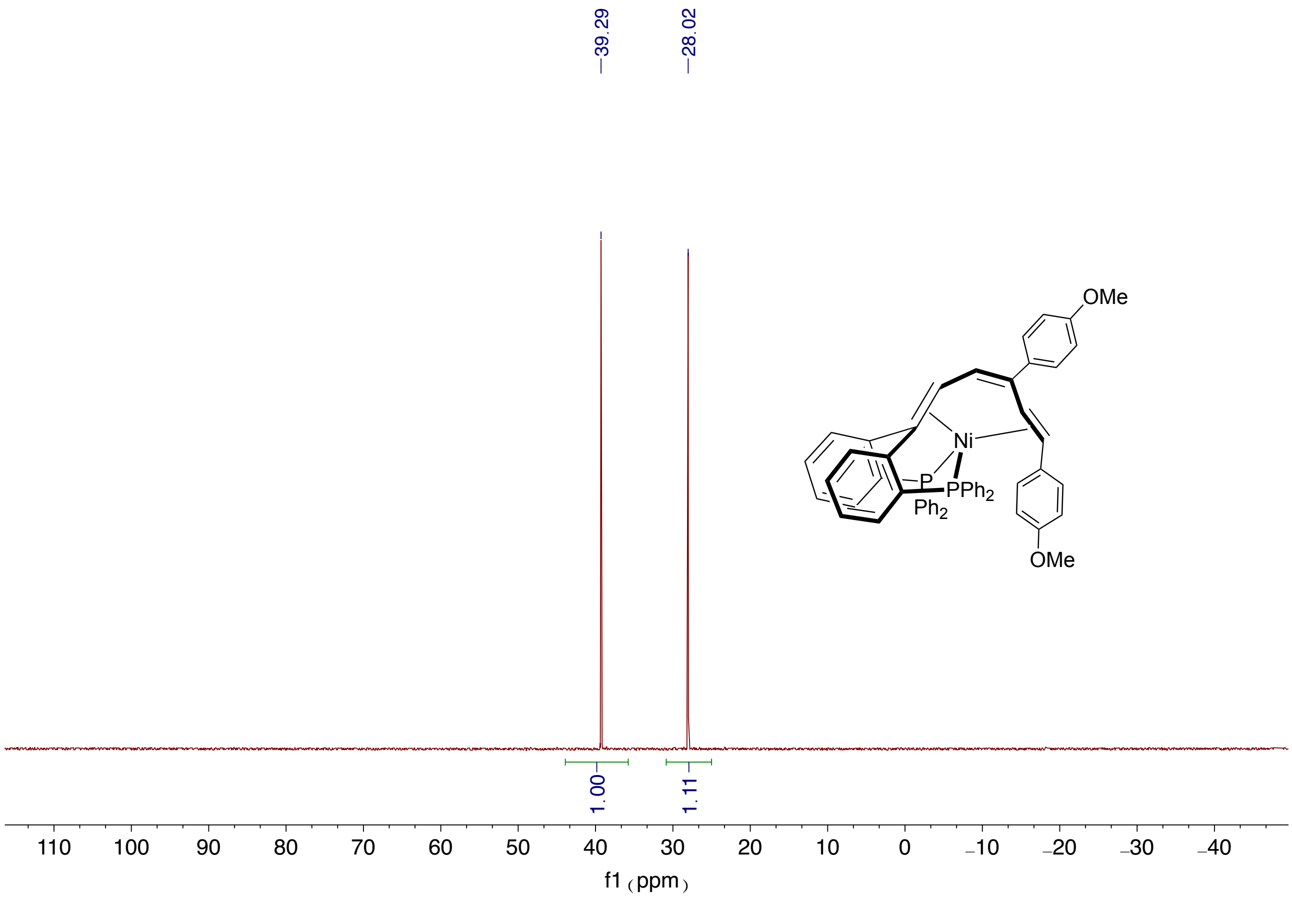

Figure S45. $^{31}P\{^{1}H\}$ NMR spectrum of complex **8** in C_6D_6 at 25 ºC.

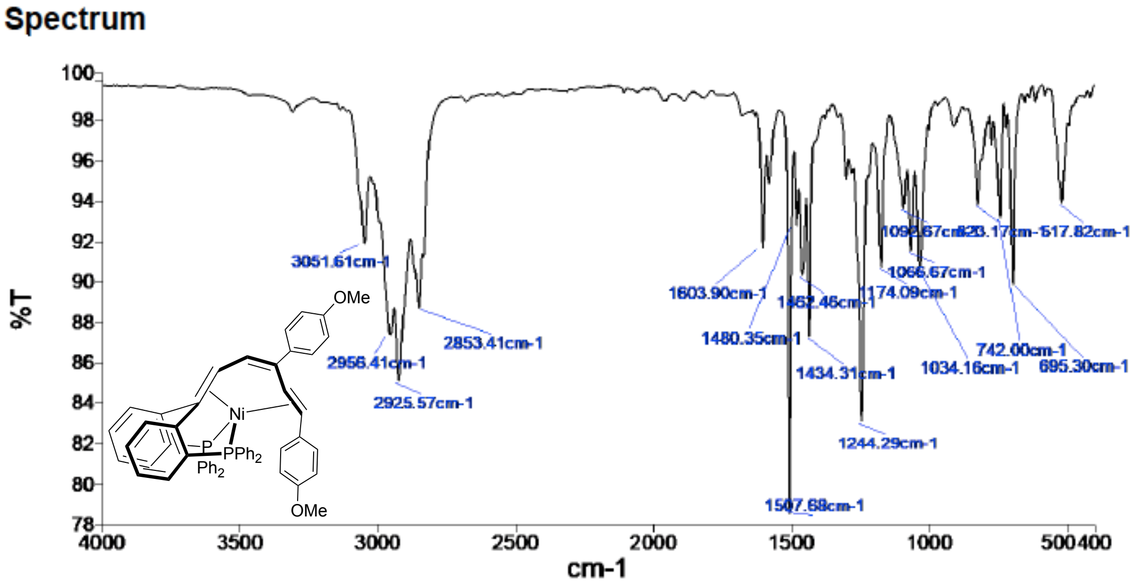

Figure S46. FTIR spectrum of complex **8** at 25 ºC.

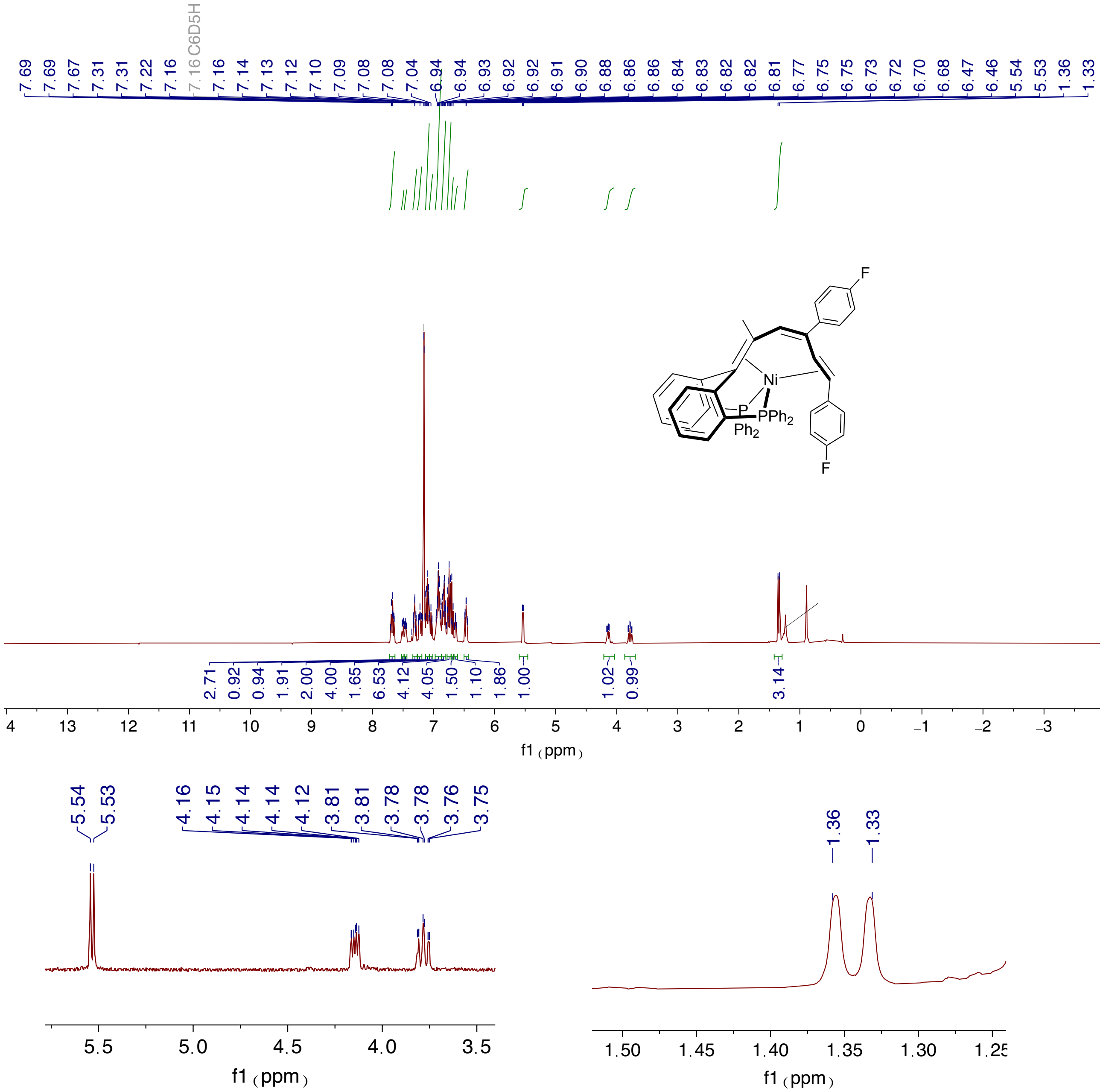

Figure S47. ^{1}H NMR spectrum of complex **9** in C_6D_6 at 25 ºC.

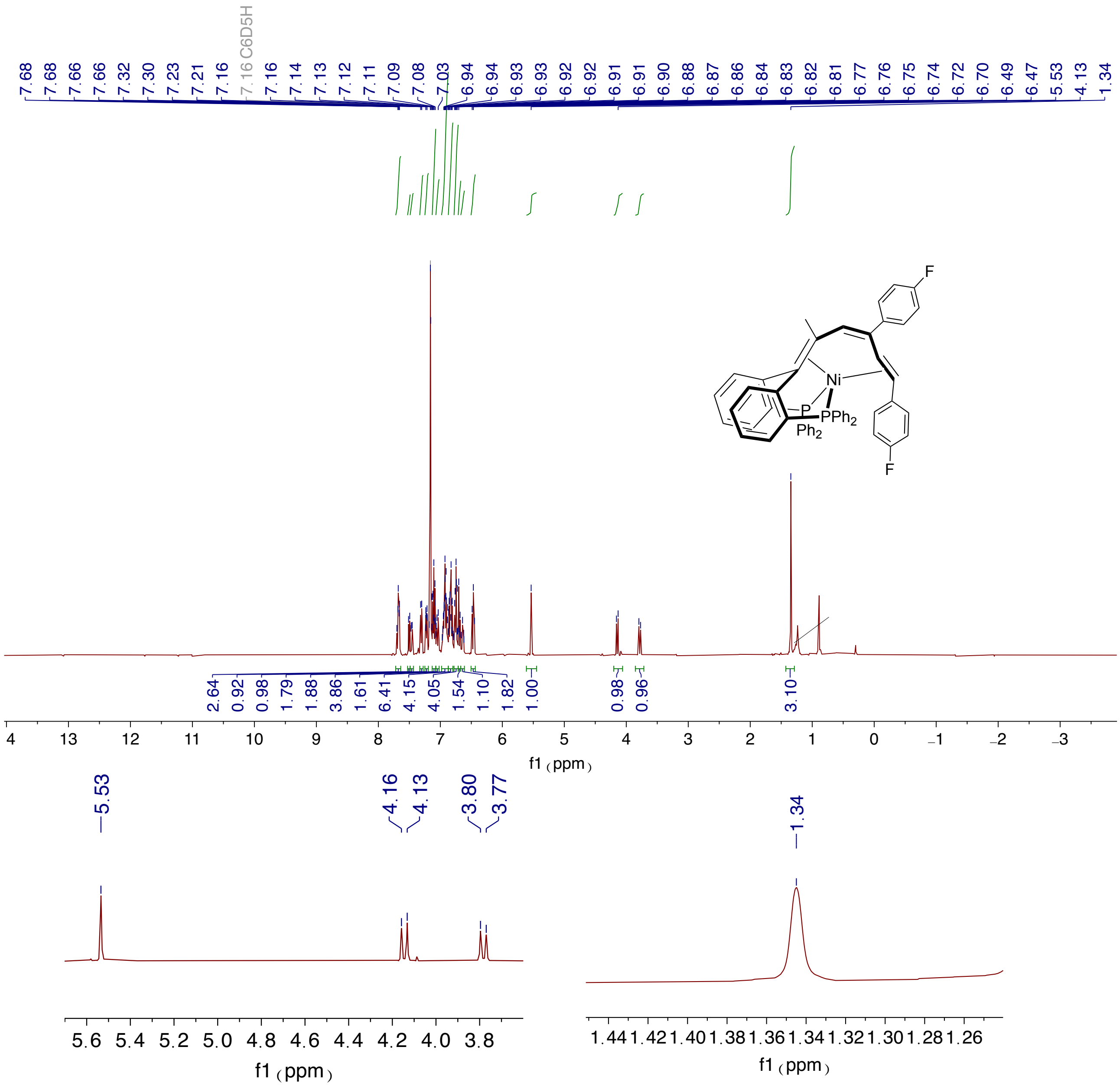

Figure S48. $^{1}H\{^{31}P\}$ NMR spectrum of complex **9** in C_6D_6 at 25 ºC.

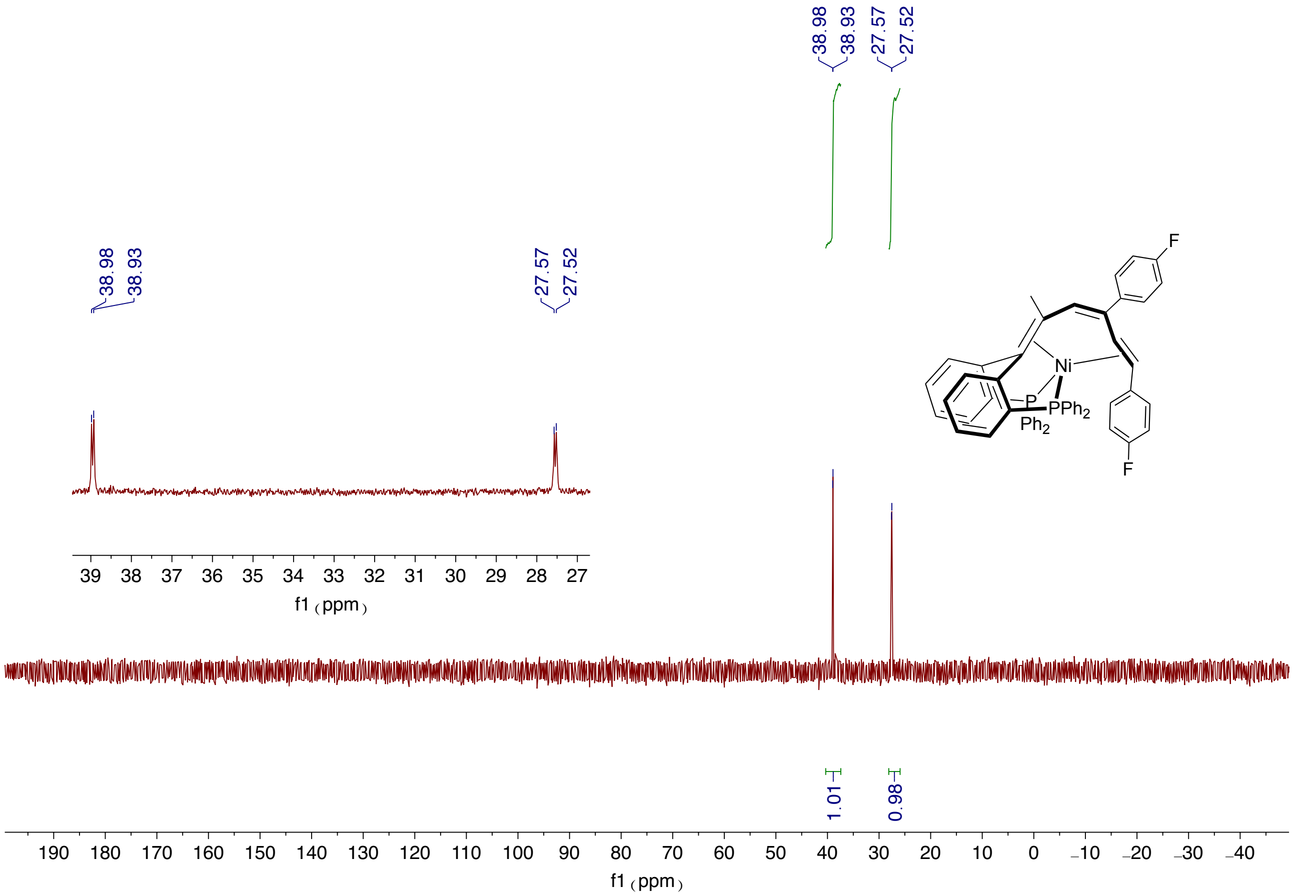

Figure S49. ^{31}P NMR spectrum of complex **9** in C_6D_6 at 25 ºC.

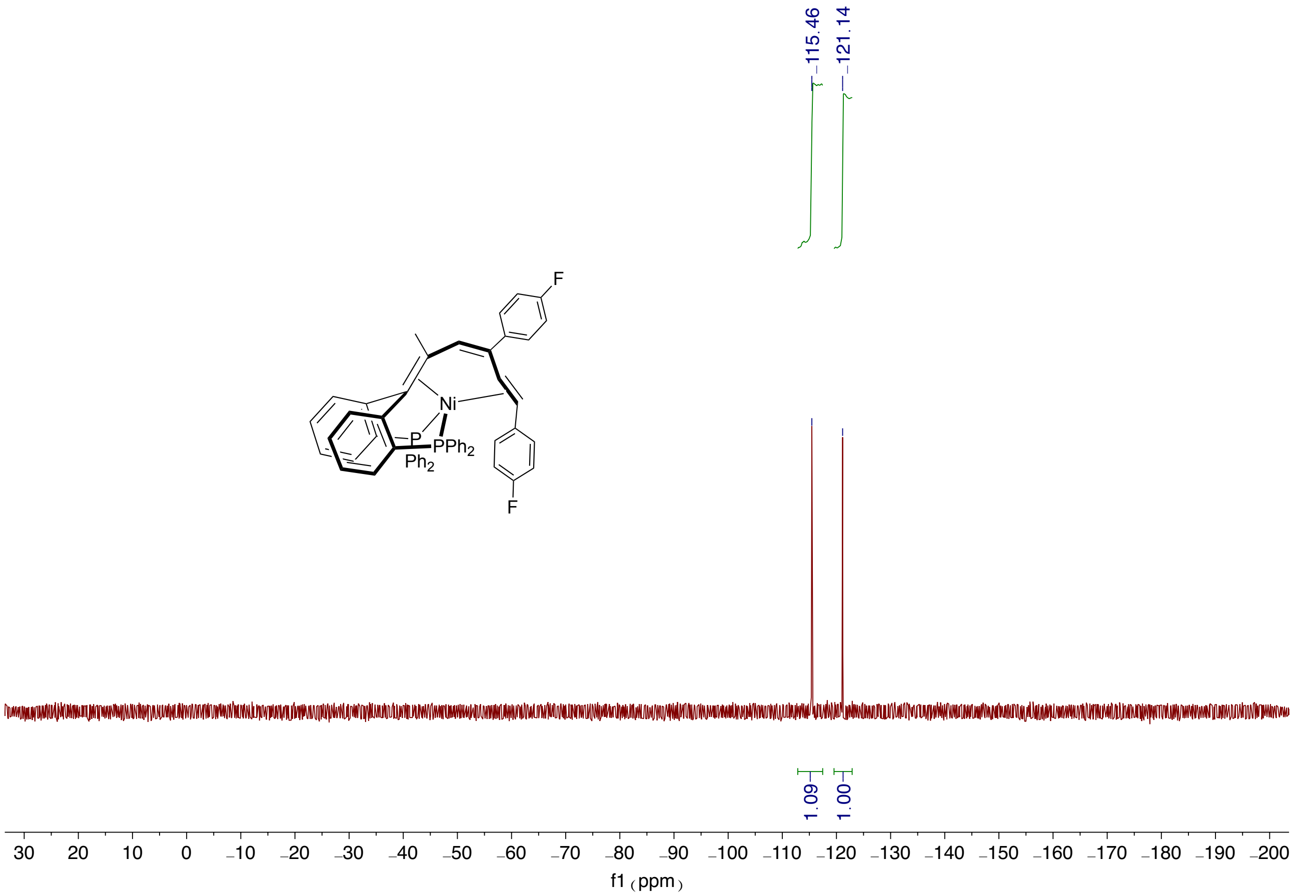

Figure S50. ${}^{19}F$ NMR spectrum of complex **9** in C_6D_6 at 25 ºC.

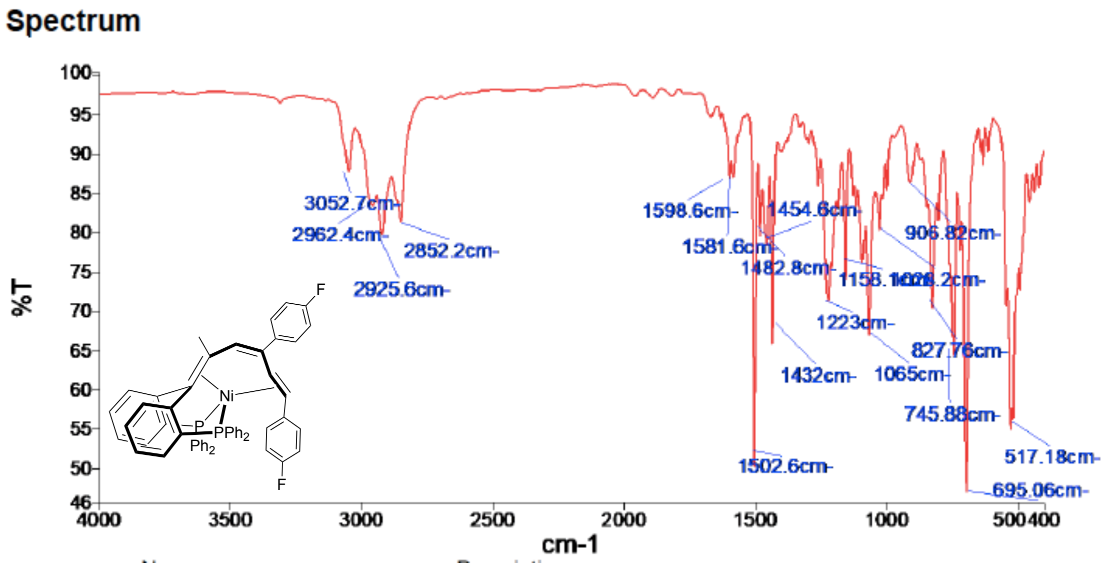

Figure S51. FTIR spectrum of complex **9** at 25 ºC.

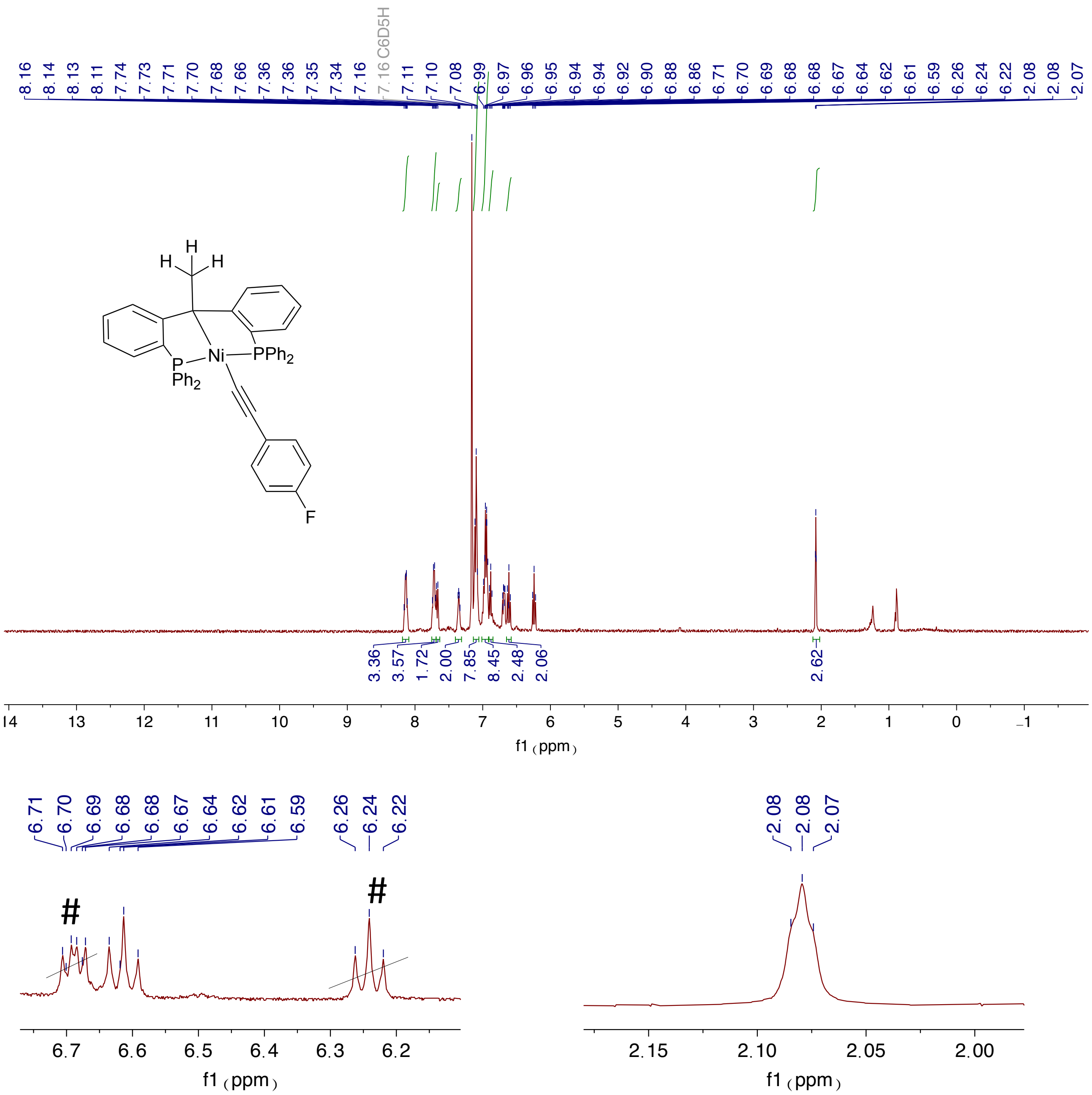

Figure S52. ^{1}H NMR spectrum of complex **10** in C_6D_6 at 25 ºC. Crossed peaks marked with (#) correspond to free 4-fluorobenzonitrile.

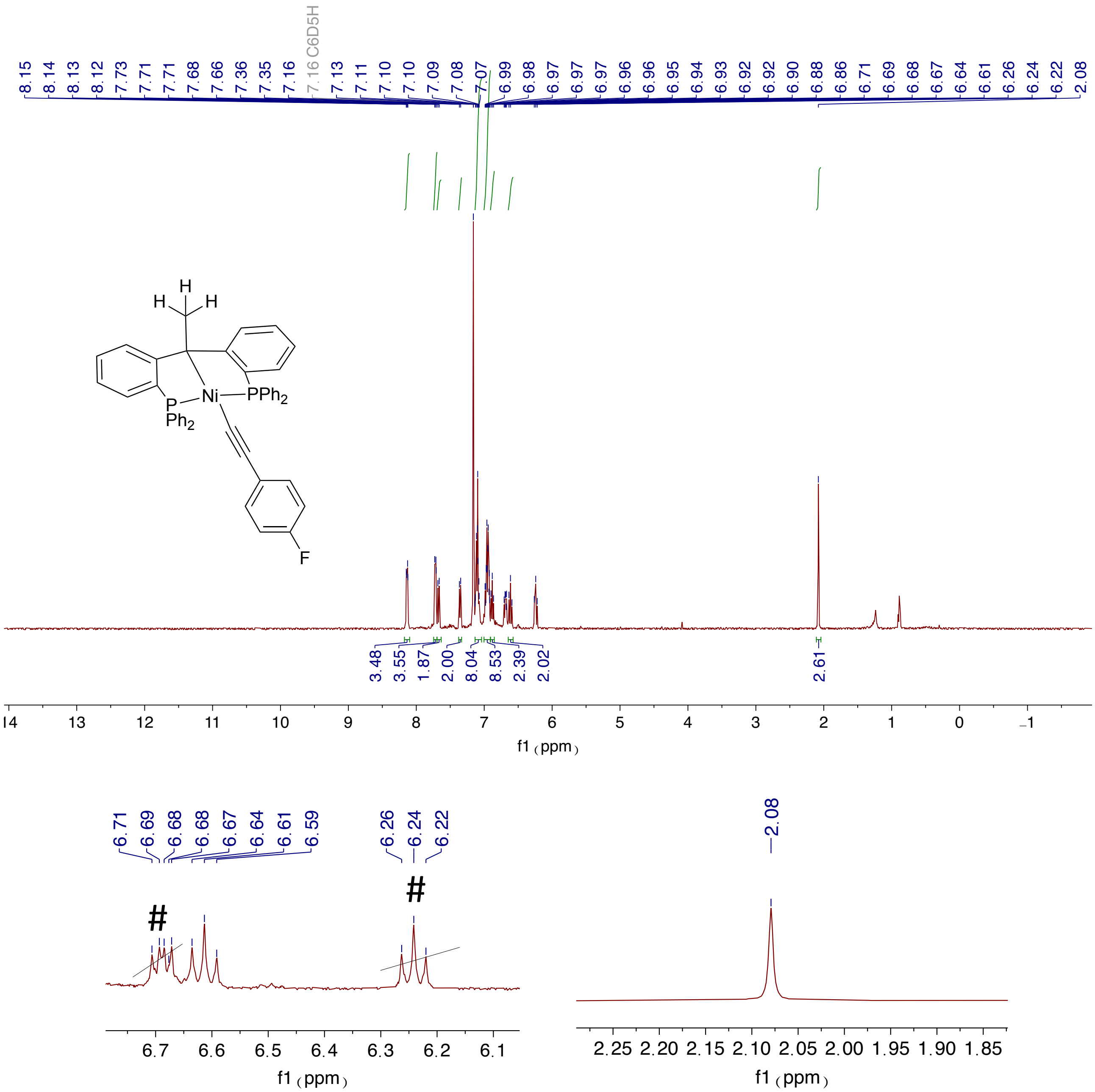

Figure S53. $^{1}H\{^{31}P\}$ NMR spectrum of complex **10** in C_6D_6 at 25 °C. Crossed peaks marked with (#) correspond to free 4-fluorobenzonitrile.

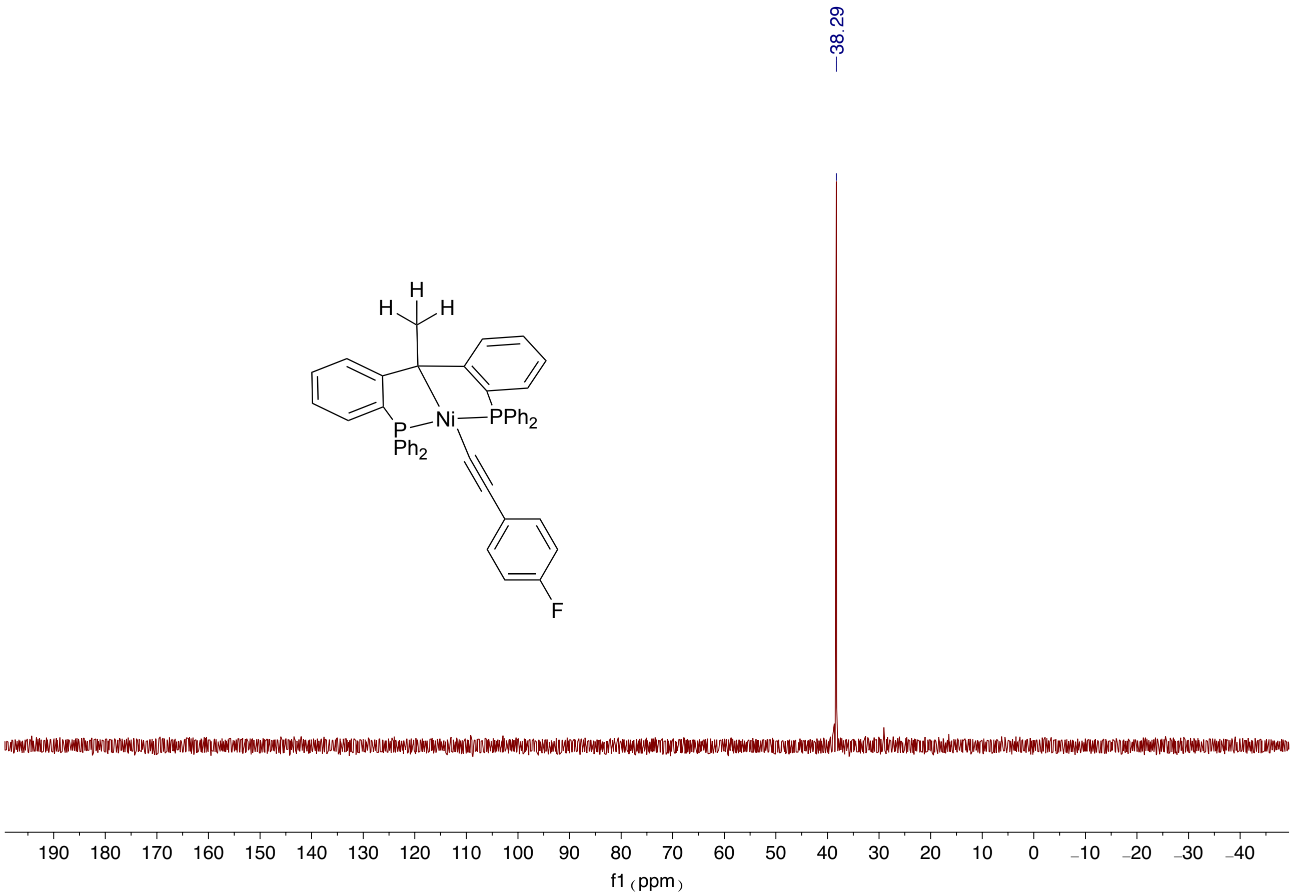

Figure S54. ^{31}P NMR spectrum of complex **10** in C_6D_6 at 25 °C.

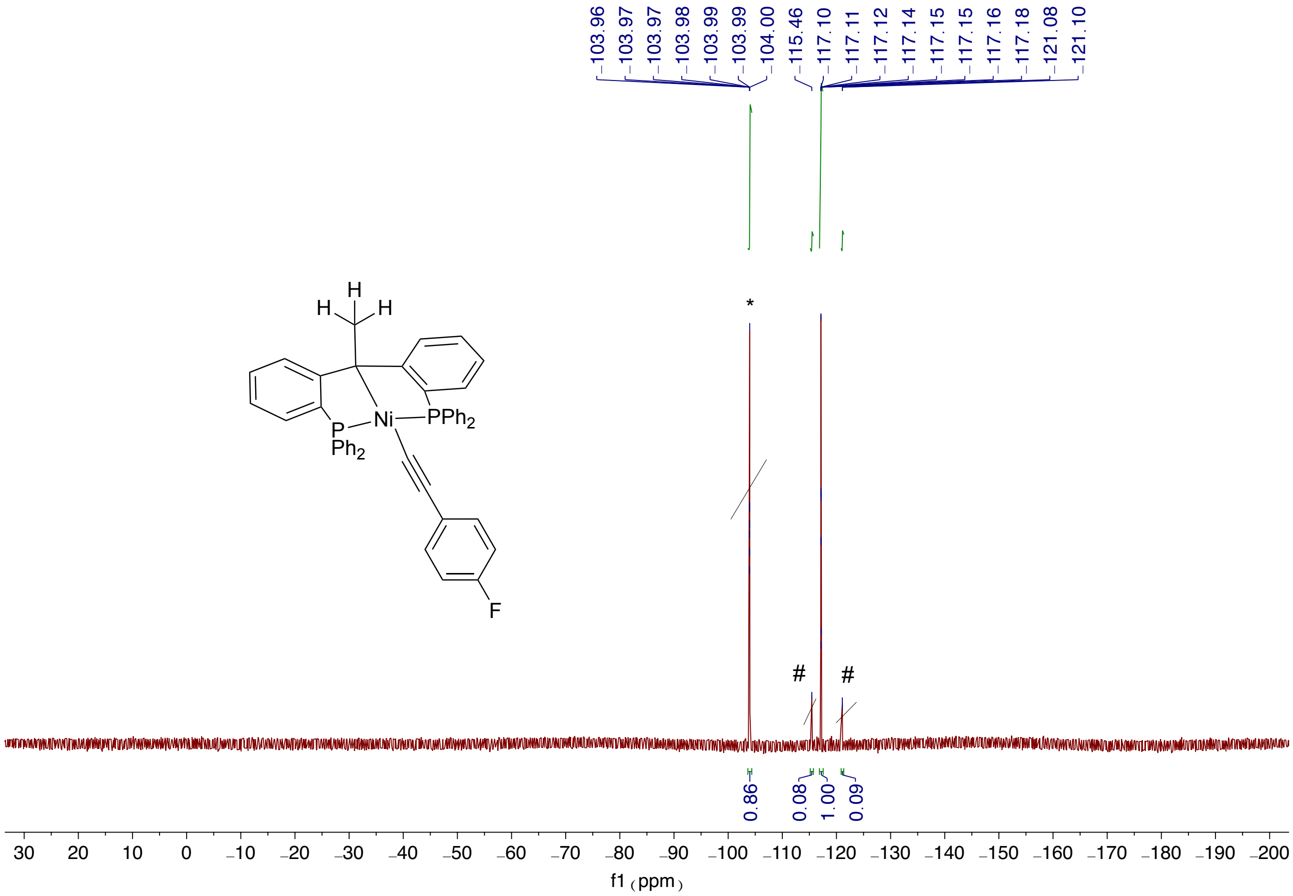

Figure S55. ^{19}F NMR spectrum of complex **10** in C_6D_6 at 25 ºC. Crossed peak marked with (*) correspond to free 4-fluorobenzonitrile. Crossed peaks marked with (#) correspond to a small quantity of complex **7**.

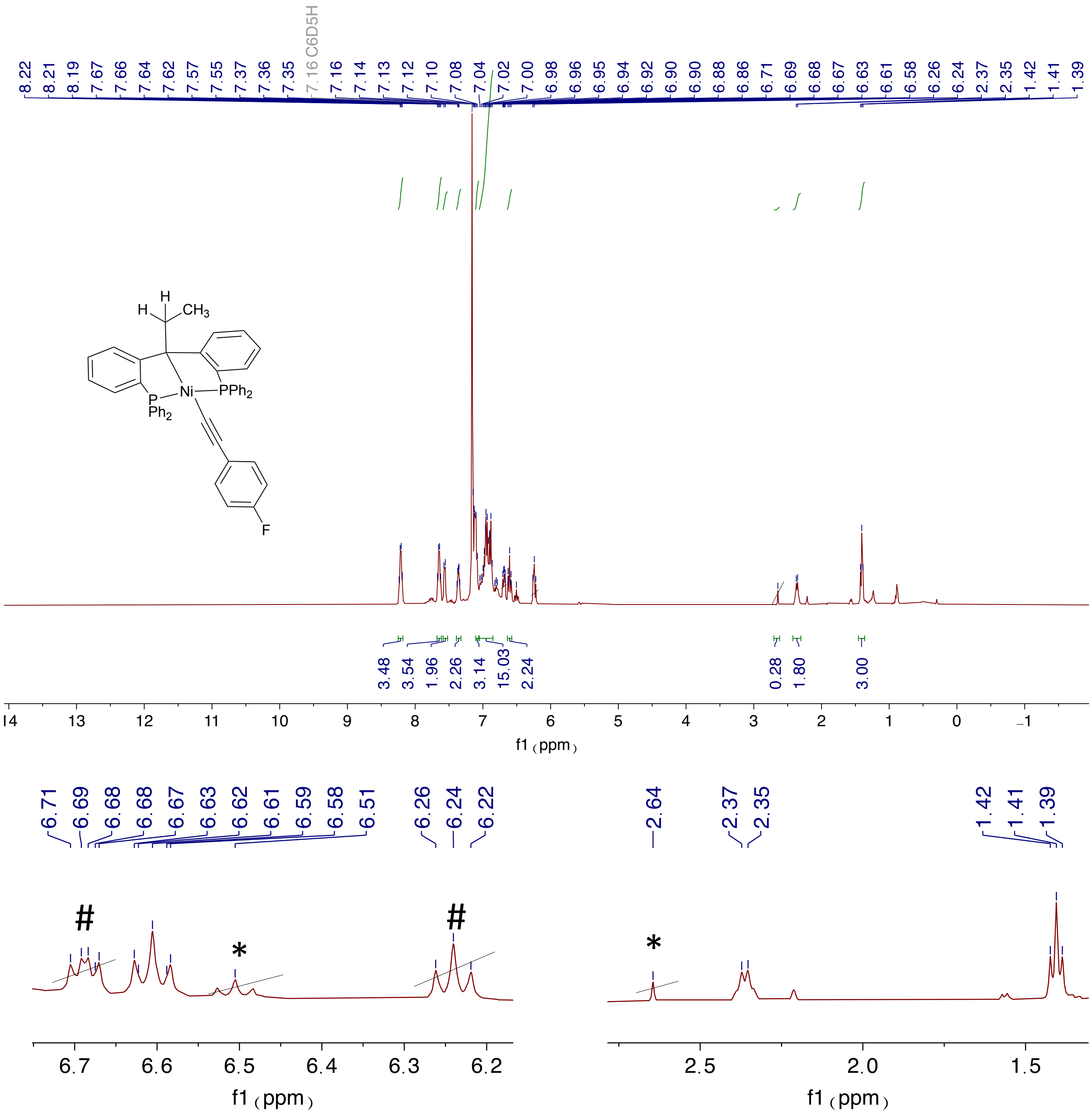

Figure S56. ^{1}H NMR spectrum of complex **11** in C_6D_6 at 25 ºC. Crossed peaks marked with (#) correspond to free 4-fluorobenzonitrile. Crossed peaks marked with (*) correspond to free 1-ehtynyl-4-fluorobenzene.

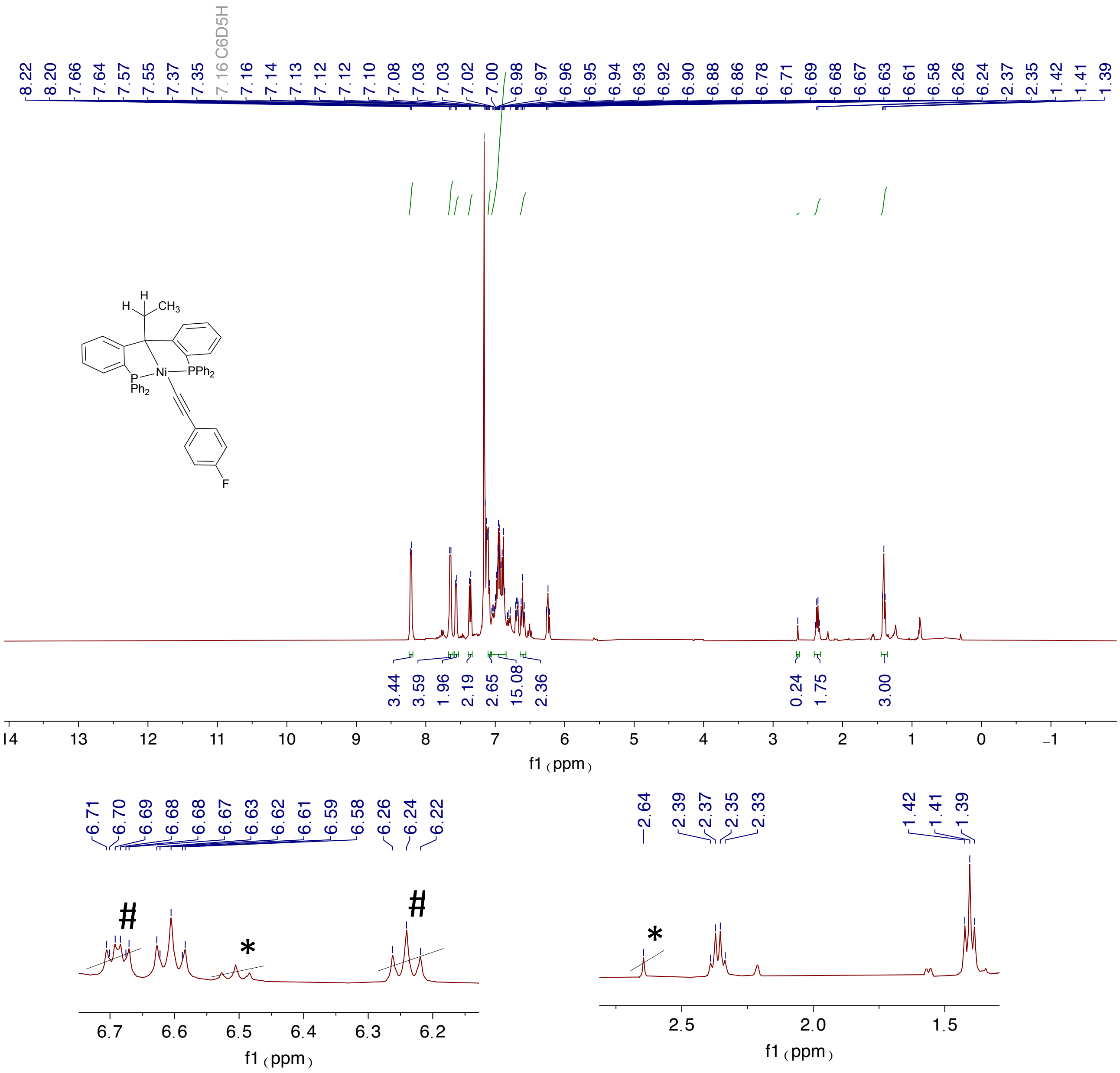

Figure S57. $^{1}H\{^{31}P\}$ NMR spectrum of complex **11** in C_6D_6 at 25 ºC. Crossed peaks marked with (#) correspond to free 4-fluorobenzonitrile. Crossed peaks marked with (*) correspond to free 1-ehtynyl-4-fluorobenzene.

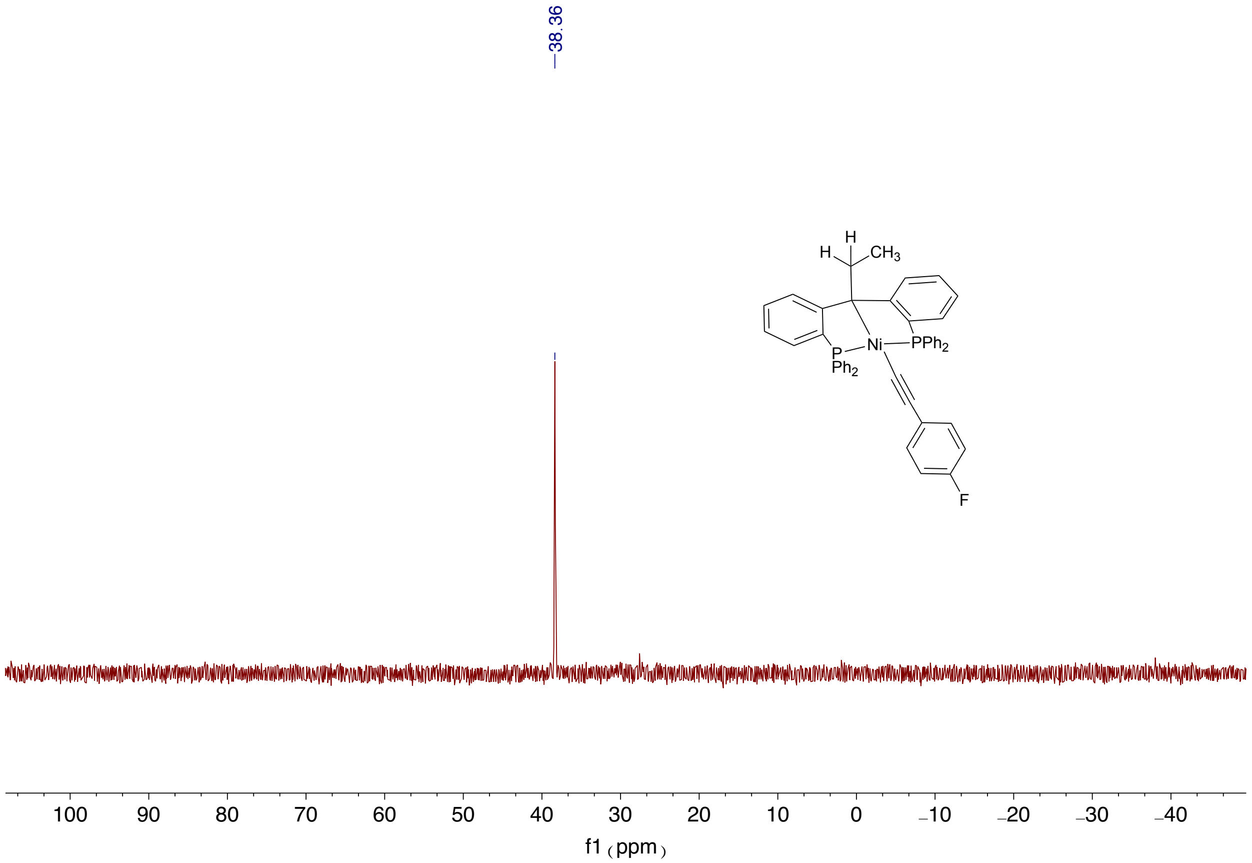

Figure S58. $^{31}P\{^{1}H\}$ NMR spectrum of complex **11** in C_6D_6 at 25 ºC.

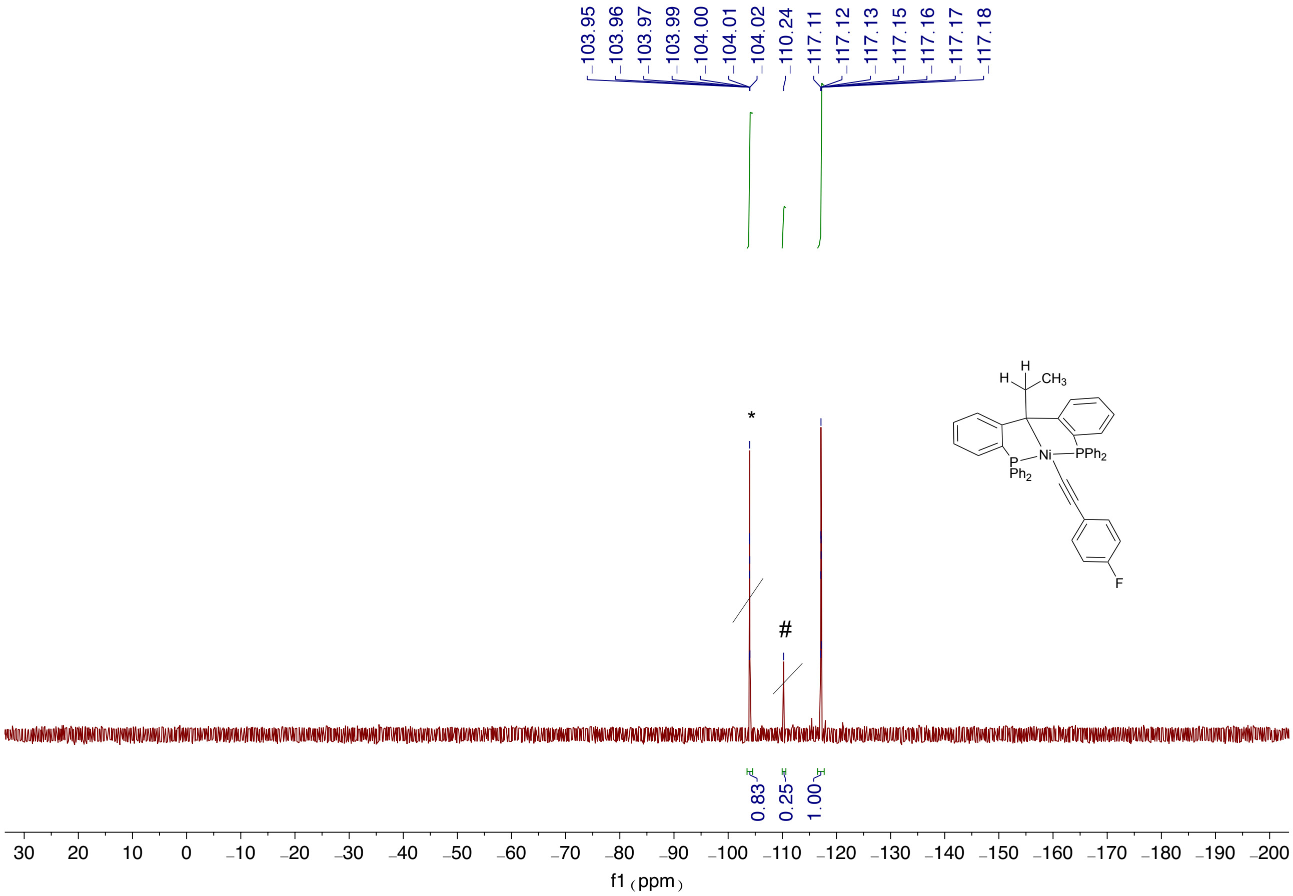

Figure S59. ^{19}F NMR spectrum of complex **11** in C_6D_6 at 25 ºC. Crossed peak marked with (*) correspond to free 4-fluorobenzonitrile. Crossed peaks marked with (#) correspond to free 1-ethynyl-4-fluorobenzene.

3. X-ray crystal structure determination of complex 8

$C_{56}H_{46}NiO_2P_2 \cdot 2(C_4H_8O)$, Fw = 1015.78, red needle, 0.19 × 0.09 × 0.06 mm³, triclinic, $P\overline{1}$ (no. 2), a = 9.7558(4), b = 15.7936(9), c = 17.5772(9) Å, α = 87.998(3), β = 77.256(2), γ = 84.980(2) °, V = 2631.1(2) Å³, Z = 2, D_x = 1.282 g/cm³, μ = 0.48 mm⁻¹. The diffraction experiment was performed on a Bruker Kappa ApexII diffractometer with sealed tube and Triumph monochromator (λ = 0.71073 Å) at a temperature of 150(2) K up to a resolution of $(\sin \theta/\lambda)_{max}$ = 0.65 Å⁻¹. Intensity integration was performed with the Eval15 software[1]. A multi-scan absorption correction and scaling was performed with SADABS[2] (correction range 0.60-0.75). A total of 38908 reflections was measured, 12111 reflections were unique (R_{int} = 0.065), 7781 reflections were observed [I>2σ(I)]. The structure was solved with Patterson superposition methods using SHELXT.[3] Structure refinement was performed with SHELXL-2018[4] on F^2 of all reflections. Non-hydrogen atoms were refined freely with anisotropic displacement parameters. The THF solvent molecules were refined with a disorder model. The disorder could not be fully resolved. Hydrogen atoms of the nickel complex were located in difference Fourier maps and hydrogen atoms of the THF were introduced in calculated positions. All hydrogen atoms were refined with a riding model. 734 Parameters were refined with 282 restraints (distances, angles and displacement parameters in the disordered THF). R1/wR2 [I > 2σ(I)]: 0.0557 / 0.1342. R1/wR2 [all refl.]: 0.1014 / 0.1533. S = 1.019. Residual electron density between -0.62 and 0.77 e/Å³. Geometry calculations and checking for higher symmetry was performed with the PLATON program.[5]

CCDC 2294468 contains the supplementary crystallographic data for this paper. These data can be obtained free of charge from The Cambridge Crystallographic Data Centre via www.ccdc.cam.ac.uk/data_request/cif.

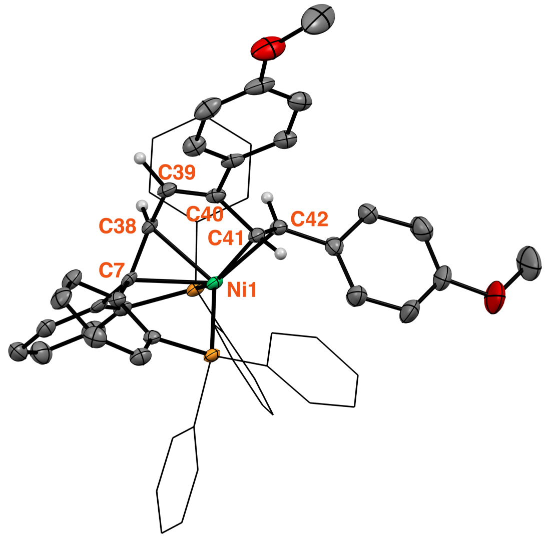

Figure S60. Molecular structure of complex **8**. Displacement ellipsoids are drawn at the 50% probability level. Solvent molecules, most H atoms are omitted for clarity. Phenyl rings of the phosphines and tolyl groups are presented as wireframes. Selected bond lengths (Å): C7–C38 1.413(4), C38–C39 1.468(4), C39–C40 1.332(4), C40–C41 1.496(4), C41–C42 1.398(4), Ni1–C7 2.109(3), Ni1–C38 2.102(3), Ni1–C41 2.022(3), Ni1–C42 2.065(3).

4. Computational studies

4.1 Additional comments about C–H activation mechanism

C–H activation could proceed through two different pathways: oxidative addition/hydride insertion or Ligand-to-ligand hydrogen transfer (
Scheme S3). Both transition states showed significant differences that are summarize in Figure S61.

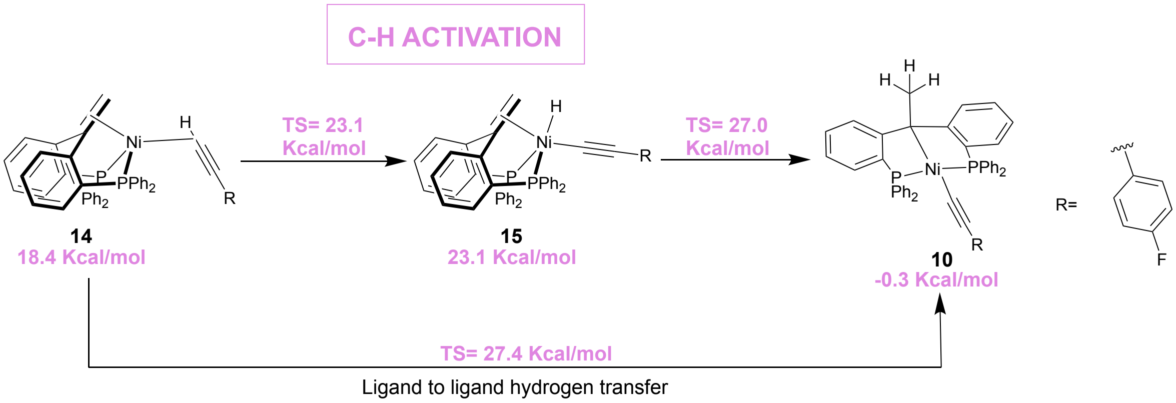

Scheme S3. C–H activation of 1-ethynyl-4-fluorobenzene.

TS 14 to 15, oxidative addition, imaginary frequency: -430.97 cm-

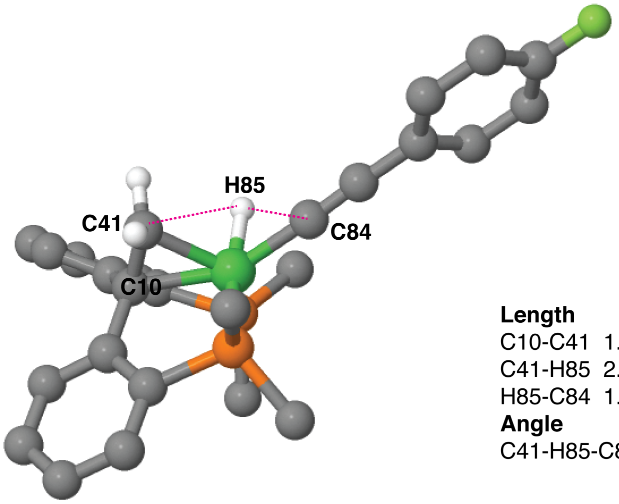

Length
C10-C41 1.413 Å
C41-H85 2.408 Å
H85-C84 1.700 Å
Angle
C41-H85-C84 104.6°

TS 14 to 10, LLHT, imaginary frequency: -1145.93 cm-

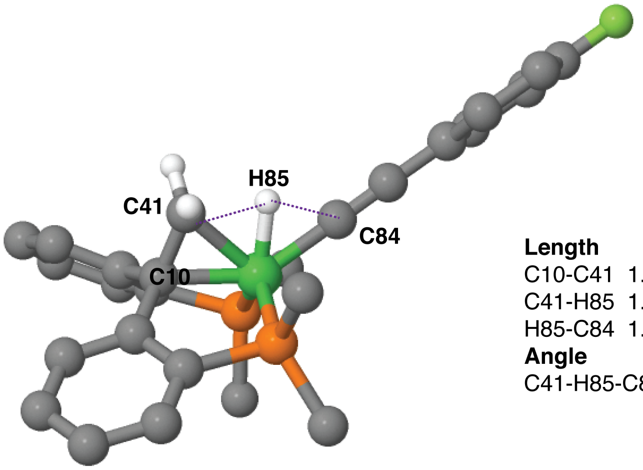

Length
C10-C41 1.451 Å
C41-H85 1.631 Å
H85-C84 1.524 Å
Angle
C41-H85-C84 157.9°

Figure S61. Comparison between transition states of C–H activation.

4.2 Additional comments about the alkene/alkyne coupling

In Figure S62 is presented a detailed Gibbs energy profile for the formation of complex **7** via alkene/alkyne coupling, in black is presented the proposed route for the formation of complex **7**. Other conformers result of the decoordination of a phosphine arm are presented in blue, their calculated energy is higher or similar to the conformers with both phosphines arms coordinated (black). Therefore, pathways involving these intemediates were not computed. Additionally, a route of coupling from complex **18-1-noP** was computed and showed to be higher than the coupling from complex **18**. This is in good agreement with the experimental observations.

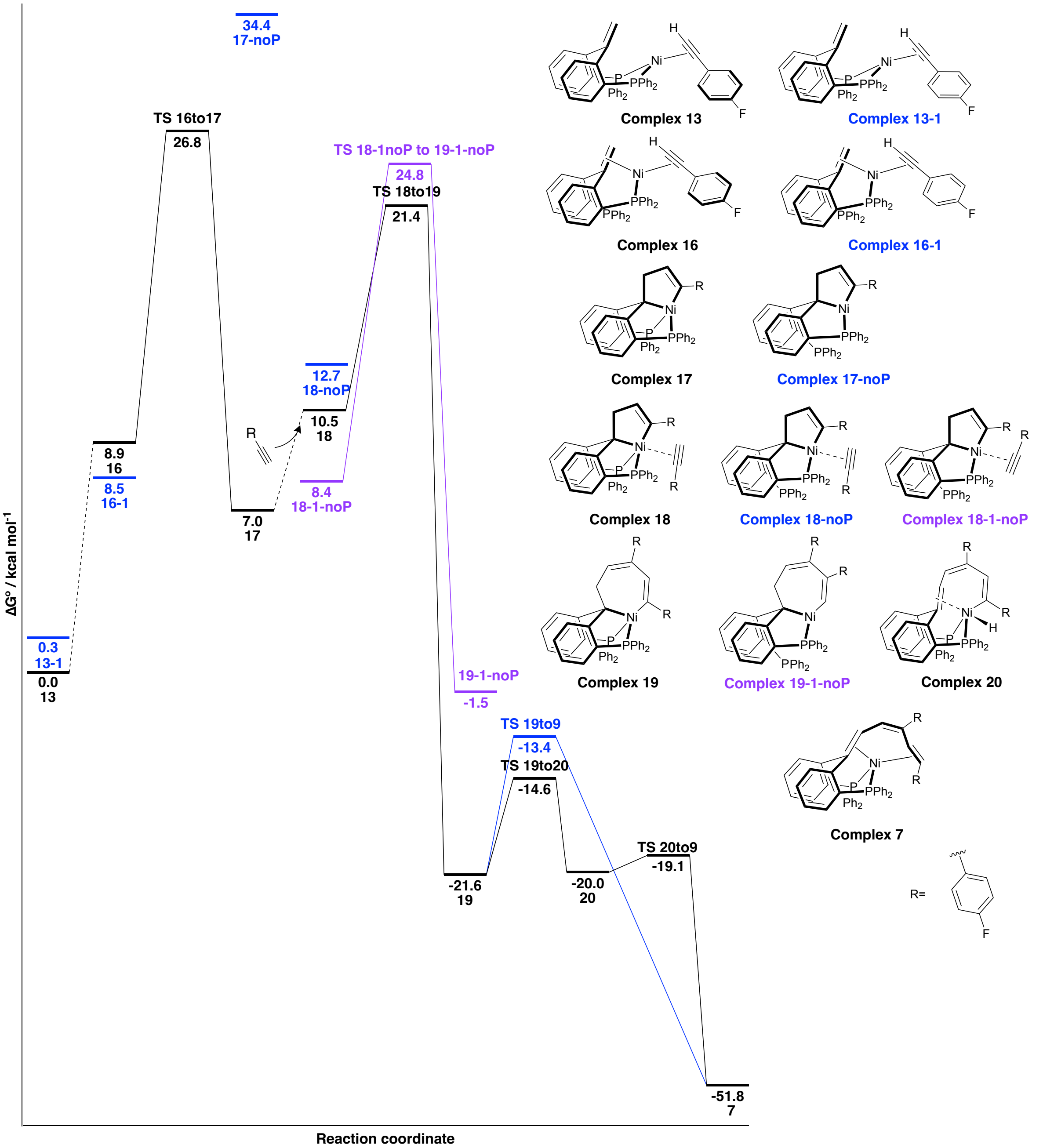

Figure S62. Detailed alkene/alkyne coupling mechanism and additional isomers.

As discussed, the formation of **7** could go through two different pathways: β-hydride elimination/reductive elimination or ligand-to-ligand hydrogen transfer. The energy difference between these processes does not indicate a significant preference for one pathway. Both transition states are similar but present important differences in the bond distances and angles as shown in Figure S63.

TS 19 to 9, LLHT
Imaginary frequency: -892.76 cm-

TS 19 to 20, β-hydride elimination
Imaginary frequency: -784.31 cm-

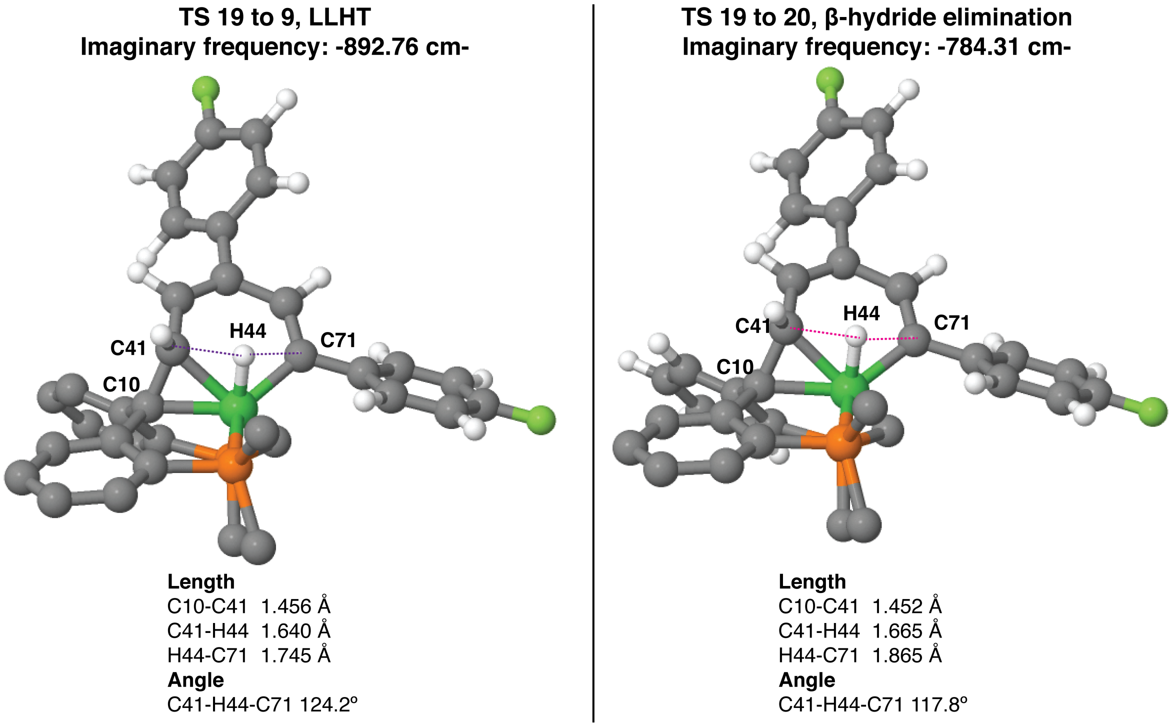

Length
C10-C41 1.456 Å
C41-H44 1.640 Å
H44-C71 1.745 Å
Angle
C41-H44-C71 124.2°

Length
C10-C41 1.452 Å
C41-H44 1.665 Å
H44-C71 1.865 Å
Angle
C41-H44-C71 117.8°

Figure S63. Comparison between β-hydride elimination vs. LLHT from complex 18.

4.3 Comparison nickelacyclopentadiene intermediate

An alternative pathway starting from the coupling of two alkyne molecules was computed (Figure S64). Starting from complex **13**, the coordination of a second molecule of alkyne towards complex **21** is endergonic by 15.4 Kcal/mol. The decoordination of one phosphine arm leads to a tricoordinated complex **21-noP** (21.5 Kcal/mol). **TS 21-noPto21**: alkyne/alkyne coupling takes place with an overall barrier of 38.8 Kcal/mol yielding complex **21**, showing that this process is not competitive. No transition state from complex **21** to **22** (without prior phosphine arm decoordination) was found. Additionally, another isomer (complex **21-1**) was computed but its formation was found to be more endergonic than complex **20.**

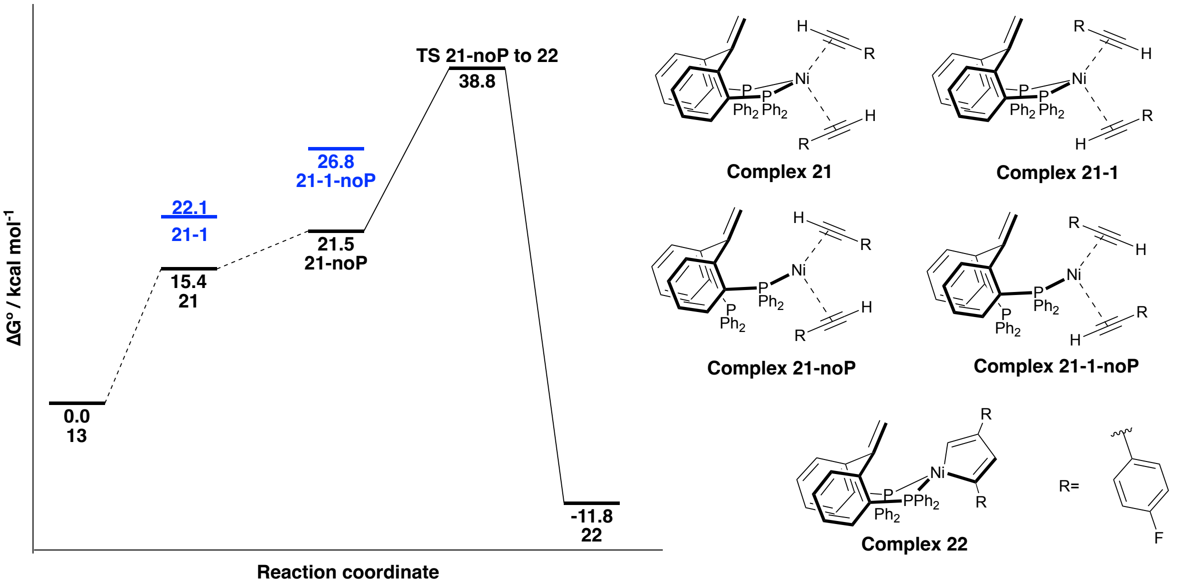

Figure S64. Alkyne-alkyne coupling pathway.

4.4 Table of energies

	Energy (Hartree)	Thermal correction to free energy
**1-ethynyl-4-fluorobenzene**	-407.8266578	0.070155
**4-Fluorobenzonitrile**	-423.9280892	0.059481
**Complex 5**	-4082.020629	0.573136
**Complex 7**	-4473.879682	0.689402
**Complex 10**	-4065.93545	0.583691
**Complex 12**	-4489.894779	0.668796
**Complex 13**	-4065.937714	0.586525
**Complex 13-1**	-4065.936644	0.585972
**Complex 14**	-4065.90388	0.581973
**Complex 15**	-4065.894353	0.579957
**Complex 16**	-4065.918641	0.581788
**Complex 16-1**	-4065.920017	0.582393
**Complex 17**	-4065.927579	0.587571
**Complex 17 no P**	-4065.882286	0.585886
**Complex 18**	-4473.776361	0.685433
**Complex 18 no P**	-4473.767531	0.680051
**Complex 18-1 no P**	-4473.774692	0.680388
**Complex 19**	-4473.830506	0.688325
**Complex 19-1**	-4473.828659	0.688908
**Complex 19-1 no P**	-4473.792782	0.682741
**Complex 20**	-4473.825834	0.686272
**Complex 21**	-4473.760434	0.677332
**Complex 21-1**	-4473.750524	0.678124
**Complex 21-1 no P**	-4473.740441	0.675545
**Complex 21 no P**	-4473.745886	0.672498
**Complex 22**	-4473.810439	0.684001
**TS 14 to 10**	-4065.886262	0.578758
**TS 14 to 15**	-4065.89395	0.579592
**TS 15 to 10**	-4065.886105	0.577962
**TS 16 to 17 no P**	-4065.877204	0.583269
**TS 16 to 17**	-4065.894927	0.586394
**TS 18 to 19**	-4473.758859	0.685363
**TS 18-1noP to 19-1noP**	-4473.75087	0.682778
**TS 19 to 7**	-4473.811338	0.681816
**TS 19 to 20**	-4473.814675	0.683825
**TS 20 to 7**	-4473.822477	0.684345
**TS 21 no P to 22**	-4473.720847	0.674938

5. References

[1] A. M. M. Schreurs, X. Xian, L. M. J. Kroon-Batenburg. "*EVAL15*: a diffraction data integration method based on *ab initio* predicted profiles". *J. Appl. Cryst.* (2010). **43**, 70-82.
[2] L. Krause, R. Herbst-Irmer, G. M. Sheldrick, D. Stalke. "Comparison of silver and molybdenum microfocus X-ray sources for single-crystal structure determination". *J. Appl. Cryst.* (2015). **48**, 3-10.
[3] G. M. Sheldrick. "*SHELXT* - Integrated space-group and crystal-structure determination". *Acta Cryst.* (2015). A**71**, 3-8.
[4] G. M. Sheldrick. "Crystal structure refinement with *SHELXL*". *Acta Cryst.* (2015). C**71**, 3-8.
[5] A. L. Spek. "Structure validation in chemical crystallography". *Acta Cryst.* (2009). D**65**, 148-155.
